# DeBruyn and Casagrande manuscripts on tree shrew retinal ganglion cells as a basis for cross-species retina research

**DOI:** 10.1017/S0952523821000171

**Published:** 2022-01-31

**Authors:** Thomas T. Norton, Elise L. Savier, Madineh Sedigh-Sarvestani

**Affiliations:** 1 Department of Optometry and Vision Science, University of Alabama at Birmingham, Birmingham, Alabama, USA; 2 Department of Biology, University of Virginia, Charlottesville, Virginia, USA; 3 Functional Architecture and Development of Cerebral Cortex, Max Planck Florida Institute for Neuroscience, Jupiter, Florida, USA

**Keywords:** cell density, visual streak, *area centralis*, central projections, soma size

## Abstract

The purpose of this brief communication is to make publicly available three unpublished manuscripts on the organization of retinal ganglion cells in the tree shrew. The manuscripts were authored in 1986 by Dr. Edward DeBruyn, a PhD student in the laboratory of the late Dr. Vivien Casagrande at Vanderbilt University. As diurnal animals closely related to primates, tree shrews are ideally suited for comparative analyses of visual structures including the retina. We hope that providing this basic information in a citable form inspires other groups to pursue further characterization of the tree shrew retina using modern techniques.

Northern tree shrews (*Tupaia belangeri* or *Tupaia chinensis*) (Fig. 1A) are small (150–200 g) diurnal animals that are closely related to primates (Fig. 1B). They are dichromatic, have cone-dominated retinas, a well-developed visual system, and exhibit complex visually guided movements. Tree shrews have been used by the visual neuroscience community since the 1960s. In the decades since, a small cohort of investigators have studied various aspects of the development, structure, and function of the tree shrew visual system. Their discoveries have produced significant advances in our understanding of vision, leading to a growing interest in the use of tree shrews as model organisms in neuroscience. However, in contrast to the relatively detailed characterization of cortical and subcortical visual areas, published information about the first stage of the tree shrew visual system, the retina, and the output projections of the retinal ganglion cells (RGCs) remains sparse.

**Fig. 1.**
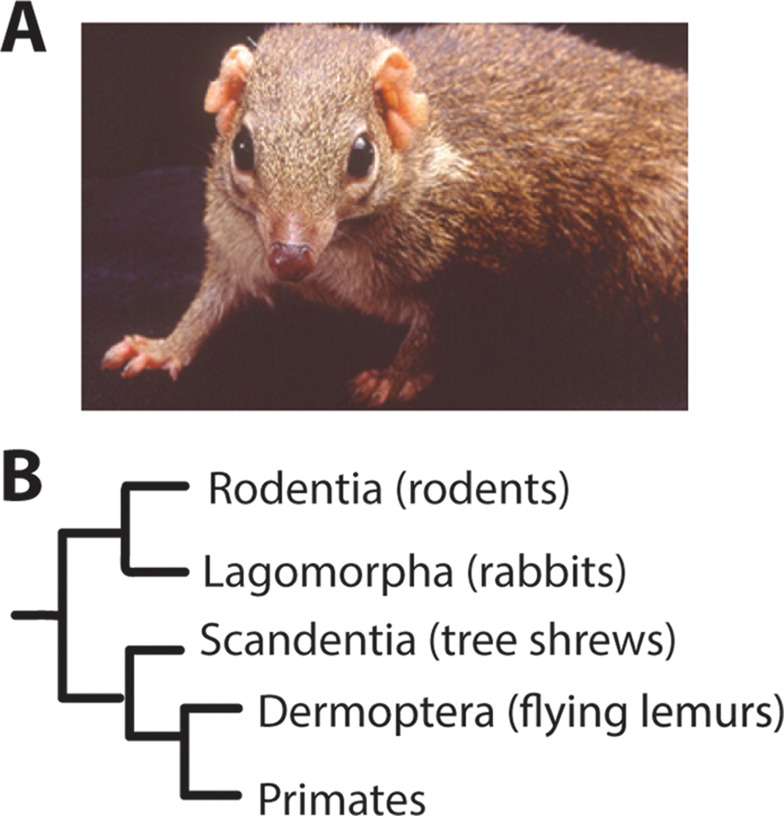
The northern tree shrew. (**A**) An adult animal. (**B**) Phylogenetic position of tree shrews and primates (simplified from Janecka et al., 2007).

In the early 1980s, Dr. Edward DeBruyn, then a PhD student in the laboratory of Dr. Vivien Casagrande, produced a characterization of the organization, morphology, and projections of RGCs in the tree shrew retina. The findings were documented in the first three chapters of Dr. DeBruyn’s doctoral thesis (DeBruyn, 1983). Manuscripts based on these chapters were submitted for publication in 1986 and were returned for revisions that were not made. Thus, the work has remained unpublished (DeBruyn & Casagrande, 1986*a*,*b*; DeBruyn et al., 1986). A few copies were distributed to colleagues by Dr. Casagrande before her death in 2017. Since then, the first author of this brief communication has circulated copies to interested researchers who had become aware of the studies by word of mouth.

The purpose of this communication is to make these unpublished manuscripts on the organization of RGCs in the tree shrew available to the neuroscience community. Here we briefly describe the central findings of the works, place them in the context of current knowledge of cross-species retinal properties, and refer the reader to the three manuscripts in Supplementary Material. Although they did not complete the peer-review process, the manuscripts contain the majority of our current morphological knowledge about tree shrew RGCs. We hope their availability inspires future studies using modern techniques that may confirm or refine the main findings. To cite any of these manuscripts, please follow the format used in this brief communication both for in-text citations and for the full reference as listed in the reference section.

## Manuscript I: Organization of RGCs in the tree shrew: Analysis of cell size distribution

**Fig. 2.**
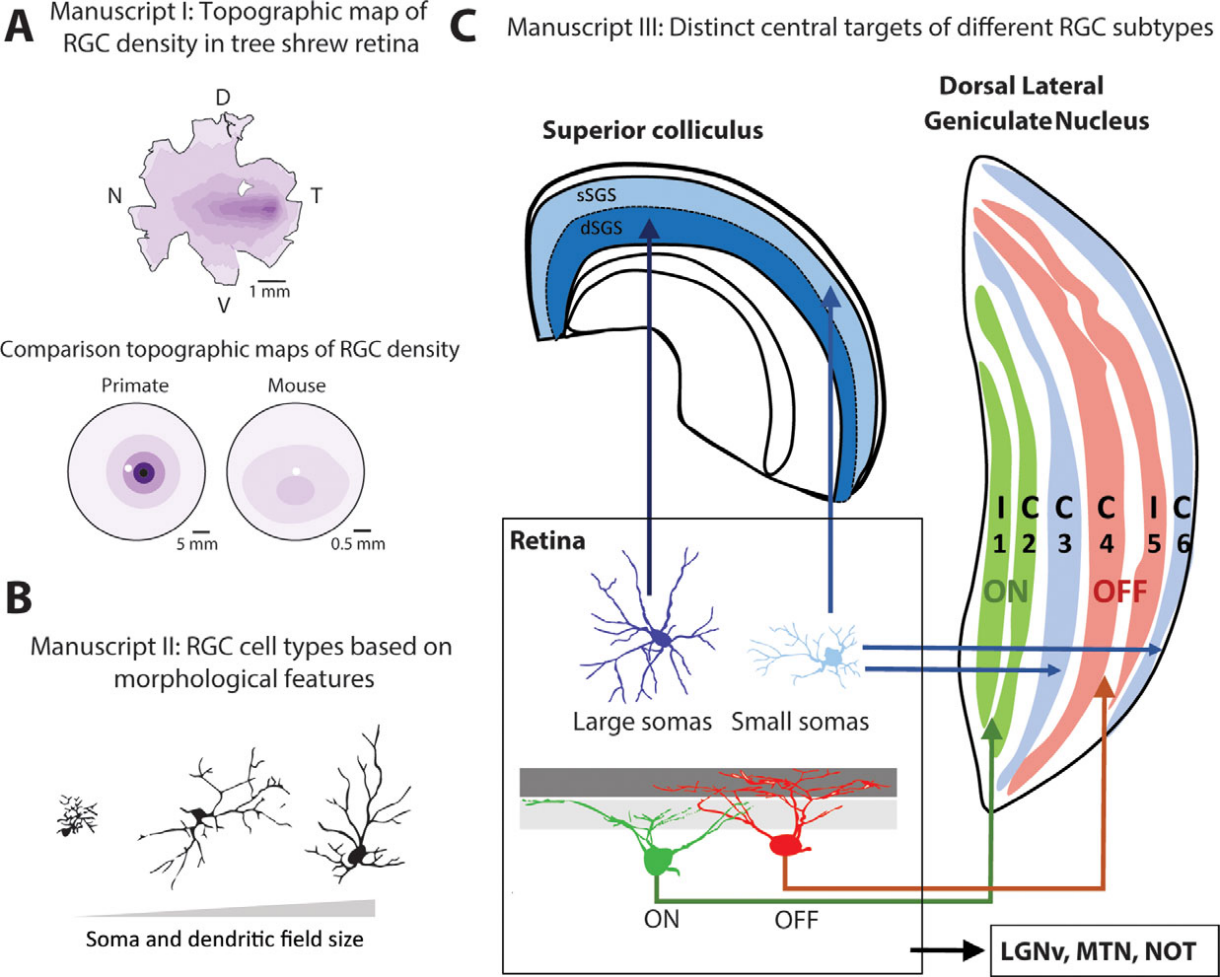
The manuscripts provide a preliminary sketch of the organization of RGCs in the tree shrew retina. (**A**) Manuscript I provides the topographic map of RGC density in tree shrews. Topographic maps from primate (adapted from Heukamp et al., 2020) and mouse are provided for comparison. Maximum RGC density is 20,000 cells/mm^2^ for tree shrews, 60,000 cells/mm^2^ for primates (macaques) (Wässle et al 1990) and 8,000 cells/mm^2^ for mice (Heukamp et al., 2020). Dark purple indicates increased cell density. The small white region in each represents the optic disk. (**B**) Manuscript II provides a classification of RGC cell types based on somatic and dendritic-field sizes and other morphological features. (**C**) Manuscript III identifies central projections of different RGC cell types. Laminae 1 and 2 contain ON-center neurons; laminae 4 and 5 contain OFF-center cells. Lamina 3 contains a mixture of cells with ON-centers or OFF-centers while lamina 6 contains mostly cells with ON–OFF-centers and suppressive surrounds (Holdefer & Norton, 1995). C, contralateral eye input; D, dorsal; dSGS, deep stratum griseum superficiale; I, ipsilateral eye input; LGNv, ventral lateral geniculate nucleus; MTN, medial terminal nucleus; N, nasal; NOT, nucleus of the optic tract; sSGS, superficial stratum griesum superficiale; T, temporal; V, ventral.

This study provides the first characterization of RGC density distribution across whole-mounted retinas in the tree shrew. The tree shrew retina consists of a “visual streak,” a horizontal elliptical band of densely packed RGCs, and an “*area centralis*,” a small region in the temporal retina with the maximum cell density of approximately 20,000 cells/mm^2^ (Fig. 2A). Such a density distribution suggests that the tree shrew retina is specialized to process detailed features in both the central and peripheral visual fields. This is markedly different from the concentric foveal specialization in the retina of cats and primates that produces high-acuity vision in a small region of the central visual field but much lower acuity vision in the periphery. Retinal visual acuity in tree shrews is also distinctly better than in rodents, who exhibit low-acuity vision across both the central and peripheral visual field despite relatively higher-acuity vision in the upper visual field (Fig. 2A). More recently, the overall organization of the tree shrew retina has been assessed using noninvasive (Sajdak et al., 2019) and histological approaches (Müller & Peichl, 1991; Abbott et al., 2009), however a detailed characterization of the distribution of RGCs is still lacking. Future studies are needed to reproduce these results using modern methods and to determine the relationship of bipolar cell and cone density distributions with the RGC distribution.

## Manuscript II: Organization of RGCs in the tree shrew: Analysis of cell morphology

The second manuscript focuses on morphology (soma size, axon diameter, dendritic-field size, and retinal distribution) of RGCs backfilled with horseradish peroxidase (HRP) injected into the optic tract or superior colliculus (SC). The authors concluded that tree shrew RGCs could be grouped into three major classes based on morphological criteria (Fig. 2B). Though multiple studies have reported 20–30 morphologically defined RGC cell types in the mouse (Sanes & Masland, 2015) such characterization has not been attempted in tree shrews except for this manuscript. Modern techniques, including functional and genetic characterization of RGC cell types in the tree shrew would produce a more accurate picture of the diversity of RGCs in this species as well as their role in vision.

## Manuscript III: Organization of RGCs in the tree shrew: Central projections of RGCs

This manuscript addresses an important question concerning the organization of the visual system: do certain RGC cell types project to specific targets and do all species exhibit the same layout? To answer this question, the authors injected either HRP alone or HRP conjugated to wheat germ agglutinin (WGA-HRP) in different brain structures and analyzed the spread, soma size and dendritic arborization of retrogradely labeled RGCs. These results highlight unique features of the tree shrew visual system, as also reported by other groups including (Fig. 2C): (1) segregation of ON-center (laminae 1 and 2) *versus* OFF-center (laminae 4 and 5) cells into paired ipsi- and contralateral layers in the dorsal lateral geniculate nucleus (LGNd) (Conway & Schiller, 1983; Holdefer & Norton, 1995), (2) presence of separate layers (laminae 3 and 6) of the LGNd dedicated to the koniocellular pathway (Diamond et al., 1993) which provides an opportunity to investigate the contribution of the K-pathway to vision, and (3) the presence of superficial and deep subdivisions of the stratum griseum superficiale (SGS) of the superior colliculus (Albano et al., 1979). It also is worth noting that the koniocellular LGNd layers receive inputs from the superior colliculus. Taken together, these histological results suggest that tree shrew retinal projections show specializations that can be compared with the projections of species both closer to and more distant from primates.

## Future Directions

Although these manuscripts provide fundamental data on the organization of the tree shrew retina, much work remains. For instance, studies of the morphological, functional, and molecular properties of RGC cell types in the mouse, their distribution across the retina, and their contribution to behavior, have produced major advances in our understanding of visual processing in this species. These studies were critically dependent on advances in genetic and molecular tools for the measurement and manipulation of neuronal structure and function such as large-scale electrophysiological recording and single cell RNAseq (Baden et al., 2016; Shekhar & Sanes, 2021). The discovery of new molecular markers has, for example, allowed the identification of the projection pattern of RGC subtypes by using already existing Cre-lines (Martersteck et al., 2017). More recently, the generation of synthetic promoters (Jüttner et al., 2019) and enhancer based viral vector constructs (Hrvatin et al., 2019) has greatly improved the opportunity to extend these approaches to other species, including tree shrews. With the information in these manuscript as a starting place, applying modern tools to tree shrew retina would not only elucidate the species-common and species-specific aspects of the projection pathways from the retina but also would contribute to a common nomenclature for RGC types across species.

Improved understanding of the tree shrew retina will benefit visual neuroscientists as well as the broader community of scientists who study species-specific retinal properties and their relationship to ecological niche and behavior. Given the tree shrew’s proximal position to primates, in the evolutionary tree (Fig. 1B; Janecka et al., 2007) these data also will contribute to our understanding of the evolution of the parallel visual pathways that characterize primate vision.

## Supplementary material

For supplementary material accompanying this paper visit https://doi.org/10.1017/S0952523821000171. click here to view supplementary material

## Supplementary Materials

To view supplementary material for this article, please visit http://doi.org/10.1017/S0952523821000171.
