# Supplementary material for: DeBruyn and Casagrande manuscripts on tree shrew retinal ganglion cells as a basis for cross-species retina research
Source: Vis Neurosci. 2022 Jan 31;39:E001. doi: 10.1017/S0952523821000171 (PMC8807137; doi:10.1017/S0952523821000171)
Supplement: Supplementary file 1 [file vnssup.zip › S0952523821000171sup003.pdf]

The Organization of Retinal Ganglion Cells in the Tree Shrew (*Tupaia belangeri*). III. Central Projections of Different Ganglion Cell Populations

E.J. DeBruyn[1, 2], J.T. Weber[3] and V.A., Casagrande[1, 4]

Departments of Cell Biology[1] and Psychology[4]

Vanderbilt University

Nashville, TN 37232

Department of Anatomy[3]

Tulane University

New Orleans, LA 70112

[2]Present Address: Department of Electrical Engineering

Vanderbilt University

Nashville, TN 37235

Running Head: Tree Shrew Ganglion Cell Projections

Address Correspondence to: Dr. V.A. Casagrande

Department of Cell Biology

Vanderbilt University

Nashville, TN 37232

Key words: Tree shrew, retina, ganglion cells, central projections, lateral geniculate nucleus, superior colliculus.

ABSTRACT

The size distributions, percentages, and in some cases morphological characteristics of retinal ganglion cells projecting to different subcortical targets and their subdivisions in tree shrews (*Tupaia belangeri*) were determined from whole-mounted retinae using the retrograde transport of either horseradish peroxidase (HRP) alone or conjugated to wheat germ agglutinin (WGA-HRP) to identify cells. The major focus of the study compared ganglion cells projecting to the layers of the two main targets of the retina, namely, the dorsal lateral geniculate nucleus (LGNd) and superior colliculus. Following large injections which involved all six layers of the LGNd, 54% of the ganglion cells of all sizes are labeled in the area of densest reaction product contralaterally. Ipsilaterally, 44% of the ganglion cells are labeled. The difference in the proportion of labeled cells is due to a reduction in small ganglion cells. Results of injections restricted to individual layers, or pairs of layers, show that ganglion cells projecting to layers 3 and 6 are, on average, small (l – 34th percentile of local cell sizes) in size, while those projecting to the remaining layers are medium (35 – 74th percentile) and large (75 – 99th percentile) in size. Ganglion cells projecting to the two layers (1 and 2) known to contain physiologically defined ON-center cells (Conway and Schiller, '83) can, in turn, be distinguished from those projecting to the layers (4 and 5) containing OFF-center cells, based upon the ramification of their dendrites within the inner plexiform layer of the retina; the former ramify closer to the ganglion cell layer than the latter.

Ganglion cells projecting to the two physiologically and morphologically distinct subdivisions of superficial grey layer (stratum griseum superficiale, SGS) of the superior colliculus, also form distinct populations (Albano, et al., '78; Graham and Casagrande, '80). In cases involving both subdivisions of the SGS, an average of 54% of the ganglion cells of all sizes, and almost all previously defined morphological types (DeBruyn and Casagrande, '86b), are labeled contralaterally; in no case did we observe labeled cells in the ipsilateral retina. Results of cases with more restricted injections show that the superficial subdivision of the SGS receives input mainly from small ganglion cells while the deep subdivision of the SGS receives input mainly from the largest ganglion cells. Ganglion cells projecting to both the superficial and deep subdivisions of the SGS representing the contralateral hemifield can, in turn,

be distinguished from those projecting to the dorsal cap of the colliculus representing the ipsilateral nasal field: the latter appear to be mainly medium in size.

We also analyzed the size distribution of ganglion cells projecting to four other retinal targets. Results show that the lateral and medial terminal nuclei of the accessory optic system receive input from large ganglion cells, the pretectum principally from medium and large ganglion cells, and the ventral lateral geniculate nucleus mainly from small and large ganglion cells. As with the LGNd and colliculus, the size distribution and percentages of labeled ganglion cells projecting to these targets varies depending upon which subdivisions of each of these areas are involved and whether the projections originate in nasal or temporal retina.

These results, combined with our analysis of the density, distribution, size, and morphological types of retinal ganglion cells (DeBruyn and Casagrande, '86a, b), suggest that, although some classes of ganglion cells clearly innervate more than one central target, each subcortical nucleus, subdivision, or layer receives projections from a different subset of ganglion cell classes and, thus, is provided with different visual information.

INTRODUCTION

In an earlier communication (DeBruyn and Casagrande, '86b), we showed that tree shrew retinal ganglion cells can be subdivided into three major types (I, II, III) which, respectively, share a number of features in common with alpha, beta, and gamma cells in cat retina (Boycott and Wässle, '74). Like cat ganglion cells, tree shrew ganglion cell classes show rough division by size; type I cells are, on average, larger than type II cells which, in turn, tend to be larger than type III cells. In tree shrews these three morphological cell types can be subdivided further into five classes or clusters based upon a quantitative cluster analysis. Comparisons between the three types and five clusters suggest that clusters 1 and 2 subdivide type I cells, cluster 3 and type II cells refer to an almost identical population of cells and clusters 4 and 5 roughly subdivide type III cells. By comparing the distribution and morphological characteristics of ganglion cell classes in tree shrews with identified physiological classes (Van Dongen et al., '76; Ter Laak and Thijssen, '78), we were able to argue for certain correlations, the most obvious being between a subdivision of the morphological type I cells (cluster 2 cells) and the physiological Y-like cells, and between the morphological type II cells (or cluster 3 cells) and the physiological X-like cells. Since studies in other species (Rodieck and Brening, '83) have provided evidence that ganglion cells in different morphological/physiological groups project centrally via separate channels that preserve functional differences established in the retina, the major goal of the present study was to determine the central targets of different classes of tree shrew ganglion cells and to compare the results with what is known about the functional organization of these central targets.

The central visual system of tree shrews lends itself well to this goal since major retinal targets are well developed, highly differentiated, and a considerable volume of work has been devoted to their anatomical and physiological organization. For example, the dorsal lateral geniculate nucleus (LGNd) contains six distinct layers that can be grouped according to physiology, morphology, and connections into two pairs of matched layers (1,2 and 4,5) and two additional unmatched layers (3 and 6). The matched layers are matched in the sense that, although they receive input from different eyes, they are otherwise similar in organization; layers 1 and 2 contain medium and large X and YON-center cells and send axons to the upper tier of layer IV of striate cortex, while layers 4 and 5 contain medium and large X

and Y OFF-center cells and send axons to the lower tier of layer IV. The unmatched contralaterally innervated layers (3 and 6) contain mainly small cells with a mixture of ON- and OFF-center (layer 3) or OFF- and ON-/OFF-center (layer 6) W cells, and project to different sublayers of supragranular striate cortex (Norton, pers. commun.; for review see also Casagrande and Brunso-Bechtold, '85). It seems likely, given the laminar differences in the LGNd, that different ganglion cells classes are involved in projections to the different layers.

The tree shrew superior colliculus is also well developed and exhibits a thick superficial grey layer (the main recipient layer of the retina) which can be subdivided into superficial and deep sublayers based upon cell morphology, physiology, and connections. The superficial tier contains small fusiform cells which have small ON/OFF receptive fields (S-R cells), and project heavily to the dorsal and ventral lateral geniculate nuclei. The lower tier contains large cells, with wide radiating dendrites and large receptive fields, many of which are movement sensitive (M-S cells) and which project to the pulvinar nucleus (Albano et al., '78; '79; Graham and Casagrande, '80; Irvin et al., '83). As with the layers of the LGNd, the sublaminar organization of the superior colliculus suggests that different populations of retinal ganglion cells project to the two subdivisions.

Many of the other targets of the retina also are highly developed in tree shrews and show internal specialization. The ventral lateral geniculate nucleus contains at least three layers or subdivisions and the pretectum is made up of five nuclei; each of these subdivisions and nuclei can, in turn, be distinguished by cytoarchitectural and connectional means, (Weber and Harting, '80) and as our results will show, by retinal ganglion cell input.

In the present report we focused our main efforts on making distinctions between ganglion cells projecting to the layers and subdivisions of the main retinal targets, namely, LGNd and superior colliculus. Less effort was devoted to defining differences between ganglion cells projecting to subdivisions of the remaining retinal targets; the ventral lateral geniculate nucleus (LGNv), the medial and lateral terminal nuclei (MTN and LTN), and the pretectal nuclei. Some of the results were the subject of an earlier communication (DeBruyn and Casagrande, '78).

METHODS

The present results are based upon 52 injections of tracer made in 35 tree shrews (*Tupaia belangeri*). Injections contained either horseradish peroxidase (HRP) or horseradish peroxidase conjugated to the lectin wheat germ agglutinin (WGA-HRP) administered under pressure or iontophoretically into subdivisions of nuclei known to receive retinal projections, i.e., the dorsal and ventral lateral geniculate nuclei (LGNd and LGNv), superior colliculus (SC), pretectum (Pt), and the medial and lateral terminal nuclei of the accessary optic system (MTN and LTN).

Surgical procedures

The surgical procedures were similar to those described in detail elsewhere (DeBruyn and Casagrande, '86b). Briefly, the following procedures were employed. For pressure injections each animal was initially anesthetized with pentobarbital (Nembutal, 55 mg/kg) and placed in a stereotaxic frame for surgery. Sites for pressure injections were guided by stereotaxic coordinates; volumes ranging from 0.1 – 1.7 µl of 20% – 50% HRP (Sigma, type IX) or 5% WGA-HRP (courtesy of Dr. Russell Carey) in saline or 2% dimethylsulfoxide were injected over a 15- to 30-minute period using a 5 µl Hamilton syringe equipped with a 30G blunt needle. Following each injection, the needle was allowed to remain in place for an additional 15 minutes to avoid drawing tracer back along the injection tract. In some animals, portions of the posterior or temporal cortex were aspirated to allow direct visualization of the superior colliculus or the optic tract adjacent to the thalamus. In cases where the size of the injection was restricted to single layers or subdivisions, tracers were iontophoresed into target areas using a glass micropipette (tip diameter 20 – 30 *µm*) and applying a pulsed *2 – 3 µ*amp current for 20 – 30 minutes (1 – 1.5/sec: 500 msec duration). As with the pressure injections, the pipette was allowed to remain in the brain an additional 15 minutes following the end of the injection.

In cases where injections were made into individual LGNd laminae, procedures were modified slightly to allow for recording visually evoked responses. The animals were anesthetized with ketamine hydrochloride (125 mg/kg) and dilute (5.0 mg/ml) Nembutal (7.5 mg/kg). A cannula was inserted into the femoral vein through which supplemental doses (2.5 mg/kg) of Nembutal were administered as required

to maintain adequate anesthesia levels. The pupils were dilated with a 1% solution of atropine sulfate and the corneas protected with zero power contact lenses. The LGNd was approached horizontally through the temporal lobe using visually evoked multiunit potentials recorded via parlene coated tungsten electrodes (BAK, impendence 0.7 – 1.6 M at 1 KHz). Specific layers were identified by noting shifts in the ocular dominance of the responses and whether the predominant response was to the onset or offset of a light spot.

In agreement with Conway and Schiller ('83), **we** noted that cells in medial layers (1 and 2) always gave strong ON responses and cells in the lateral layers (4 and 5) gave strong OFF responses to a flashing light. However, unlike Conway and Schiller, we found that cells in layer 3 (confirmed by injection placement) gave weak ON-OFF responses not purely OFF responses. Responses from layer 6, (an extremely narrow layer) were difficult to elicit, but occasionally faint ON-OFF responses were recorded. Following identification of the layer of interest, the electrode was replaced by a glass micropipette, the location of the layer confirmed by recording through the pipette, and iontophoresis was initiated as described above.

Histological Procedures

Following a 48-hour survival period, the animals were deeply anesthetized with Nembutal, the eyes were removed, and the animals were perfused with either 2.5, 4 or 5% buffered glutaraldehyde − 2% paraformaldehyde. The retinae were then removed from the eyes, and immersion fixed in 2.5% glutaraldehyde. Following the fixation, the retinae were briefly rinsed in buffer and reacted either using Hanker-Yates reagent (Hanker et al., '77) or 3'3-diaminobenzidine hydrochloride (DAB, LaVail and LaVail, '74) as a chromagen, whole mounted, dehydrated and coverslipped as described previously (DeBruyn and Casagrande, '86a, b). The brains were allowed to equilibrate in 30% sucrose fixative and then were frozen-sectioned coronally at 52 μm. Every section through the injection site and selected sections through the remainder of brain were reacted with the same procedures used on the retinae. The sections were then mounted, stained with cresyl violet, dehydrated, and coverslipped.

Measures of ganglion cell number and cell density were made within 0.01 mm^2 fields in the densest area of label. Within these fields, outlines of labeled cells were drawn at l000X using a *camera lucida* drawing tube (Zeiss). Later, the retinae were counterstained with cresyl violet and outlines of all unlabeled cells within the same field were drawn (DeBruyn and Casagrande, '86b). Subsequently, percentages of labeled verses unlabeled cells and cell areas were calculated and statistically compared with the aid of the Bioquant II image analysis system (E. Leitz).

Potential Sources of Error

Correct interpretation of results following injections of either HRP or WGA-HRP requires that one be sensitive to some of the problems and limitations of the use of these tracers (see also Bunt et al., '74; Winer, '77; Vanegas et al., '78). Four issues were of special concern to us.

First, many of our pressure injections of HRP resulted in a mismatch between the extent of the injection site and the extent of labeled cells in the retina; the injection sites always appeared to be larger than the area of labeled cells. This problem was not encountered following iontophoretic injections of either HRP or WGA-HRP, presumably due to minimal diffusion. In order to accurately discriminate the effective area of the injection in the pressure injection cases, we analyzed several superior colliculus cases in particular detail. Analysis of these cases had the advantage that the extent of the injection sites could be plotted on a map of the visual field (Lane et al., '71) and then compared to the area of labeled cells in the retina. As reported by others (Jones and Leavitt, '74; Vanegas et al., '78), we confirmed that the effective injection site corresponded only to the area of dense reaction product and did not include the entire extent of the diffuse light brown haze surrounding the denser core (see Fig. 1A).

A second concern was potential misinterpretations due to labelling cut fibers of passage with HRP (Bunt et al. '75). As a result, we resorted to the use of WGA-HRP in situations (e.g., laminar LGN injections) where such labelling could confound interpretation; WGA-HRP does not appear to invade cut axons with the central nervous system (Steindler, '82). In addition, we found that injections of HRP involving both the optic tract and adjacent areas of interest (Fig. 1A), or the optic tract alone, tended to result in a wide scatter of labeled cells in the retina. In contrast, HRP injection sites not involving the

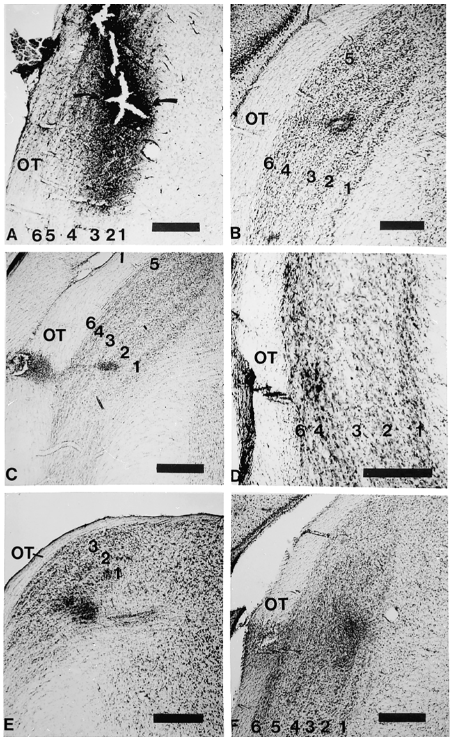

Figure 1. Examples of HRP or WGA-HRP injection sites in the dorsal lateral geniculate nucleus (LGNd) shown in photomicrographs of coronal Nissl counterstained sections from six cases. Examples of the extent of pressure (A) or iontophoretic (B-F) injection sites. A: A larger pressure injection which involved all layers of the nucleus as well as the optic tract. Note the central core of darker reaction product (arrow) which apparently represents the effective injection site (see Methods). B: A small injection which involved layer 3 of the LGNd and part of the interlaminar space between layers 3 and 4. C: A small injection involving layers 2 and 3 of the LGNd. D: A very small injection restricted to layer 4 of the nucleus. E: A large injection centered in the white matter medial to the LGNd but involving layer 1 also. F: A large injection centered in layers 2 and 3, involving all layers except layer 1. Scales = *500µm.* OT, optic tract; numerals refer to the layers of the LGNd.

optic tract (Fig. 1B) resulted in small patches of densely labeled cells (Fig. 2). The most likely interpretation of the above, the position which we take in interpreting the present results, is that the scattered, labeled ganglion cells represent uptake from interrupted optic tract axons and the denser foci of labeled cells represent the pattern of projections to the labeled structure of interest. A related point of concern is with the determination of percentages of labeled ganglion cells projecting to different areas following injections of varying size. It is extremely difficult to be certain that, even in the darkest zones of the injection (the presumptive effective zone), all ganglion cells projecting to that zone will effectively transport the label. Very small injection sites may involve only parts of the axonal arbors of ganglion cells which may not produce a recognizably labeled ganglion cell, resulting in lower estimates of percentages

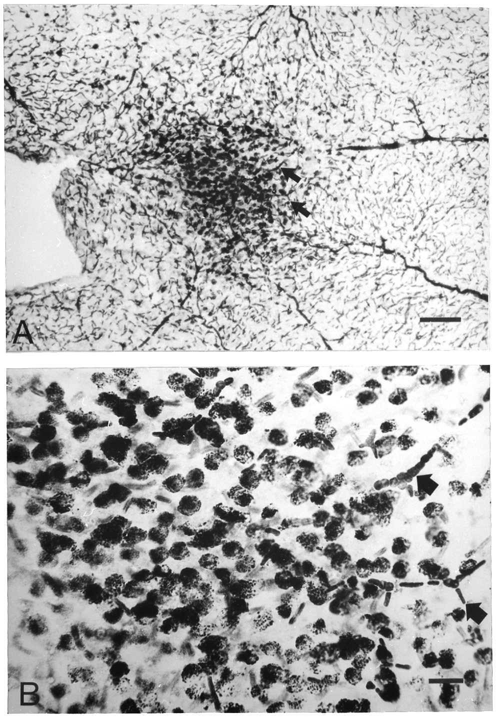

Figure 2: Examples of labeled retinal ganglion cells within the retina. A: A low power photomicrograph of a small patch of labeled cells centered in the *area centralis*. Superior is toward the top and nasal toward the left of this photomicrograph of a retinal whole mount. B: A higher power photomicrograph of the same patch of labeled cells to illustrate the HRP granules within the cell somas. Arrows mark the same blood vessels in each figure. Scales = 100 μm (A); 20 μm (B).

of labeled cells. Therefore, comparison of results concerning the relative percentages of labeled ganglion cells following injections into different retinal targets should be viewed only as rough estimates.

A third point of concern is with interpretation of absolute cell size. As discussed in an earlier communication (DeBruyn and Casagrande, '86a), absolute retinal ganglion cell size measures are almost meaningless since relative size varies with cell density, and, thus, eccentricity. Also, cell size is influenced somewhat by histological treatment and may vary individually between animals (Hughes, '81). Therefore, as in the past we have described the size of labeled cells as a percentile figure relative to the size of the total population of ganglion cells in the area measured.

A final concern, one that has plagued others (Bunt et al., '74), is with the potential selectivity of the label and sensitivity of chromagens used to identify that label. It has been suggested that some small retinal ganglion cells may not transport HRP or may not transport enough of the enzyme to be detectable using less than the most sensitive chromagens. However, since some of our HRP injections labeled the smallest ganglion cells, it seems unlikely that the label itself or our methods discriminate against this

population. On the other hand, since in the majority of our cases, the mean for the unlabeled population was small, we cannot rule out the possibility that some small cells are not labeled due to technical difficulties.

RESULTS

The Dorsal Lateral Geniculate Nucleus (LGNd)

The tree shrew LGNd consists of six cytoarchitectonically distinct layers; two of which receive uncrossed (1 and 5) and four of which received crossed (2, 3, 4, and 6) input. Pressure injections involving the entire LGNd were made in 5 animals; and resulted in labelling a maximum of 56% of the ganglion cells (range = 52.3 – 55.9%).

In case 77-46 the injection included all four layers of the monocular segment (Fig. 3A) and labeled cells were found only within the contralateral retina at a point roughly corresponding to the representation of the dense core of reaction product in the injection site, in the inferior nasal retina (Fig. 3B). Fifty-two percent of ganglion cells in the area of densest reaction product were labeled. Histograms of labeled vs. unlabeled cells (Fig. 3C) show that while labeled cells fall into all size classes, the majority

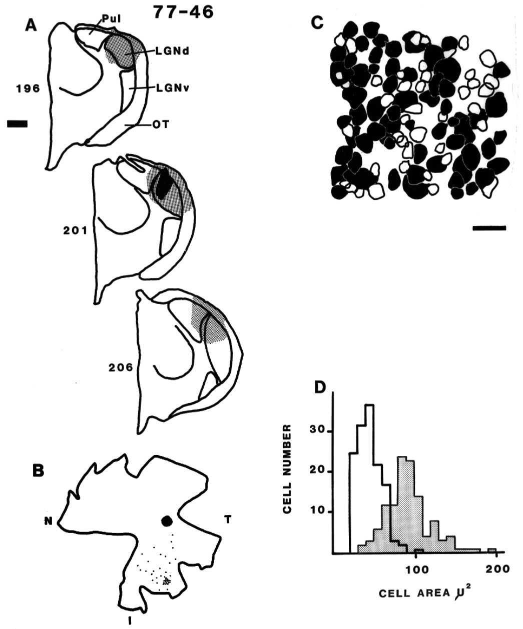

Figure 3: Results of an injection into the monocular segment of LGNd in case 77-46. A: The presumptive effective injection site (black center) involved all the layers (2, 3, 4 and 6) of the monocular segment of the LGNd and part of the optic tract shown in drawings of coronal sections at three levels through the thalamus. B: The topographic distribution of labeled cells in the contralateral retina, depicted by dot density. No labeled cells were found in the ipsilateral retina. C: The appearance of one 0.1 X 0.1 mm field from the area of densest label. Labeled cells are filled, unlabeled cells are open. D: A histogram of labeled (thin lined, filled histogram) vs. unlabeled (thick lined, open histogram) retinal ganglion cells. The scales are 1 mm for the injection site drawings depicted in A, and 20 *μm* for cell drawings depicted in C. I, inferior; N, nasal; T, temporal; LGNd, dorsal lateral geniculate nucleus; LGNv, ventral lateral geniculate nucleus; OT, optic tract; pul, pulvinar; numerals in A refer to section levels.

of labeled cells are medium and large in size relative to the local population with a mean (40.5th percentile ± 1.46 S.E.) in the range of the medium size cells. It is noteworthy that all of the largest cells (top 25% of the size range) of the local population contained label while very few of the smallest cells were labeled.

In case 79-104 the injection site involved the dorso-caudal binocular segment of the nucleus as well as a small portion of the optic tract (Fig. 4A). In addition to the dense patches of labeled cells within the area centralis of both retinae, a random scattering of labeled cells was found in the contralateral retina (Fig. 4B). As noted in the methods, the scattered labeled cells in the contralateral retina likely reflect filling of cut optic tract fibers rather than uptake from the injection site in the LGNd. Examination of the

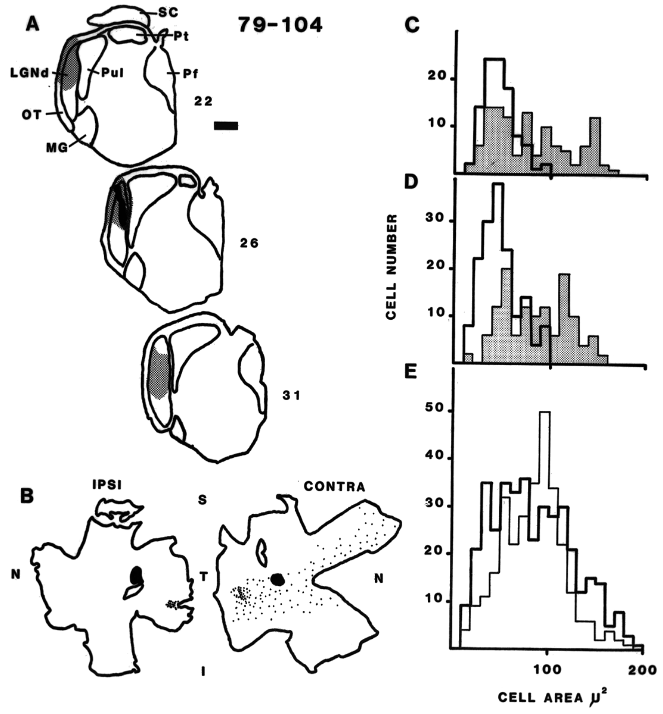

Figure 4: Results of an injection into the binocular segment of the LGNd in case 79-104. A: The presumptive effective injection site involved the dorsocaudal portion of the nucleus and part of the optic tract. B: The topographic distribution of labeled cells in the contralateral (contra) and ipsilateral (ipsi) retinae. The scattered cells throughout the contralateral retina are likely due to optic tract involvement. C: Histograms of labeled vs. unlabeled ganglion cells in the contralateral retina. D: Histograms of labeled vs. unlabeled cells in the ipsilateral retina. This distribution is similar to that seen in the contralateral retina, with the exception that fewer small ganglion cells are labeled. Again, all large ganglion cells are labeled. E: A comparison of the size distributions of labeled cells from the contralateral (heavy line) and ipsilateral (light line) retina. Note that although the distributions overlap almost completely, there are more small ganglion cells labeled in the contralateral retina. I, inferior; N, nasal; S, superior; T, temporal, MG, medial geniculate nucleus; Pf, parafascicular nucleus; Pt, pretectum; SC, superior colliculus; other abbreviations and conventions as in Figure 3.

frequency histogram of the size of labeled cells in the contralateral retina revealed that contralaterally innervated LGNd layers (2, 3, 4 and 6) in the binocular segment receive projections from approximately 54% of ganglion cells of all sizes (Fig. 4C), although the distribution of labeled cells (mean - 39.9th percentile ± 3.48 S.E.) appears broader and involves more of the smallest cells than in the previous case. It is unclear whether the difference in the labeled versus unlabeled distributions of ganglion cells projecting to the monocular and binocular segments reflects real differences in the proportion of morphological classes projecting to the two LGNd divisions or is simply a reflection of differences in injection size or involvement of different LGNd layers (see also below).

In the ipsilateral retina of case 79-104, the histogram (Fig. 4D) shows a distribution of labeled cells (mean - 44.9th percentile ± 2.46 S.E.) similar to that seen in the contralateral retina, with the exception that fewer small ganglion cells are labeled and fewer cells (44%) are labeled overall. When the size distributions of labeled cells from both retinae are compared (Fig. 4E), it is evident that, with the exception of the smallest cells, they overlap almost completely. The additional small cells labeled following injections involving all four contralaterally innervated layers (2, 3, 4, 6) likely reflect the presence of two additional contralateral layers (3 and 6), a point which is reinforced by results of injections into individual layers, presented below.

Geniculate laminar analysis - In order to study the sizes of ganglion cells which project to individual laminae, 10 tree shrews received iontophoretic injections into single or multiple layers of the LGNd. The results of these experiments are best illustrated by five representative injections involving each of the six geniculate layers (Figs. 5 – 7). Due to the restricted size of the injections only small numbers of ganglion cells were labeled in each case, and thus, it was impossible to draw accurate conclusions concerning the percentages of ganglion cells projecting to different geniculate layers. However, analysis of ganglion cell sizes as well as some of their morphological characteristics clearly revealed differences between the cells projecting to the different geniculate layers or pairs of layers.

Injections restricted to, or principally involving layer 3 (see Fig. 1B and Fig. 5), label only small ganglion cells (1 – 34th percentile of the local population). Similarly, injections centered in layer 6

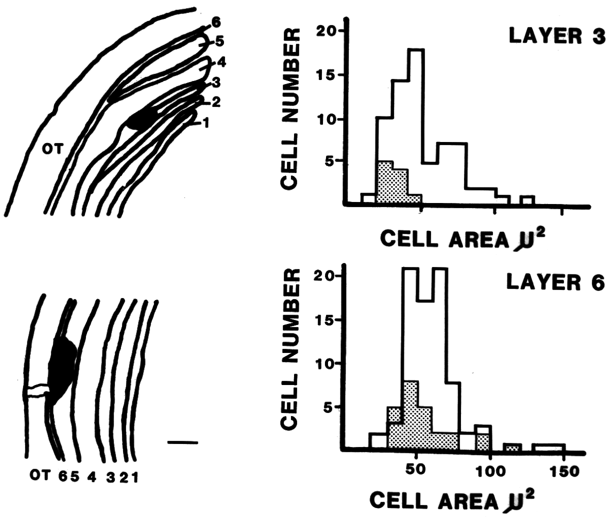

Figure 5. The results of two cases, one involving contralaterally innervated layer 3 and the other contralaterally innervated layer 6 and ipsilaterally innervated layer 5 of the LGNd. Histograms of labeled and unlabeled cells projecting to layers 3 and 6 are shown in the top and bottom histograms, respectively. In contrast to the other four LGNd layers, the majority of ganglion cells projecting to these two laminae are small in size, although some medium-sized and large cells project to layer 6 also. Scales = 500 *µm;* numerals refer to the layers of the LGNd; other conventions as in the previous figures.

involving also ipsilaterally innervated layer 5 (Fig. 5), label mainly small ganglion cells, although the size distribution of labeled cells is broader than that seen following layer 3 injections and includes some medium (35th – 74th percentile) and large (>74th percentile) ganglion cells (Fig. 5).

In contrast, injections restricted to the remaining four LGNd layers (1, 2, 4, and 5) result in labeled ganglion cells that are almost entirely medium and large in size relative to the local population (see Figs. 6 and 7). The ganglion cells projecting to these four layers can, in turn, be divided into two distinct populations based upon differences in the level of dendritic branching in some fortuitously well-filled cells. Of 26 cells (19 projecting to layers 4 and 5, 7 projecting to layers 1 and 2) in which the level of dendritic branching could be determined, <u>all</u> cells projecting to layers 4 and 5 branched high in the inner plexiform layer (IPL), near the inner nuclear layer, while those projecting to layers 1 and 2 branched lower within the IPL. It should, however, be stressed that these morphological distinctions be viewed with caution due to the incomplete filling in some of these cells, and due to the fact that dendritic branching depth is difficult to determine accurately in whole-mounted retinae. Nevertheless, the fact that dendrites on ON- and OFF-center ganglion cells in other species (e.g., Nelson et al., '78) terminate within separate tiers of the IPL, and that cells in LGNd layers 1 and 2 respond to the ON-set of light, while cells in layers

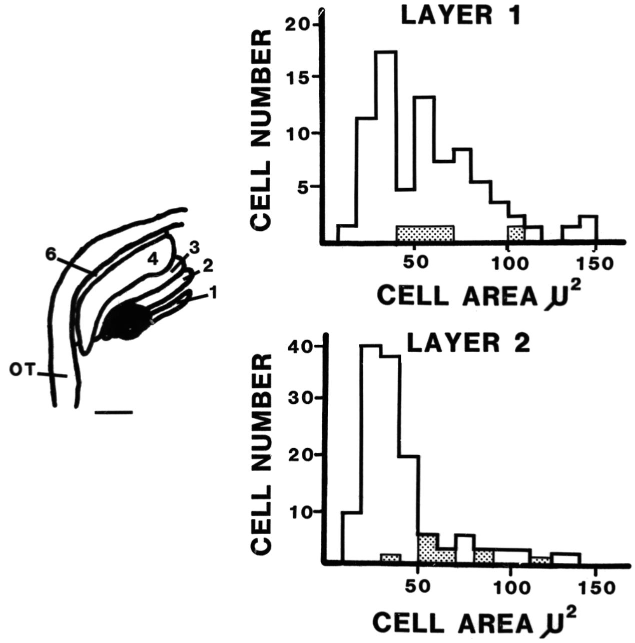

Figure 6: Results of a case in which the injection involved ipsilaterally innervated layer 1 and contralaterally innervated layer 2 of the LGNd. Large- and medium-sized ganglion cells are labeled. Scale= 500 *µm;* numerals refer to the layers of the LGNd; other conventions as in Figure 3.

4 and 5 respond predominantly to the OFF-set of light (present study and Conway and Schiller, '83), suggest that the morphological distinctions we have identified are valid. We will return to this point in the discussion.

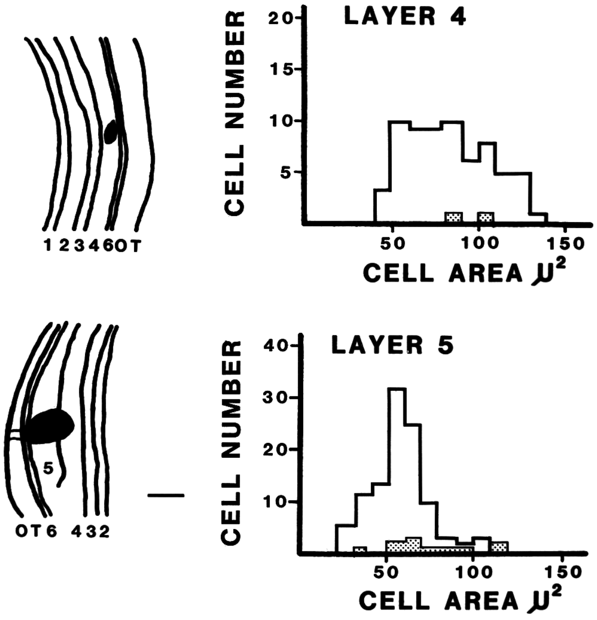

Figure 7: Results of two cases one involving contralaterally innervated layer 4 and one involving mainly ipsilaterally innervated layer 5 of the LGNd. As in Figure 6, only large- and medium-sized ganglion cells are labeled. Scales - 500 *µm.* Numerals refer to the layers of the LGNd; other conventions as in Figure 3.

The Superior Colliculus

The tree shrew superior colliculus is large, with well-developed layers. Retinal input projects to the superficial three layers terminating mainly within the thick grey layer, stratum griseum superficiale (SGS). The SGS can, in turn, be subdivided into at least two cytoarchitectonically distinct sublayers, the superficial (SGS_S) and deep (SGS_d) subdivisions (Graham and Casagrande, '80). A total of 16 separate injections (in 4 animals) were made into portions of the superficial layers of the colliculus in areas which represent both the monocular and binocular segments of the contralateral hemifield (Lane et al., '71). In none of these cases were we able to identify labeled ganglion cells in the ipsilateral retina, presumably due to the fact that this pathway is either very weak or absent in tree shrews (Conley et al., '85). In all 16 of the SGS cases injection sites included both subdivisions of the SGS.

Due to the consistent pattern of label in the contralateral retina in all of the above cases, the results of only three injections in one animal (78-33) are represented (see Fig. 8). In this case, injections into the middle or posterior portions of the colliculus which represent the physiologically defined

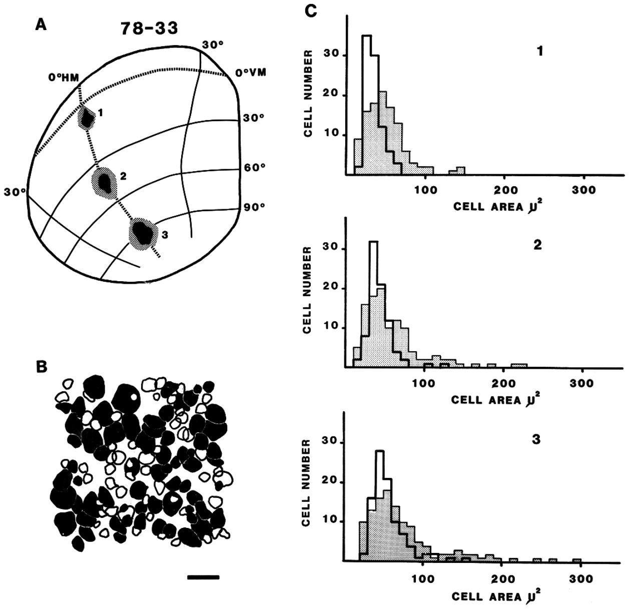

Figure 8: Results of three separate injections into the superior colliculus in one animal (78-33). All three injection sites were large enough to involve both the superficial and deep sublaminae of the superficial grey layer. A: A dorsal reconstruction of the injection sites. The map of the visual field is modified from Lane et al. (1971). B: The appearance of one 0.1 x 0.1 mm field from the retinal area containing the most densely labeled cells. Scale bar - 20 *μm.* C: Histograms of labeled vs. unlabeled cells illustrated separately for each injection site. The numbers of the sites correspond to the histograms in C. HM, horizontal meridian; VM, vertical meridian.

binocular and monocular segments, respectively, resulted in label in 56% of the cells within the densest patches of reaction product. The size distribution of labeled cells from all three injection sites appear similar to that seen in the contralateral retina following large LGNd injections with the exception that there is a greater tendency for small ganglion cells to project to the colliculus. As with extensive LGNd injections, virtually all of the largest ganglion cells are labeled after collicular injections involving both subdivisions of the SGS, suggesting that the largest ganglion cells have bifurcating axons that innervate both the LGNd and the colliculus. As with the LGNd there are also a few instances when injections produced cells whose dendrites were well filled, twenty-two such cells were drawn and were classified according to the morphological scheme developed in our previous communication (DeBruyn and Casagrande, '86b). Figure 9 shows the dendritic morphology of some of these cells and indicates their morphological type and subtype. If these cells are representative, then it appears that all morphological types (with the possible exception of one subclass, cluster 5) send axons to the superior colliculus.

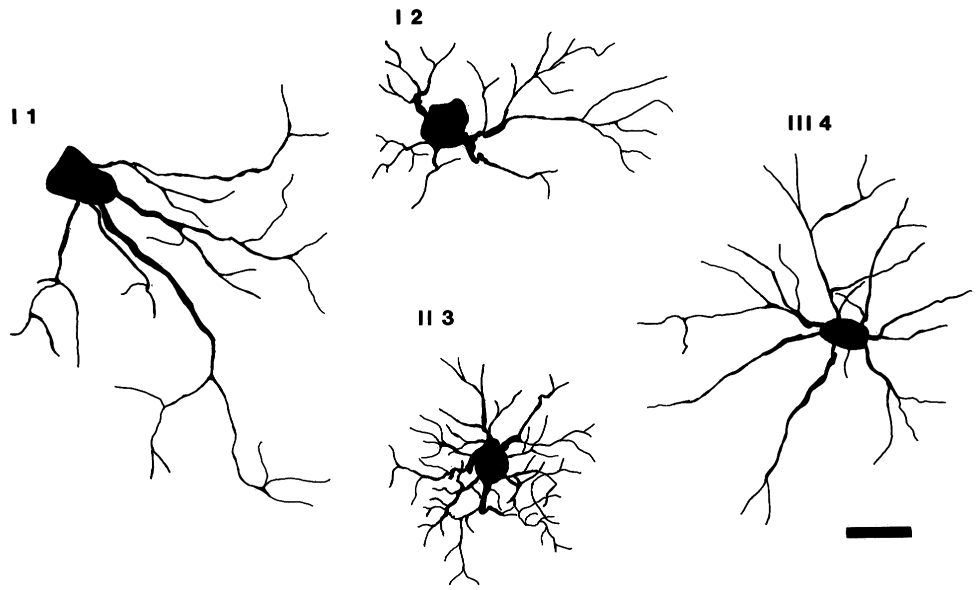

Figure 9: Camera lucida drawings of cell types backfilled following injections of the colliculus. The qualitative cell type and quantitative cluster number (DeBruyn and Casagrande, '86b) are indicated for each cell. DeBruyn and Casagrande ('86b) identified three major cell types, termed types I, II, and III which, in turn, could be subdivided into five clusters Note that four of the five cell clusters project to the colliculus. Scale *25µm.* See text for details.

In two additional animals, we made three injections restricted to the anterior pole of the colliculus which receives a separate contralateral projection from temporal retina (Lane et al., '71). One of these cases is illustrated in Fig. 10. In this case (80-15) two injections were placed in the rostral pole of the

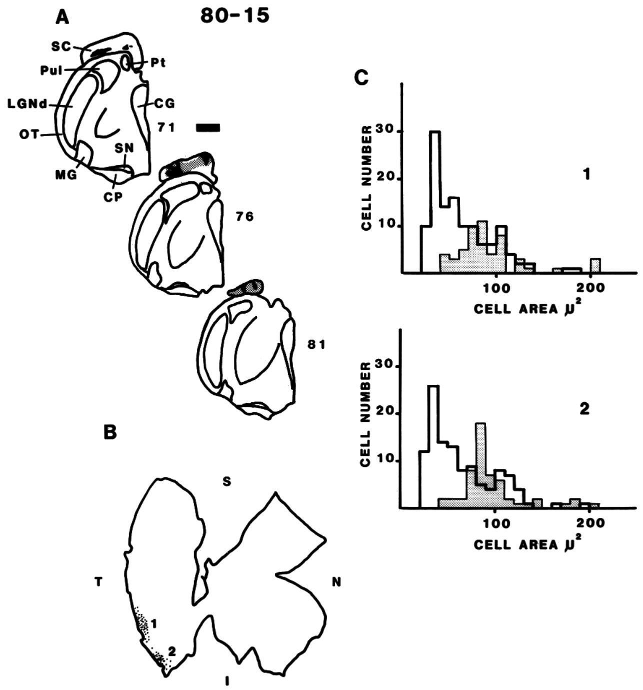

Figure 10: Results of two injections in one animal (80-15) into the rostral cap of the colliculus. A: The injection sites were confined entirely to the "cap region" of the colliculus, which represents the contralateral nasal visual field. B: The topographic distribution of labeled cells in the contralateral retina. No labeled cells were found in the ipsilateral retina. C: Histograms of labeled vs. unlabeled cells in this case. CG, central grey area; CP, cerebral peduncle; LGNd, dorsal lateral geniculate nucleus; MG medial geniculate nucleus; OT, optic tract; Pt, pretectum, pul, pulvinar nucleus; SC, superior colliculus; SN, substantia nigra; 1, 2 identify the locations of resulting labeled cells in retina and corresponding histograms. Scale = 1 mm, other conventions as in Figure 3.

colliculus. These injections resulted in two distinct patches of labeled ganglion cells in the inferior mporal portion of the retina (Fig. 10B). In contrast to the results of the cases described earlier, only 30% of the ganglion cells appear labeled. Moreover, the distribution of labeled cells was mainly medium in size (Fig. 10C). This result suggests that different topographic zones of the colliculus receive information from different subsets of retinal ganglion cells; the results presented below suggest that the same conclusion can be drawn for cells projecting to different sublayers of the colliculus.

Divisions of the superficial grey layer of the colliculus - In three animals restricted injections were made either within the SGS_s (4 injections) or SGS_d (4 injections) sublaminae of the stratum griseum superficiale. Examination of labeled cells within the retinae in these cases revealed that the percentages and sizes of ganglion cells which projected to the two sublaminae differ considerably. Following injections into the SGS_s approximately 41% of the cells appear labeled whereas an average of only 15%

of the cells appear labeled following an injection of comparable size into SGS_d. Figure 11 shows that although ganglion cells of all sizes project to the SGS_s, the majority are small to medium in size compared to the local population, whereas ganglion cells projecting to the SGS_d are composed almost

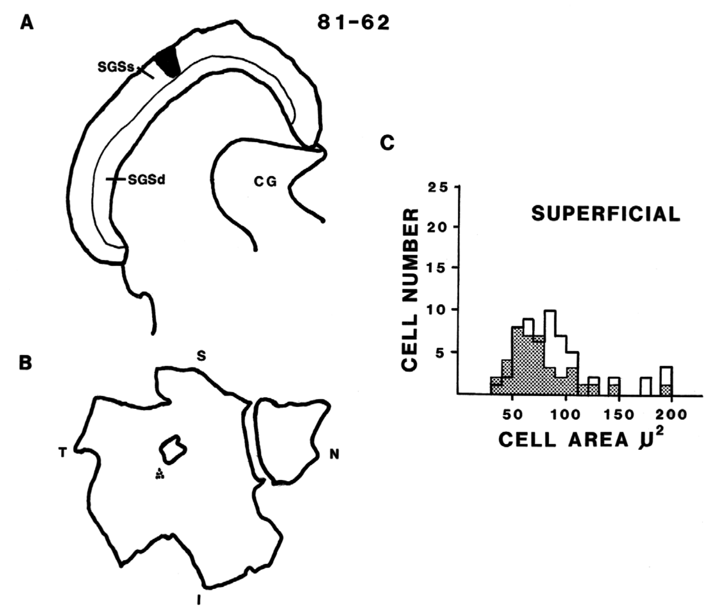

Figure 11: Results of case (81-62) in which the injection was confined to the superficial subdivision of the stratum griseum superficiale (SGS_s) of the superior colliculus. A: A drawing of the center of the injection site in one coronal section through the superior colliculus. B: The topographic distribution of labeled cells in the contralateral retina. C: Histograms of labeled vs. unlabeled cells. The average size of labeled cells is small, although some medium and large cells are also labeled. Compare with Figure 12. CG, central grey; SGS_d, deep subdivision of the stratum griseum superficiale; other conventions as in Figure 3.

exclusively of large ganglion cells (Fig. 12). Taken together, these results indicate that although there is overlap between the size and probably also the morphological characteristics of ganglion cells projecting to the major thalamic (LGNd) and midbrain (superior colliculus) targets of the retina, ganglion cells projecting to layers or sublayers within these targets form distinct populations.

The ventral lateral geniculate nucleus (LGNv)

In the tree shrew, the LGNv is a large, well-developed nucleus consisting of at least 3 (dorsal, medial, and lateral), subdivisions (Laemle, '68; Abplanalp, '71), only two of which, the dorsal and lateral divisions, receive retinal input (Laemle, '68). Two injections were made into the LGNv and as the results differed, (presumably due to differential involvement of the nuclear subdivisions) both will be described.

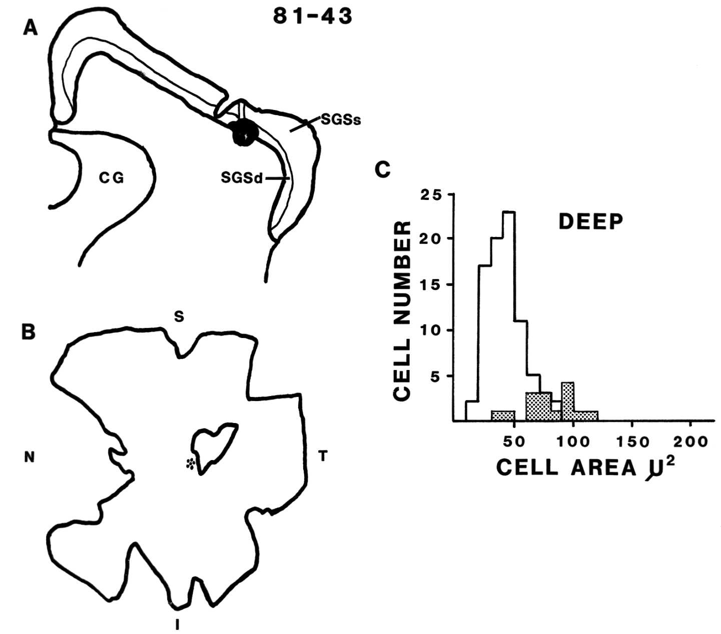

Figure 12: Results of case (81-43) in which injection was mainly confined to the deep subdivision of the stratum griseum superficiale (SGS$_d$). A: A drawing of the center of the injection site in one coronal section through the superior colliculus. B: The topographic distribution of labeled cells in the contralateral retina. C: Histograms of labeled vs. unlabeled cells. Note that the majority of labeled ganglion cells are large in size. Compare to Figure 10. CG, central grey area; SGS$_S$, superficial subdivision of the stratum griseum superficiale; other conventions as in Figure 3.

A single injection in the first case (77-24) involved the most lateral aspect of the nucleus as well as the optic tract (Fig. 13A). In addition to the dense patch of labeled cells in the inferior nasal quadrant of the retina, the injection produced labeled cells scattered throughout the remainder of the contralateral retina (Fig. 13B). The scattered cells, presumably labeled through involvement of the optic tract, will not be considered further. The labeled cells in the dense patch constituted 24% of the local population and were primarily small in size (Fig. 13C). Unfortunately, the ipsilateral retina was damaged during processing in this case and no information on cells projecting from this retina is available.

In a second case (83-1), the injection involved the dorsocaudal portion of the LGNv and included both retinal recipient subdivisions of the nucleus as well as the optic tract (Fig. 14A). Because WGA-HRP was used in this case, contamination of the results by optic tract involvement can be ruled out (see Methods). Resulting labeled cells appear in both retinae near the *area centralis* (Fig. 14B), the histograms of labeled vs. unlabeled cells in both retinae (Figs. 14C and 14D) reveal a difference between the ipsilaterally and contralaterally projecting cells. In the contralateral retina (Fig. 14C), labeled ganglion

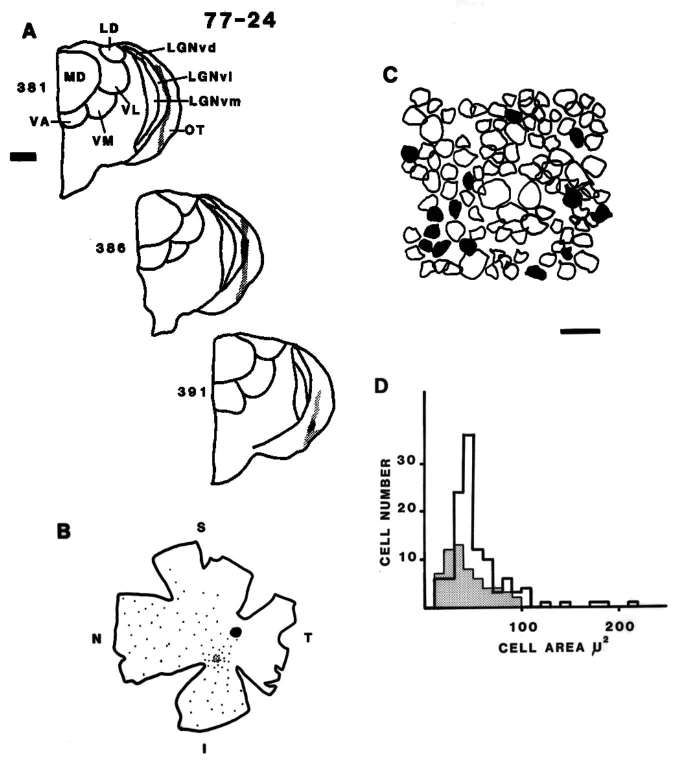

Figure 13: Results of a case (77-24) in which the injection involved the lateral division of the ventral lateral geniculate nucleus (LGNvl) A: the extent of the injection site shown at three coronal levels through the thalamus. Scale bar = 1mm. B: the topographic distribution of labeled cells in the contralateral retina. The scattered cells are presumably due to optic tract (OT) involvement. C: The appearance of one 0.1 X 0.1 mm field from the area of densest label. Scale bar = 20 μ. LGNvd, LGNvl, LGNvm, dorsal, lateral, and medial subdivisions of the ventral lateral geniculate nucleus; LD, lateral dorsal nucleus; MD, medial dorsal nucleus; VA, ventral anterior nucleus; VL, ventral lateral nucleus; VM, ventral medial nucleus; numerals indicate section levels; other conventions as in Figure 3.

cells are mainly large or small in size. Since no large, labeled cells are evident in the contralateral retina in case 77-24, it seems likely that the large, labeled cells in case 83-1 project to the dorsal subdivision of the nucleus, a region which was not involved in the previous injection. In the ipsilateral retina of case 83-1 only large ganglion cells appear labeled (Fig. 14D). Together, the results of both cases indicate that there may be an exclusive subdivision or "layer" within the lateral subdivision of the LGNv that receives input only from small ganglion cells within the contralateral retina. This arrangement resembles that seen in the LGNd where two layers (3 and 6) also appear to receive an exclusive input from small, contralaterally-projecting retinal ganglion cells.

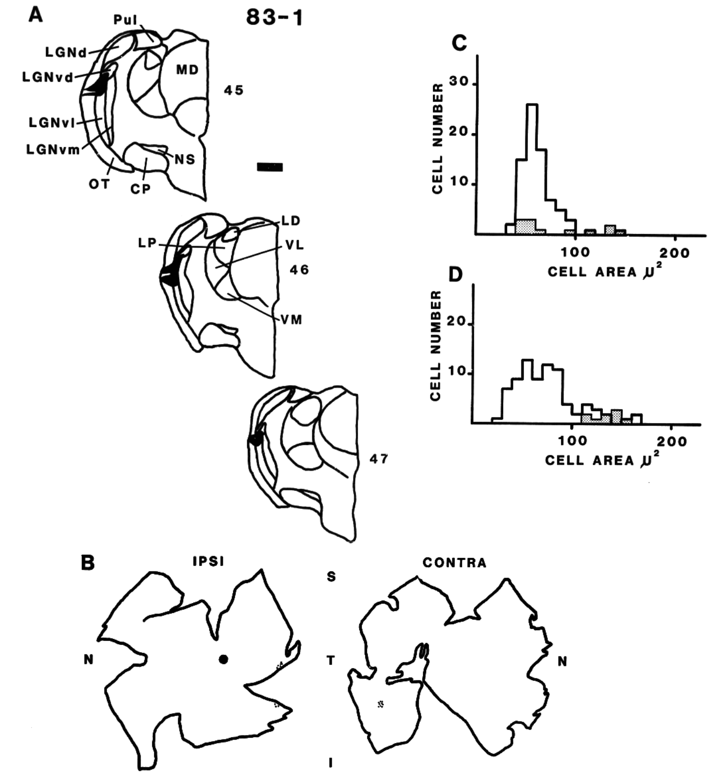

Figure 14: Results of a case (83-1) in which the injection involved all three divisions of the ventral lateral geniculate nucleus (LGNv). A: The extent of the injection site shown at three levels through the thalamus. Scale bar = 1 mm. B: The topographic distribution of labeled cells in the contralateral (contra) and ipsilateral (ipsi) retinae. C: Histograms of labeled vs. unlabeled cells in the contralateral retina. The majority of labeled cells are small and large in size. D: Histograms of labeled vs. unlabeled cells in the ipsilateral retina. Only large cells are labeled. GP, cerebral peduncle; LD, lateral dorsal nucleus; LGNd, dorsal lateral geniculate nucleus; LP, lateral posterior nucleus; MD, medial dorsal nucleus; OT, optic tract; pul, pulvinar nucleus; NS, subthalamic nucleus; VL, ventral lateral nucleus; VA, ventral anterior nucleus; numerals refer to section levels; other conventions as in Figure 3.

The Pretectum

The pretectal complex in the tree shrew consists of five separate nuclei (the anterior, posterior, and medial pretectal nuclei, the nucleus of the optic tract and the pretectal olivary nucleus). Of these nuclei, two (the nucleus of the optic tract and the olivary nucleus) receive the majority of retinal projections; all except the nucleus of the optic tract receive bilateral input (Weber and Harting, '80). The present results are based on two injections in two animals (592 and 82-35).

The first case is illustrated in Figure 15. The injection covered parts of 4 nuclei (anterior and posterior nuclei, olivary nucleus and nucleus of the optic tract) but mainly affected the posterior nucleus and nucleus of the optic tract (Fig. 15A) and resulted in labelling 20% of the ganglion cells in both retinae

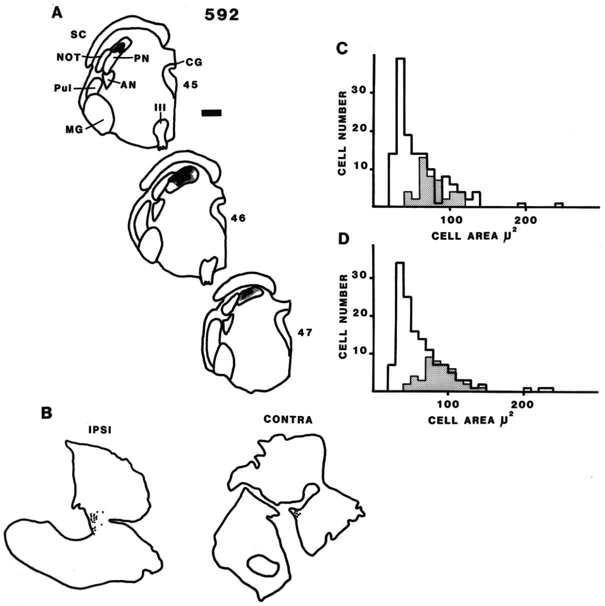

Figure 15: Results of an injection involving four subdivisions of the pretectum. A: The injection site depicted at three levels through the midbrain primarily involved the posterior nucleus and nucleus of the optic tract, although some involvement of the anterior and olivary nuclei was also evident. Scale bar = 1 mm. B: the topographic distribution of labeled cells in the contralateral (contra) and ipsilateral (ipsi) retinae. Because of tissue mounting difficulties in this case, we were uncertain of the orientation of the retinae and thus, no conclusions about the relative eccentricity of the labeled patches could be drawn. C and D: Histograms of labeled vs. unlabeled cells in the contralateral and ipsilateral retinae, respectively. In both cases, approximately 20% of the cells are labeled, with the labeled cells being predominantly medium and large in size. AN, PN, ON, anterior, posterior and olivary pretectal nuclei; NOT, nucleus of the optic tract; CG, central grey; MG, medial geniculate nucleus; SC, superior colliculus; pul, pulvinar; III, third nerve; numerals indicate section levels; other conventions as in Figure 3.

(Fig. 15B). Due to problems in orienting and mounting the retinae, it was impossible to determine the exact location of the labeled cells in these retinae. Frequency histograms (Fig. 15C) show that the majority of labelled cells from both retinae were medium to large in size.

In the second case (82-35), the injection was restricted mainly to the nucleus of the optic tract (Fig. 16A) and labeled cells were found only in the inferior contralateral retinal (Fig. 16B). As in the previous case, the labeled cells were mainly medium to large in size. Because the results of the case were similar to those obtained in the ipsilateral retina of case 592, it seems evident that the sizes of retinal ganglion cells projecting to the nucleus of the optic tract (which has no ipsilateral input) are similar to those projecting to other subdivisions of the pretectum.

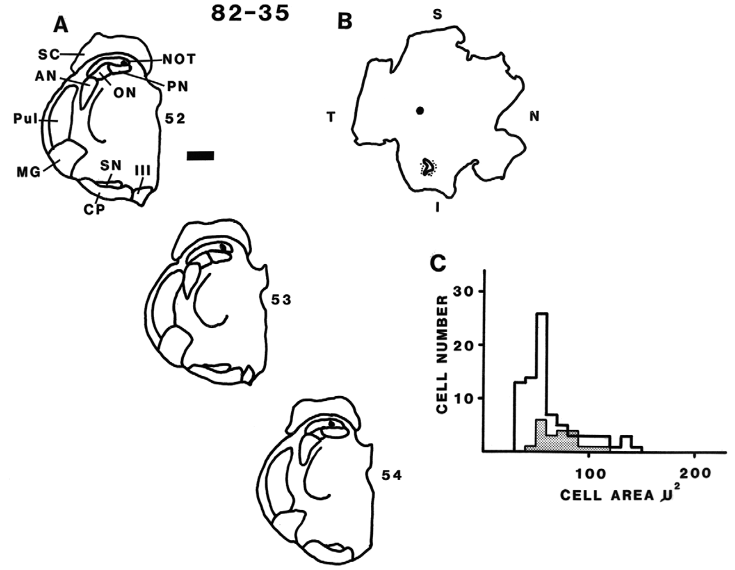

Figure 16: Results of a pretectal injection involving mainly the nucleus of the optic tract (NOT). A: The injection site is depicted at three levels through the midbrain. Scale bar = 1 mm. B: the topographic distribution of labeled vs. unlabeled cells in the contralateral retina. C: Histograms of labeled vs. unlabeled cells in the contralateral retina. The labeled cells are medium to large in size. Compare with the results in the previous figure. SN, substantia nigra; other conventions as in Figure 15.

The medial and lateral terminal nuclei of the accessory optic system

All three nuclei of the accessory optic system, the medial (MTN), lateral (LTN) and dorsal (DTN) terminal nuclei have been identified cytoarchitectonically in tree shrews and all three receive a crossed retinal input. Of these nuclei, MTN is the largest and receives more than 90% of the input from the accessory optic tract (Tigges, '66). Two injections were made into the nuclei of the accessory optic system. The first involved only the MTN while the second involved both MTN and LTN.

In the first case (618), the center of the injection was located within the inferolateral portion of MTN (Fig. 17A) and resulted in label in a group of very large (>90th percentile), ganglion cells in the contralateral retina (Fig. 18B). Examination of these cells indicate that they are probably part of a morphologically separate population which may not have been labeled in our previous study characterizing ganglion cell types in tree shrews (DeBruyn and Casagrande, '86b). The somas of these cells appear to lie in the deepest part of the ganglion cell layer, and each is characterized, in addition to its large cell body size, by three stout primary dendrites and the tendency to group in pairs of labeled cells.

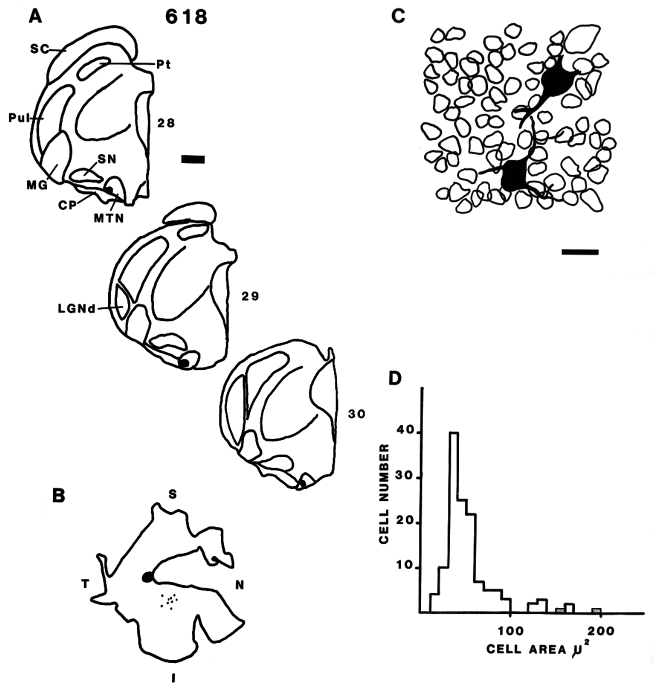

Figure 17: Results of an injection into the medial terminal nucleus (MTN) of the accessory optic system. A: The injection site depicted in three levels through the thalamus. The injection mainly involved the inferolateral portion of the nucleus. Scale bar = 1mm; B: The topographic distribution of labeled cells in the retina. C: The appearance of one 0.1 x 0.1 mm field from the area of densest label. Note that very few labeled cells are contained within this area. Scale bar = 20 μ. D: Histogram of labeled vs. unlabeled cells in the contralateral retina within a 0.1 x 0.1 mm square area of densest label. GP, cerebral peduncle; LGNd, dorsal lateral geniculate nucleus; MG, medial geniculate nucleus; Pt, pretectum; SC superior colliculus; SN substantia nigra; pul, pulvinar, numerals indicate section levels; other conventions as in Figure 3.

In a second case, a large injection was made which involved both MTN and LTN (Fig. 18A). Since the MTN receives the vast majority of retinal input, it is likely that results of this case reflect mainly cells projecting to the MTN. The results reveal a rather remarkable topographic distribution of the cells. Figure 18 shows that the labeled cells occur in widely scattered pairs (see Fig. 18C) with an overall density of approximately 25 cells/mm^2. When a nearest neighbor analysis (Wässle et al., '81) was performed on these ganglion cells, it was found that the average distance between cells within a pair was less than 50 *μm,* while the average distance to the next 6 nearest cells was over 350 *μm.* Moreover, the cells were found to be distributed in a roughly hexagonal matrix. In addition to the paired cells, occasional unpaired large cells were evident, however, their density was considerably lower (1 – 2 cells/mm^2) than that of the paired cells. As with the first case, the cells in this case were very large, had three thick primary dendrites, and tended to lie deep in the ganglion cell layer. However, none of the

labeled cells appeared to be displaced as has been reported for cells projecting to the avian homolog of the MTN (Karten et al., '77; Brecha and Karten, '79).

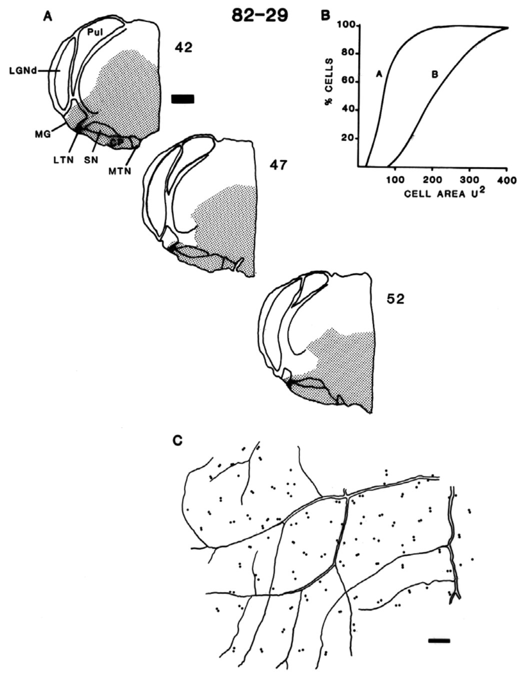

Figure 18: Results of an injection involving both the medial and lateral terminal nuclei of the accessory optic system. A: The injection site depicted at three levels through the thalamus. Scale = 1 mm. B: Cumulative frequency graph illustrating the size difference between (A) 100 cells illustrating the local size population and, (B) 64 cells projecting to the accessory optic system (AOS). Note that all cells projecting to the AOS are larger than 75% of cells in the local population, and that the median size of cells projecting to the AOS is in the top 5% of local cell sizes. C. Camera lucida drawing of the distribution of labeled cells in the retina. The vast majority of cells occur in well-defined pairs, with the average within pair distance being less than 50 μm and the average between pair distance being over 350 μm. Scale = 300 μm. CP, cerebral peduncle; LGNd, dorsal lateral geniculate nucleus; LTN, lateral terminal nucleus; MG, medial geniculate nucleus; MTN, medial terminal nucleus; Pul, pulvinar nucleus; SN, substantia nigra.

DISCUSSION

A main goal of the present study was to establish whether different classes of retinal ganglion cells in tree shrews project to different central targets or subdivisions of these targets. The results clearly provide evidence for differences in the distribution of size classes projecting to different targets. Since ganglion cell size correlates roughly with morphological cell class in tree shrews (DeBruyn and Casagrande, 1986b), we can also conclude that the results provide evidence for differences in the distribution of morphological ganglion cell classes projecting to different targets, a point which is reinforced in some instances by observed differences in dendritic morphology. However, the results also support the view that most retinal targets do not receive exclusive input from a distinct class of ganglion cells; rather, the relative proportion of inputs from different classes varies between targets or target subdivisions. Moreover, the results suggest that the relative proportion of input from different classes of ganglion cells to a particular central target varies according to regional retinal specialization, e.g., differences are obvious in the comparison of ganglion cells projecting from corresponding points in nasal versus temporal retina. In the discussion that follows we consider what these findings imply about the functional significance of ganglion cells, and how the results compare with what is known about the functional organization of these central retinal recipient zones in tree shrews and other species.

The Dorsal Lateral Geniculate Nucleus

As described earlier, the cellular morphology, physiology, and connections of the LGNd of tree shrews have been studied in detail. For the purposes of this discussion the most interesting aspects of LGNd organization concern: 1) the degree to which layers or pairs of layers, which differ in their characteristics, are innervated by unique populations of retinal ganglion cells, and 2) the degree to which separate LGNd layers preserve and transmit information inherited from the retina.

Regarding the question of layer innervation by unique ganglion cell populations, our data show that layers 3 and 6 are clearly different, being primarily innervated by small ganglion cells. With layers 1, 2, 4 and 5, however, establishing whether each is innervated by unique ganglion cell populations involves more than an appreciation of cell size differences since both sets of layers are innervated by large- and

medium-size ganglion cell populations. However, as our results show, retinal ganglion cells projecting to layers 1 and 2 can be distinguished from those projecting to 4 and 5 based upon the level at which their dendrites branch within the inner plexiform layer (IPL) of the retina; ganglion cells projecting to layers 1 and 2 branch closer to the ganglion cell layer (sublamina b of the IPL) than to those projecting to layers 4 and 5 (sublamina a of the IPL). Presumably these differences in dendritic stratification reflect differences between ON- and OFF-center ganglion cells that innervate LGNd layer pairs 1, 2 and 4, 5 which contain ON- and OFF-center cells, respectively. Findings in other species have also provided evidence that ON- and OFF-center ganglion cells can be distinguished based upon a dichotomy in dendritic branching within the IPL (e.g., Nelson et al., '78), and that segregation of ON-center and OFF-center retinal projections is, in some instances (e.g., mink and ferret), preserved at the level of the LGNd (LeVay and McConnell, '82; Stryker and Zahs, '83).

These data indicate that the visual information abstracted by different classes of retinal ganglion cells is to a great degree preserved by being sent either to separate cell types (e.g., X and Y cells) or to separate layer pairs (ON-center and OFF-center channels) within the tree shrew LGNd. It is noteworthy that the separation of the ON- and OFF-center channels in tree shrews is preserved anatomically and physiologically beyond the LGNd. LGNd layers 1 and 2, containing primarily ON-center cells, project to the upper tier of layer IV of striate cortex, while layers 4 and 5, containing primarily OFF-center cells, project to the lower tier of layer IV of striate cortex (Harting et al., '73; Conley et al., '84). Cells in the upper and lower tiers of striate cortex, in turn, reflect their major laminar LGNd inputs and exhibit mainly ON- or OFF-center activity, respectively (Norton et al., '85). At present, it does not appear that other properties, such as those defining X and Y cells in the LGNd are preserved at the cortical level in tree shrews to the same degree as ON- and OFF-center information. ON- and OFF-center distinctions are also lost beyond layer IV of striate cortex, although, at least one anatomical study has suggested that spiny stellate cells within the upper and lower tiers of cortical layer IV provide input to different subtiers of cortical layer III, indicating that some aspects of the original channel separation may be maintained (Humphrey and Lund, '79).

In other species, evidence suggests that information from different LGNd layers can be preserved to a degree in cortex. For example, in the mink, separation of ON- and OFF-center retinal information is preserved within layer IV of striate cortex where it appears to be organized into patches (McConnell and LeVay, '83). The usefulness of keeping ON- and OFF-center channels separate at the cortical level is not immediately obvious although recent evidence in monkeys suggests that separation of ON- and OFF-center information may be important for enhancing contrast and thus increasing the visibility of forms against a background (Schiller, '84).

Unlike layers 1, 2, 4 and 5, contralaterally innervated layers 3 and 6 receive an input from the smallest ganglion cells. Moreover, some ganglion cells projecting to layer 6 can also be distinguished from those projecting to layer 3 based on size since a few cells projecting to layer 6 are also medium to large in size. From our previous analysis of the sizes of morphologically distinct classes of tree shrew retinal ganglion cells, we would argue that a high proportion of the small cells projecting to layers 3 and 6 must belong to one class of cells, namely, Type III cells (DeBruyn and Casagrande, '86b). These cells resemble the small gamma cells (Boycott and Wässle, '74) or W-cells seen in the cat retina, having large dendritic fields and fine axons. These findings are in accord with earlier studies as well as other aspects of the present study suggesting that cells with W-like properties and/or conduction velocities are present in layers 3 and 6 of the LGNd which contain the smallest cells (Casagrande et al., '78; Conway and Schiller, '83; Norton, unpublished).

From a functional standpoint, several points are noteworthy about the retinal input to, and the organization of, LGNd layers 3 and 6. First, ganglion cells innervating these layers have no ipsilateral equivalent and may account for the greater proportion of labeled small ganglion cells seen in the nasal retina. This arrangement also suggests that the role of layers 3 and 6 has less to do with binocular integration than is the case for the remaining layers. Second, the SGS$_S$ of the superior colliculus also receives input from small retinal ganglion cells (perhaps the same ones) and sends projections to layers 3 and 6 (Fitzpatrick et al., '80). This projection pattern indicates that layers 3 and 6 are intimately connected functionally with the superior colliculus. Third, LGNd layers 3 and 6 both project to sublaminae within supragranular striate cortex; layer 3 projects to cortical layers IIIb and I and LGNd layer 6 to cortical

layer IIIc (Conley et al., '84). The fact that cells in LGNd layers 3 and 6 receive and send information mainly via fine axons that terminate in these cortical layers suggests that the cells in these pathways are not part of a major excitatory channel to cortex, but instead either modulate activity or provide more specific information to higher order neurons, perhaps concerning the activity of the superior colliculus. Finally, similarities in ganglion cell morphology, physiology, and central projections of small LGN cells in several species including cats and primates (galago) suggest that this arrangement is not unique to tree shrews but instead represents a more universal mammalian feature (Wilson and Stone, '75; Fitzpatrick et al., '80; 83; Torrealba et al., '81; Casagrande and DeBruyn, '82; Itoh et al., '82; Livingstone and Hubel, '84; Huerta et al., '85; Kaas, '85; Irvin et al., '86).

The Superior Colliculus

Results of large injections involving all retinal recipient subdivisions of the superior colliculus clearly demonstrate that all cell size classes project to the colliculus. In fact, our data suggest that all morphological ganglion cell classes, with perhaps one exception are represented in this projection. In this sense, the retinocollicular pathway in tree shrews is similar to that of other lateral-eyed mammals such as rodents and lagomorphs where all classes of ganglion cells appear to project to the colliculus, and is different from that of mammals with wide binocular overlap such as cats and primates where the pathway consists either of projections from large (alpha) or small (gamma) ganglion cells as in cats, or mainly small (gamma-like) ganglion cells as in primates (Bunt et al., '75; Kelly and Gilbert, '75; Dreher et al., '76; Kelly and Fox, '77; Rhoades and Chalupa, '78; Molotchnikoff et al., '79; Itoh et al., '81).

However, the above conclusions ignore the most important observations, namely, that, like the LGNd, the colliculus cannot be viewed as a single functional unit. Rather, it contains distinguishable subdivisions, sublayers and/or topographic regions.

The major retinal recipient subdivisions of the tree shrew colliculus are the SGS_S and SGS_d, and the anterior cap region, which our unpublished observations suggest is a specialized extension of the SGS_S. Analysis of cell sizes and the morphological classes of ganglion cells projecting to these subdivisions indicate that they may be part of separate systems that function in parallel. The cells of the

SGS$_S$ are primarily stationary responsive (S-R) cells with ON-OFF receptive field centers and suppressive surrounds (Albano et al., '78). Since the receptive field properties of S-R cells closely resemble those described for one class of retinal ganglion cell (ON/OFF cells), the majority of ganglion cells projecting to the SGS$_S$ probably belong to this physiological class. We have not been able to positively correlate ON/OFF ganglion cells with their morphological counterparts. However, since morphological Type I and type II cells do correlate with other physiological cell types, some of the morphological Type III cells with large dendritic fields (matching the large receptive fields of the S-R cells) likely represent the major input to the SGS$_S$ (DeBruyn and Casagrande, '86b).

The organization and connections of the deep substratum of the SGS (SGS$_d$) are different in a number of respects from that described above. In contrast to the SGS$_S$, the SGS$_d$ receives input primarily from large ganglion cells, and is composed of a several physiological and morphological cell types which, in turn, project to the pulvinar nucleus of the thalamus (Albano et al, '79; Graham and Casagrande, '80). Moreover, correlates between the anatomy and physiology of retinal ganglion cells projecting to the SGS$_d$ and the properties of the cells within this substratum are less obvious than for the SGS$_S$, since SGS$_d$ cells are heterogeneous, and most do not have obvious retinal counterparts (Albano et al., '78). This could result from the fact that many SGS$_d$ cells send dendrites to the surface of the colliculus, and are, therefore, in a position to integrate information from retinal ganglion cells projecting to both substrata of the SGS (Irvin et al., '83). Further, since conduction latencies to the SGS$_d$ cells from chiasm stimulation are on average, longer (Norton, unpublished) than those to SGS$_S$ cells, it seems likely that the influence on the SGS$_d$ cells of many of the larger ganglion cells (presumably with faster conducting axons) is indirect. Ultimately, the influence of the SGS$_d$ on visual behavior could be quite complex since cells in SGS$_d$ are physiologically heterogeneous and together could influence, via pulvinar, wide regions of extrastriate cortex which is known to contain several visual areas (Sesma et al., '85).

How unique is the arrangement of differential retinal inputs to the colliculus that we have revealed in tree shrews? In mice, results very similar to ours have been reported, namely, that the SGS$_S$ and SGS$_d$ receive projections from distinct populations of smaller and larger retinal ganglion cells

(Hofbauer and Dräger, '85). In rats and hamsters, a similar pattern is seen, but investigators have argued that there may be three subpopulations of ganglion cells projecting to subdivisions of the SGS (Chalupa and Thompson, '80). In cats, evidence suggests that cells projecting to the upper and lower subtiers of the SGS are distinct morphologically and physiologically; W (gamma) cells and Y (alpha) cells project to the SGS_s and SGS_d, respectively (Itoh et al., '81). Since correlations between cell size and physiology are clearly documented only in cats, it is not evident if the physiology of the retinocollicular pathways in tree shrews and other species is similar to that of the cat. Evidence against a strict translation from cats to other species such as tree shrews and opossums is that large collicular injections in the latter species label ganglion cells of all sizes and all major morphological classes, not just those with alpha- and gamma-like morphology (present study and Rapaport and Wilson, '83). Also, in tree shrews the size distribution of cells projecting to the SGS_s and SGS_d is not as restricted as reported for cats indicating that some ganglion cells of all sizes project to both subdivisions; the difference in the projection pattern lies in the *relative* distribution of ganglion cell sizes projecting to the two subdivisions.

Ganglion Cells Projecting to Other Retinal Targets

Our major effort in this study was aimed at determining, in detail, the distribution and sizes of ganglion cells projecting to the LGNd and superior colliculus. In addition, we examined ganglion cells projecting to four other retinal targets: the ventral lateral geniculate nucleus (LGNv), the pretectum (Pt), and two nuclei of the accessory optic system, the medial and lateral terminal nuclei, MTN and LTN.

The reason we have grouped comments concerning these four areas together is not because we feel they are functionally related, but rather to emphasize the limited nature of our observations. Undoubtedly a more detailed analysis of ganglion cells projecting to the many subdivisions of some of these areas will ultimately be necessary for us to fully appreciate their complexity. Regardless, our data reveal interesting general features of the retinal ganglion cells projecting to each of these areas, features which are worth discussing in light of the possible functional significance of each of these target areas.

Our data concerning projections to LGNv are based on two cases, which have rather different results. Since the injections involved different subdivisions of LGNv in the two cases, it is reasonable to

argue that the differences we observed reflect differences in ganglion cell classes projecting to each subdivision. The dorsal subdivision receives projections from large ganglion cells in both eyes, the lateral subdivision from small ganglion cells in the contralateral retina. Although a physiological study of the LGNv in tree shrews did not address the issue of functional subdivisions within the nucleus, the data concerning conduction latencies of axons projecting to cells in LGNv are in accord with our results, demonstrating that both fast and slowly conducting fibers innervate the LGNv (Kuyk et al., '82). These data contrast with those found in cats where only slowly conducting (presumed W-cells) from the contralateral eye were found to project to the LGNv (Spear et al., '77). Nevertheless, in both tree shrews and cats LGNv cells have large receptive fields and heterogeneous response properties that are quite distinct from the majority found in the LGNd. Functionally, relatively little is known about the LGNv although it has been linked to systems concerned with pupillary light reflexes, eye movements and detection of brightness (e.g., Polyak, '57). Since our data provide evidence that distinct populations of retinal ganglion cells project to different subdivisions of the LGNv, it is not impossible to imagine that through its subdivisions the LGNv is specialized to perform more than one of the above functions.

The pretectum of the tree shrew is more complex in terms of subdivisions, than the LGNv, consisting of five nuclei that are likely to be functionally distinct based upon differences in central connections (Weber and Harting, '80). As with the LGNv, our analysis of ganglion cells projecting to pretectum is based upon two cases, one of which clearly involved the nucleus of the optic tract (NOT) and the other of which involved four separate pretectal nuclei but mainly NOT and the posterior pretectal nucleus (PN). Since both cases yielded nearly identical size distributions of labeled cells (mainly medium and large), one could argue that both cases actually reflect input only from the NOT. However, since the large pretectal injection case yielded nearly equal numbers of labeled cells in both the ipsilateral retina and the contralateral retina, the result must reflect ganglion cells projecting to a nucleus that receives bilateral input, the most likely candidates being (PN) or the olivary nucleus (ON), but not NOT which receives only a contralateral retinal input. It is also unlikely that the ipsilateral input reflects interruption of fibers of passage to the colliculus since in most tree shrews the colliculus only receives contralateral retinal input (Conley et al., '85). The similarity in the size distribution of ganglion cells projecting to more

than one pretectal area is surprising and contrasts with our findings for other retinal targets with subdivisions or layers. However, our data cannot rule out the possibility that NOT, PN, and ON receive distinct projections since there is considerable overlap in the size of morphologically distinct ganglion cell classes in tree shrews. We can, however, conclude that the proportion of small ganglion cells projecting to these zones of the pretectum is small. This finding can be contrasted with recent data in the cat which suggest that 47% of the cells projecting to the pretectum are small in size (Koontz et al., '85). In addition, these authors also report pretectal projections from large (alpha) cells and medium-size (beta and epsilon) cells. Since the NOT in cat does not receive a direct projection from physiological Y-cells, the functional counterpart of alpha cells, they argue that these large cells must terminate outside of NOT. In tree shrew NOT clearly receives input from the largest cells which are made up of two morphological classes, only one (cluster 2) of which is likely to be the morphological counterpart of physiologically identified Y-cells (Van Dongen et al., '76; DeBruyn and Casagrande, '86b). We have argued previously that the other morphological subclass of large ganglion cells (cluster 1) is likely to be the counterpart of the physiologically identified directionally-selective cell. If this true, then NOT in the tree shrew may be functionally similar to NOT in the cat which contains directionally-selective cells (Hoffman and Schoppman, '75, '81). It is less easy to posit a guess as to the role of the medium-size ganglion cells, although it is likely, given the high percentage of medium-size ganglion cells projecting the pretectum that at least a small percentage of these are morphologically beta-like (Type II or cluster 3 in our terminology) and represent, as in cat (Fukuda and Stone, '74), evidence for physiological X-cell input to the pretectum (see also DeBruyn and Casagrande, '86b).

The present finding of an exclusively crossed projection from large ganglion cells to the MTN and LTN is in good agreement with those of other investigators which describe similar projections in a number of avian (Karten et al., '77; Brecha and Karten, '79; Reiner et al., '79), reptilian (Reiner and Karten, '78; Reiner, '81), fish (Finger and Karten, '78) anuran (Montgomery et al., '79) and mammalian (Kimm et al., '79; Oyster et al., '80) species. An exception to this is the cat, in which Farmer and Rodieck ('82) reported that small ganglion cells project to the accessory optic system. In non-mammalian species, this projection seems to be composed exclusively of displaced ganglion cells (e.g., Karten et al., '77;

Finger and Karten, '78; Reiner and Karten, '78); however, with the exception of the chinchilla in which at least some cells are displaced (Kimm et al., '79), this feature is not characteristic of mammals (Oyster et al., '80; Farmer and Rodieck, '82; present results).

The most noteworthy feature of ganglion cells projecting to the accessory optic system in tree shrews is their arrangement. They occur in a regular arrangement of widely separated pairs. Such a pattern has not been reported for other species (Karten et al., '77; Oyster et al., '80; see also Simpson, '84 for review). A clue to the functional significance of such pairing is suggested from physiological work in rabbits. In rabbits as in other species, cells in the accessory optic nuclei are directionally selective. Simpson ('84) has argued that the preferred excitatory and inhibitory directions of LTN and MTN neurons are transmitted from retinal ganglion cells. It is tempting to speculate that each pair of ganglion cells consists of one excitatory and one inhibitory cell, and that each pair provides directional information for a limited area of retina. This arrangement is analogous to that reported for ON- and OFF-center alpha and beta cells in the cat (Wässle et al., '81; Peichl and Wässle, '81). One difficulty with this argument is that no such paired arrangements of ganglion cells projecting to the accessory optic nuclei have been identified in rabbits (Oyster et al., '80). Perhaps, the rather precise projection patterns to the LGN and MTN in tree shrews reflect unique vestibular-ocular reflex requirements of these agile, squirrel-like, arboreal mammals.

How Exclusive are Retinal Channels to Central Targets?

In recent years, it has become popular to emphasize the degree to which different ganglion cells classes give rise to exclusive pathways that project in parallel to different subcortical visual centers and their subdivisions, much as we have done in the foregoing discussion. Evidence in tree shrews and other species certainly suggests many instances in which classes of retinal ganglion cells, such as ON- and OFF-center, or X and Y cells, project precisely to their counterparts in specific retinal targets (see Rodieck and Brening, '83 for review). However, it is also revealing to consider the degree to which different retinal targets must share information processed by the same ganglion cells. In tree shrews, it is clear that the major ganglion cell size groups that correlate with the three major morphological classes

(DeBruyn and Casagrande, 86b), are represented in projections to almost all of the main retinal targets and, in many instances, to their subdivisions as well. For example, both the LGNd and superior colliculus receive input from the full range of ganglion cell sizes. Even subdivisions of these areas, such as the SGS_S and LGNd layer 6, which receive mainly from small ganglion cells, also receive input from medium and large ganglion cells. One could, of course, argue that since there are actually five morphological classes of ganglion cells with considerable size overlap and as many as eight physiological types in tree shrew retina (Van Dongen et al., '76), projections to each of the subdivisions may be entirely unique. However, inspection of the percentages of labeled cells within each size group argues against this point. Figures 4 and 9 involving large injections into the LGNd and colliculus, respectively, clearly show that all of the largest ganglion cells project to these two structures and therefore must send axons simultaneously to at least three, and, likely more, retinal targets since other targets also receive projections from cells in this size range. The literature suggests that the same is true of other non-primate mammalian species (Wässle and Illing, '81; Illing and Wässle, '81; Rapaport and Wilson, '83). One can also argue that at least a percentage of medium-size ganglion cells must send axon collaterals to more than one retinal target. Large injections into either the LGNd or colliculus label more than 50% of cells within the medium-size range (LGNd = 73%: colliculus = 67%), indicating at least some medium-size ganglion cell axons branch to more than one central structure. It is more difficult to ascertain if small ganglion cells innervate more than one central target since the percentages of small, labeled cells projecting to any one retinal target is always less than 50%. However, since double labelling experiments have demonstrated that all major classes of cat ganglion cells have bifurcating axons (Illing, '80), it seems reasonable to assume that at least some small tree shrew retinal ganglion cells possess them also.

In functional terms, widespread axon collateralization obviously suggests that the visual information extracted by individual ganglion cells is useful in several contexts, (i.e., important to the roles of a number of subcortical visual centers that likely perform rather different functions). In tree shrews the strongest evidence for single ganglion cells innervating multiple central targets is for the large cells. Our previous results of tree shrew retinal ganglion cells suggest that most of the large ganglion cells are transient or Y-like cells (DeBruyn and Casagrande, '86b). One advantage to distributing information

broadly via a rapidly conducting pathway capable of signaling change would be as an alerting mechanism, readying each recipient zone to receive more specific incoming visual information from other ganglion cell classes. This arrangement has been suggested for cat Y cells (Spitzer and Hochstein, '85), and could be particularly advantageous to a rapidly moving arboreal mammal requiring quick decisions concerning its visual environment.

It is more difficult to speculate on the advantages of axonal collateral within the other ganglion cell classes. In this regard, it is noteworthy that several subdivisions which receive projections via the same size class of ganglion cells also interconnect. For example, the cells of the SGS_S send projections primarily to two thalamic targets, the LGNd and LGNv (Fitzpatrick et al., '80). Within the LGNd, SGS_S cells project mainly to layers 3 and 6 and all but one of the interlaminar zones. As mentioned earlier, cells within LGNd layer 3, and to some extent layer 6, also respond to both the ON-set and OFF-set of light and receive input predominantly from small ganglion cells. At present, it is not obvious why the cells in LGNd layers 3 and 6 would receive both direct and indirect projections from ON-OFF gamma-like ganglion cells. One possibility is that the indirect pathway from the colliculus increases the likelihood that the small LGNd cells will reach threshold, perhaps communicating the continued presence of stimuli of interest in one part of the visual field in preparation for an eye or a head movement. This information could then be relayed to cortical layers III and I and act to modulate the visual signals coming to these cells from cortical layer IV which may be more concerned with the detailed, spatial properties of the visual stimulus.

ACKNOWLEDGEMENTS

We are grateful to Drs. Sherre Florence, Judy Brunso-Bechtold and George Condo for helpful suggestions and for reading the manuscript at different stages of completion. We thank Drs. Elizabeth Birecree and Lance Durden for technical help in various stages of this study, Aurora Buck for her skillful care and handling of the animals, Julie Mavity for photographic assistance, Vera Murphy for typing and to Sam Marvin for proofing the manuscript. This research was supported by Public Health Service Grant EY01778 and was performed in partial fulfillment of the requirements for the degree of Doctor of Philosophy from Vanderbilt University. E.J. DeBruyn was supported by Public Health Service Fellowship MH08472 and by Neuroscience Training Grant MH15452. V.A. Casagrande was supported by Research Career Development Award K04-EY00223, and J.T. Weber by EY01277 to Dr. John Harting.

REFERENCES

Abplanalp, P. 1971 The neuroanatomical organization of the visual system in the tree shrew. Folia Primat., 16:1-34.

Albano, J.E., A.L. Humphrey, and T.T. Norton 1978 Laminar organization of receptive field properties in the tree shrew superior colliculus. J. Neurophysiol., 41:1140-1164.

Albano, J.E., T.T. Norton, and W.C. Hall 1979 Laminar origins of projections from the superficial layers of the superior colliculus in the tree shrew, *Tupaia glis*. Brain Res., 173:1-11.

Boycott, B.B., and H. Wässle 1974 The morphological types of ganglion cells in the domestic cat's retina. J. Physiol. (Land.), 240:397-419.

Brecha, N., and H.J. Karten 1979 Accessory optic projections upon oculomotor nuclei and vestibulocerebellum. Science, 203:913-916.

Bunt, A.H., R.D. Lund, and J.H. Lund 1974 Retrograde axonal transport of horseradish peroxidase by ganglion cells of the albino rat's retina. J. Comp. Neurol., 164:265-286.

Bunt, A.H., A.E. Hendrickson, J.S. Lund, R.D. Lund, and A.F. Fuchs 1975 Monkey retinal ganglion cells: Morphometric analysis and tracing of axonal projections with a consideration of the peroxidase technique. J. Comp. Neurol., 164:265-286.

Casagrande, V.A., and E.J. DeBruyn 1982 The Galago visual system: Aspects of normal organization and developmental plasticity. In: The Lesser Bushbaby (Galago) as an animal model: selected topics. Ed., D.E. Haines, CRC Press, Boca Raton, Fla., pp. 137-181.

Casagrande, V.A., and J.K. Brunso-Bechtold 1985 Development of lamination in the lateral geniculate nucleus; critical factors. In: Advances in neural and behavioral development. Vol. 2, ed., N. Aslin, Ablex Publ. Corp., N.J.

Casagrande, V.A., R.W. Guillery, and J.K. Harting 1978 Differential effects of monocular deprivation seen in different layers of the lateral geniculate nucleus. J. Comp. Neurol., 1979:469-486.

Chalupa, L.M., and I. Thompson 1980 Retinal ganglion cell projections to the superior colliculus of the hamster demonstrated by the horseradish peroxidase technique. Neurosci. Lett., 19:13-19.

Conley, M., D. Fitzpatrick, and I.T. Diamond 1984 The laminar organization of the lateral geniculate body and the striate cortex in the tree shrew (*Tupaia glis*). J. Neurosci., 4:171-197.

Conley, M., E.A. LaChica, and V.A. Casagrande 1985 Demonstration of ipsilateral retinocollicular projections in the tree shrew (*Tupaia glis*). Brain Res., 346:181-185.

Conway, J.L., and P.H. Schiller 1983 Laminar organization of tree shrew dorsal lateral geniculate nucleus. J. Neurophysiol., 50:1330-1342.

DeBruyn, E., and V.A. Casagrande 1978 Density and central projections of retinal ganglion cells in the tree shrew. Soc. for Neurosci. Abstr., 4:642.

DeBruyn, E.J., and V.A. Casagrande 1986a The organization of retinal ganglion cells in the tree shrew (*Tupaia belangeri*). I. Analysis of cell size and distribution. J. Comp. Neuro. (Submitted).

DeBruyn, E.J., and V.A. Casagrande 1986b The organization of Retinal ganglion cells in the tree shrew (*Tupaia belangeri*): II. Analysis of Cell Morphology. J. Comp. Neurol. (Submitted).

Dreher, B., Y. Fukuda, and R.W. Rodieck 1976 Identification, classification, and anatomical segregation of cells with X-like and Y-like properties in the lateral geniculate nucleus of Old-World primates. J. Physiol. (Lond.), 258:433-452.

Farmer, S.G., and R.W. Rodieck 1982 Ganglion cells of the cat accessory optic system: morphology and retinal topography. J. Comp. Neurol, 205:190-198.

Finger, T.E., and H.J. Karten 1978 The accessory optic system in teleosts. Brain Res., 153:144-149.

Fitzpatrick, D., R.G. Carey, and I.T. Diamond 1980 The projection of the superior colliculus upon the lateral geniculate body in *Tupaia glis* and *Galago senegalensis*. Brain Res., 194:494-499.

Fitzpatrick, D., K. Itoh, and I.T. Diamond 1983 The laminar organization of the lateral geniculate body and the striate cortex in the squirrel monkey (*Saimiri sciureus*). J. Neurosci., 3:673-702.

Fukuda, Y., and J. Stone 1974 Retinal distribution and central projections of Y-, X-, and W-cells of the cat's retina. J. Neurophysiol., 37:749-772.

Graham, J., and V.A. Casagrande 1980 A light microscopic and electronmicroscopic investigation of the superficial layers of the superior colliculus of the tree shrew (*Tupaia glis*). J. Comp. Neurol., 191:133-151.

Hanker, J.S., P.E. Yates, C.B. Metz, and A. Rustioni 1977 A new, specific, sensitive, and non-carcinogenic reagent for the demonstration of horseradish peroxidase. J. Histochem., 9:789-792.

Harting, J.K., W.C. Hall, I.T. Diamond, and G.F. Martin 1973 Anterograde degeneration study of the superior colliculus in (*Tupaia glis*): Evidence for a subdivision between the superficial and deep layers. J. Comp. Neurol., 148:361-386.

Hofbauer, A., and U.C. Dräger 1985 Depth segregation of retinal ganglion cells projecting to the mouse superior colliculus. J. Comp. Neurol., 234:465-474.

Hoffman, K.P., and A. Schoppmann 1975 Retinal input to direction selective cells in the nucleus tractus opticus of the cat. Brain Res., 99:359-366.

Hoffman, K.P., and A. Schoppmann 1981 A quantitative analysis of the direction-specific response of neurons in the cat's nucleus of the optic tract. Exp. Brain Res., 42:146-157.

Huerta, M.F., J.T. Weber, L.R. Rothstein, and J.K. Harting 1985 Subcortical connections of area 17 in the tree shrew: an autoradiographic analysis. Brain Res., 340:163-170.

Hughes, A. 1981 Population magnitudes and distribution of major modal classes of cat retina ganglion cells as estimated from HRP filling and a systematic survey of the soma diameter spectra for classical neurons. J. Comp. Neurol., 197:303-339.

Humphrey, A. L., and J. S. Lund 1979 Anatomical organization of layer IV in tree shrew striate cortex (area17): evidence for two sublaminae. Soc. for Neurosci. Abstr., 5:787.

Illing, R.-B 1980 Axonal bifurcation of cat retinal ganglion cells as demonstrated by retrograde double labelling with fluorescent dyes. Neurosci. Lett., 19:125-130.

Illing, R.-B, and H. Wässle 1981 The retinal projection to the thalamus in the cat: A quantitative investigation and a comparison with the retinotectal pathway. J. Comp. Neurol., 202:265-285.

Irvin, G.E., T.T. Norton, and T.K. Kuyk 1983 Morphology of physiologically defined neurons in the superior colliculus of the tree shrew. Invest. Ophthalmol. Vis. Sci. Suppl., 24:224.

Irvin, G.E., T.T. Norton, M.E. Sesma, and V.A. Casagrande 1986 W-like response properties of interlaminar zone cells in the lateral geniculate nucleus of a primate (Galago crassicaudatus). Brain Res., 362:254-270.

Itoh, K., M. Conley, and I.T. Diamond 1981 Different distribution of large and small retinal ganglion cells in the cat after HRP injections of single layers of the lateral geniculate body and the superior colliculus. Brain Res., 207:147-152.

Itoh, K., M. Conley, and I.T. Diamond 1982 Retinal ganglion cell projections to individual layers of the lateral geniculate body in Galago crassicaudatus. J. Comp. Neurol., 205:282-290.

Jones, E.G., and R.Y. Leavitt 1974 Retrograde axonal transport and the demonstration of non-specific projections to the cerebral cortex and striatum from thalamic intralaminar nuclei in the rat, cat, and monkey. J. Comp. Neurol., 154:349-378.

Kaas, J.H. 1985 Structural basis for information processing in the primate visual system. In: Visual Neuroscience, Eds., I.P. Pettigrew, W.R. Levick, and K.J. Sanderson. London: Cambridge University Press.

Karten, J.H., K.V. Fite, and N. Brecha 1977 Specific projection of displaced retinal ganglion cells upon the accessory optic system in the pigeon (Columbia livia). Proc. Natl. Acad. Sci., U.S.A., 74(4):1753-1756.

Kelly, A.S., and P.C. Fox 1977 A quantitative study of ascending and descending inputs to the rabbit lateral geniculate nucleus and superior colliculus. Soc. for Neurosci. Abstr., 3:565.

Kelly, J.P., and G.D. Gilbert 1975 The projections of different morphological types of ganglion cells in the cat retina. J. Comp. Neurol., 163:65-80.

Kimm, J., J.A. Winfield, and A.E. Hendrickson 1979 Visual-vestibular interactions and the role of the flocculus in the vestibule-ocular reflex. Frog. Brain Res., 50:703-713.

Koontz, M.A., R.W. Rodieck, and S.G. Farmer 1985 The retinal projection to the cat pretectum. J. Comp. Neurol., 236:42-59.

Kuyk, T.K., J.T. Siegwart, and T.T. Norton1982 Receptive field properties of ventral LGN neurons in tree shrew. Invest. Ophthalmol. Vis. Sci., Suppl. 22(3):117.

Laemle, L.K. 1968 Retinal projections of *Tupaia glis*. Brain Behav. Evol., 1:473-499.

Lane, R.H., J.M. Allman, and J.H. Kaas 1971 Representation of the visual field in the superior colliculus of the grey squirrel (*Sciurus carolinensis*) and the tree shrew (*Tupaia glis*). Brain Res., 26:277-292.

LaVail, J.H., and M.M. LaVail 1974 Retrograde intraaxonal transport of horseradish peroxidase in retinal ganglion cells of the chick. Brain Res., 85 (2):273-280.

LeVay, S., and S.K. McConnell 1982 ON and OFF layers in the lateral geniculate nucleus of the mink. Nature, 300:350.

Livingstone, M.S., and D.H. Hubel 1984 Specificity of intrinsic connections in primate primary visual cortex. J. Neurosci., 4:2830-2835.

McConnell, S.K., and S. LeVay 1983 Geniculocortical afferents in the mink: evidence for ON/OFF and ocular dominance patches. Soc. for Neurosci. Abstr., 9:617.

Molotchnikoff, S., P. Lachapelle, D. Richard, P. L'Archeneque, and I. Lessard 1979 Latency distribution from orthodromic stimulation at the optic nerve, in the lateral geniculate nucleus and superior colliculus of rabbits. Brain Res. Bull., 4:579-581.

Montgomery, N., K. Fite, and L. Bengston1979 The anuran accessory optic system: Neuroanatomical analysis. Soc. for Neurosci. Abstr., 5:798.

Nelson, R., E.V. Famiglietti, and H. Kolb 1978 Intracellular staining reveals different levels of stratification of ON- and OFF-center ganglion cells in cat retina. J. Neurophysiol., 41:472-483.

Norton, T.T., G. Rager, and R. Kretz 1985 ON and OFF regions in layer IV of striate cortex. Brain Res·. 327:319-323.

Oyster, C.W., E.S. Takahashi, and P.E. Soodak 1980 Retinal ganglion cells projecting to the rabbit accessory optic system. J. Comp. Neurol., 190:49-61.

Peichl, L., and H. Wässle 1981 Morphological identification of ON- and center brisk transient OFF (Y) cells in the cat retina. Proc. Roy. Soc. (Lond.), 212:139-153.

Polyak, S. 1957 The vertebrate visual system. University of Chicago Press, Chicago, IL.

Rapaport, D.H., and P.D. Wilson 1983 Retinal ganglion cell size groups projecting to the superior colliculus and the dorsal lateral geniculate nucleus in the North American opossum. J. Comp. Neurol., 213:74-85.

Reiner, A. 1981 A projection of displaced ganglion cells and giant ganglion cells to the accessory optic nuclei in turtle. Brain Res., 204:403-409.

Reiner, A., and H.J. Karten 1978 A bisynaptic retinocerebellar pathway in the turtle. Brain Res., 150:163-169.

Reiner, A., N. Brecha, and H.J. Karten 1979 A specific projection of retinal displaced ganglion cells to the nucleus of the basal optic root in the chicken. Neurosci., 4:1679-1688.

Rhoades, R.W., and L.M. Chalupa 1978 Conduction velocity distribution of the retinocollicular pathway in the golden hamster. Brain Res., 159:396-401.

Rodieck, R.W., and R.K. Brening 1983 Retinal ganglion cells: properties, types, genera, pathways, and trans-species comparisons. Brain Behav. Evol., 23:121-164.

Schiller, P.H. 1984 The connections of the retinal ON and OFF pathways to the lateral geniculate nucleus of the monkey. Vis. Res., 24:923-932.

Sesma, M.A., V.A. Casagrande, and J.H. Kaas 1985 Cortical connections of area 17 in tree shrews. J. Comp. Neurol., 230:337-351.

Simpson, J.I. 1984 The accessory optic system. Ann. Rev. Neurosci., 7:13-41.

Spear, P.D., D.C. Smith, and L.L. Williams 1977 Visual receptive-field properties of single neurons in cat's ventral lateral geniculate nucleus. J. Neurophysiol., 40:390-409.

Spitzer, H., and S. Hochtein 1985 Simple and complex cell response dependences on stimulation parameters. J. Neurophysiol., 53(5):1244-1265.

Steindler, D.A. 1982 Differences in the labelling of axons of passage by wheat germ agglutinin after uptake by cut peripheral nerve versus injections within the central nervous system. Brain Res., 250:159-167.

Stryker, M.P., and K.R. Zahs 1983 On and off sublaminae in the lateral geniculate nucleus of the ferret. J. Neurosci., 3:1943-1951.

Ter Laak, H.J., and J.M. Thijssen 1978 Receptive field properties of optic tract fibers from ON-center sustained and transient cells in a tree shrew (Tupaia chinensis). Vis. Res., 18:1097-1109,

Tigges, J. 1966 Ein experimenteller beitrag zurn subkortikalen optischen system von *Tupaia glis*. Folia Primatol., 4:102-123.

Torrealba, F., G.D. Partlow, and R.W. Guillery 1981 Organization of the projection from the superior colliculus to the dorsal lateral geniculate nucleus of the cat. Neurosci., 6:1341-1360.

Van Dongen, P.A., H.J. Ter Laak, J.M. Thijssen, and A.J.H. Vendrik 1976 Functional classification of cells in the optic tract of a tree shrew (*Tupaia chinensis*). Exp. Brain Res., 24:441-446.

Vanegas, H., H. Hollander, and H. Distel 1978 Early stages of uptake and transport of horseradish peroxidase by cortical structures, and its use for the study of local neurons and their processes. J. Comp. Neurol., 177:193-212.

Wässle, H., and R.-B. Illing 1980 The retinal projection to the superior colliculus in the cat: A quantitative study with HRP. J. Comp. Neurol., 190:333-356.

Wässle, H., B.B. Boycott, and R.-B. Illing 1981 Morphological and mosaic of ON- and OFF-beta cells in the cat retina and some functional consideration. Proc. Roy. Soc. (Lond.) B, 212:177-195.

Weber, J.T., and J.K. Harting 1980 The efferent projections of the pretectal complex: an autoradiographic and horseradish peroxidase analysis. Brain Res., 194:1-28.

Wilson, P.D., and J. Stone 1975 Evidence of W-cell input to the cat's visual cortex via the C laminae of the lateral geniculate nucleus. Brain Res., 92:472-478.
.

Winer, J.A. 1977 A review of the status of the horseradish peroxidase method in neuroanatomy. Biobehav. Rev., 1:45-54.
