# Supplementary material for: DeBruyn and Casagrande manuscripts on tree shrew retinal ganglion cells as a basis for cross-species retina research
Source: Vis Neurosci. 2022 Jan 31;39:E001. doi: 10.1017/S0952523821000171 (PMC8807137; doi:10.1017/S0952523821000171)
Supplement: Supplementary file 1 [file vnssup.zip › S0952523821000171sup002.pdf]

The Organization of Retinal Ganglion Cells in the Tree Shrew (*Tupaia belangeri*): II. Analysis of Cell Morphology

E.J. DeBruyn[1,2] and V.A., Casagrande[1,3]

Departments of Cell Biology[1] and Psychology[3]

Vanderbilt University

Nashville, TN 37232

[2]Present Address: Department of Electrical Engineering

Vanderbilt University

Nashville, TN 37235

Running Head: Tree Shrew Ganglion Cell Morphology

Address Correspondence to: Dr. V.A. Casagrande

Department of Cell Biology

Vanderbilt University

Nashville, TN 37235

Key words: Tree shrew, retina, ganglion cells, area centralis, visual streak, cell morphology.

ABSTRACT

In a companion study we examined the topographic distribution of cell size and density in the retinal ganglion cell layer of tree shrews (*Tupaia belangeri*) (DeBruyn & Casagrande, '86). The results show that the retina of tree shrews contains specialized regions exemplified by variations in cell density, mean cell size, and cell size range. Such variations undoubtedly reflect functional specialization, which, in turn, may be supported by a differential distribution of morphological classes of ganglion cells. In order to examine this possibility, we analyzed the morphological structure of ganglion cells that were back-filled with horseradish peroxidase. Well-filled cells selected for analysis were taken from both areas of central specialization (the *area centralis* and visual streak) and more peripheral regions within nasal and temporal retina. Qualitative examination of these cells suggested that they could be divided into three major classes which were termed types I, II and III. These three classes resemble, respectively, the alpha, beta, and gamma classes described for cat by Boycott and Wässle ('74); and can be distinguished based upon quantitative differences in regional distribution, soma size, axon diameter and primary dendritic field size. Type I cells have large somata, axons, and dendritic fields and are distributed relatively uniformly across the retina with some increase in relative frequency in the periphery. Type II cells have medium somata and axons, small, highly-branched dendritic fields, and show the greatest relative concentration in the *area centralis*. Type III cells have small somata, thin axons, large sparsely branched dendritic fields, and are found with the greatest frequency in the visual streak and visual periphery.

In order to apply a more objective approach to classification of these cells we performed a hierarchical cluster analysis using quantitative measures of soma area, axon diameter, primary dendritic field area, and number of primary dendrites of each cell. This form of analysis suggested that the cells fell into five major morphological clusters. Comparison of our qualitative classification and cluster analysis suggested a rough correspondence. Clusters 1 and 2 contained mainly type I cells; cluster 3 contained mainly type II cells and clusters 4 and 5 contained mainly Type III cells. Comparison between our morphological ganglion cell types and physiological ganglion cell classes defined in three shrews (Van Dongen et al., '76; Ter Laak & Thijssen, '78) indicates that clusters 2 and l may correspond to transient and direction selective physiological classes, respectively, while cluster 3 (or Type II) cells may correspond

with the sustained cell class.· These conclusions are reinforced by our analysis of the differences in central projections of ganglion cells in tree shrews presented in a companion paper (DeBruyn et al., '86).

INTRODUCTION

Our recent studies and those of others have provided strong evidence that retinal ganglion cells in tree shrews are specialized to transmit different types of visual information in parallel to central visual targets. Tree shrew ganglion cells exhibit a variety of physiological properties, are distributed unequally over the retinal surface in terms of size and density, and project to thalamic and midbrain targets with distinct physiological properties (Sherman et al., '75; Van Dongen et al., '76; Albano et al., '78; DeBruyn and Casagrande, '86; DeBruyn et al., '86). The main goal of the present study was to examine the morphology of horseradish peroxidase (HRP) filled ganglion cells in tree shrews to determine if distinct anatomical types could be differentiated, as might be expected from previous evidence.

As a natural outgrowth of our efforts to classify ganglion cell morphology in tree shrews, we became concerned with methods used to classify morphological cell types. Since this subject has been covered in depth from a number of different perspectives (e.g., Rowe and Stone, '77), our goal was not to invent a new scheme but rather to construct a means that would allow for both reasonable cross-species morphological comparisons and for more direct comparisons with physiological ganglion cell classes described for tree shrews (Van Dongen et al., '76; Ter Laak and Thijssen, '78). Therefore, a second goal of the study was to use both a qualitative and a quantitative (cluster analysis) classification to determine which morphological cell grouping best fit available comparative and physiological data.

Our results show that retinal ganglion cells in tree shrews can be divided into three qualitatively defined groups that share many characteristics in common with the alpha, beta and gamma ganglion cells of the cat retina (Boycott and Wässle, '74). Cluster analysis reveals that two of the three qualitatively defined groups may be further subdivided, and that these subgroups have physiological correlates.

METHODS

The study was based on results from four adult tree shrews (*Tupaia belangeri*) two of which (81-43, 81-62) received iontophoretic injections of HRP into their superior colliculi (SCs), and two of which (81-117, 82-21) received pressure injections of HRP into the optic tract. In each case, an effort was made to sample representative numbers of cells from the two areas of specialization, the *area centralis* and the visual streak, as well as from the peripheral retina. Of the 166 cells sampled from six retinae, 35 were from the *area centralis* (defined as the area enclosed by the 16,000 cell/mm^2 isodensity line), 65 were from the visual streak (defined as the area bounded centrally by the 16,000 cell/mm^2 isodensity line and peripherally by the 9,000 cell/mm^2 isodensity line), and 66 were from the peripheral retina (defined as any region of the retina with a density of less than 9,000 cells/mm^2). Of these 66 peripheral cells, 23 were sampled in the temporal retina and 43 in the nasal retina. Figure 1 shows the locations of all cells sampled.

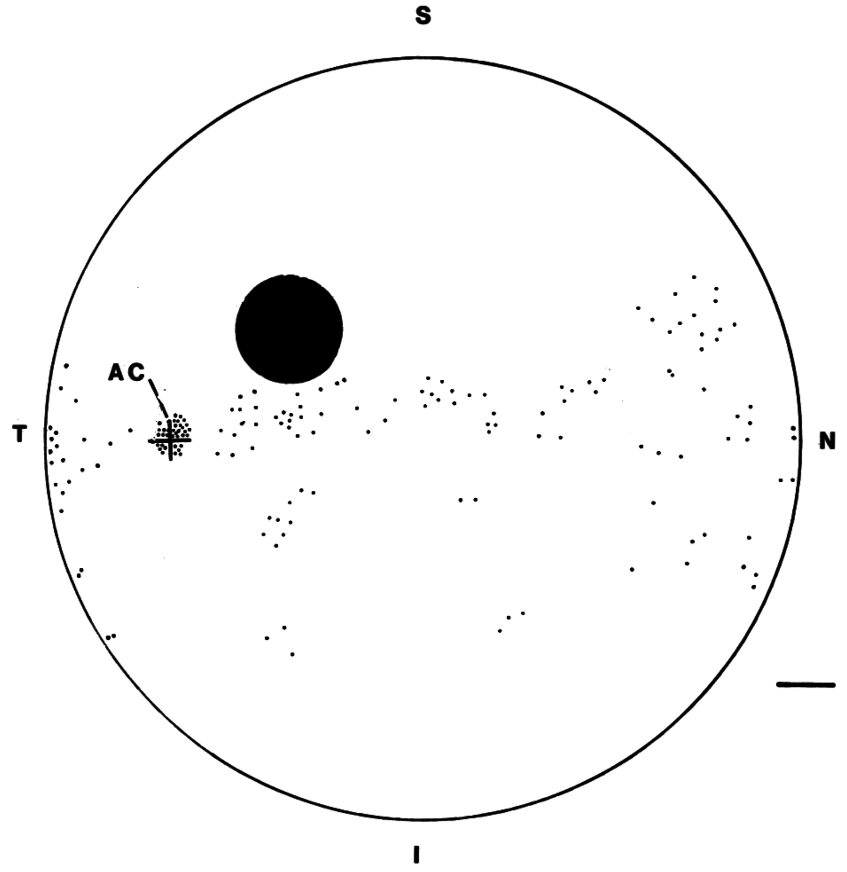

Figure 1. Figure 1. A schematic representation of the approximate locations of all 166 morphologically identified ganglion cells represented on a generalized retinal whole mount. The position of the *area centralis* is represented by the cross, and the optic disc is filled. Scale= 1 mm. AC - *area centralis*; I - inferior; N - nasal; S - superior; T - temporal.

Surgical and Histological Procedures

Each animal was deeply anesthetized with pentobarbital (Nembutal, 55 mg/kg), placed in a Kopf stereotaxic apparatus, modified for tree shrews, and a craniotomy was performed. A portion of the posterior cortex overlying the optic tract or SC was removed by gentle aspiration using a glass pipette. Pressure injections of 0.5 µl of 50% HRP (Sigma, type IX) in distilled water or saline were made into one optic tract using a Hamilton 5 µl syringe fitted with a 30-gauge needle. Iontophoretic injections were made using glass micropipettes (type diameter 20-50 µm) filled with 30% HRP in saline. Small amounts of HRP were injected by the application of a 2-3 µ amp current (pipette tip negative) for 20-30 minutes (1/sec; 50 msec duration). Following a 2-day survival period, the animals were re-anesthetized, enucleated, and perfused transcardially with saline followed sequentially by: 2.5% glutaraldehyde in 0.l M phosphate buffer (pH 7.4); 5% sucrose in 2.5% buffered glutaraldehyde and 10% sucrose in a buffer rinse. The retinae were then removed from the eyes, immersion fixed in 2.5% buffered glutaraldehyde and the vitreous was removed. They were then rinsed and reacted in Hanker-Yakes reagent (Hanker et al., '77) and whole-mounted as described previously (DeBruyn & Casagrande, '86). Following dehydration and coverslipping, backfilled ganglion cells were drawn at l000X using a *camera lucida* drawing tube (Zeiss), and soma areas, areas of the primary dendritic fields, S-DF (soma-dendritic field) ratios (see also below), axon diameters, and the number of primary dendrites were determined. Retinae were subsequently counterstained to obtain total cell density measures.

In order to evaluate injection sites, the brains were frozen-sectioned at 52 µm on a sliding microtome and every section through the injection site and selected sections throughout the remainder of the brain were reacted with Hanker-Yates reagent and counterstained with cresyl violet.

Measurements

The analysis of the present data required the use of techniques which have been employed in previous studies of retinal ganglion cell morphology (e.g., Boycott and Wässle, '74) as well as several new concepts. The analyses of soma area, number of primary dendrites, and axon diameters were made directly from drawings of the cells at 1000X and the axon diameters were measured at the point at which the axon left

the hillock. The primary dendritic field of a ganglion cell was defined as the area enclosed by a polygon constructed by joining the first branch points of each dendrite with straight lines. In cases in which this technique could not be employed, as in bipolar type cells or *area centralis* cells with one primary dendrite, the polygon was constructed by connecting the first branch points with the widest points of the cell soma. We used a measure of primary dendritic field, rather than the more traditional total dendritic field extent, in order to eliminate artifactual size differences caused by incomplete filling of the dendrites with HRP. The S-DF (the soma/primary dendritic field) ratio was developed in order to compare cells from different retinal eccentricities. It assumes, that all classes of ganglion cells show systematic variation with eccentricity, an idea supported by our own analysis (see Fig. 5). Given this assumption, the S-DF ratio should allow common classes of cells to be grouped regardless of retinal position.

All measurements described above were performed with the aid of the Bioquant II computerized image analysis system (Leitz).

Statistical Analysis

A Student's t-test was performed in all cases in which two experimental groups were present. Comparisons involving three or more groups were tested using a one-way analysis of variance. The validity of the characteristics chosen as predictors of cell type was confirmed using factor analysis (Kaiser and Caffry, '65). This test determines the percentage of variance that each characteristic contributes to the total morphological variance. Factors that contribute little to the variance are viewed as insignificant and can be eliminated from subsequent analyses.

Other quantitative comparisons were accomplished by using a hierarchical cluster analysis (Johnson, '67), which separates cells into groups or clusters based on a set of morphological characteristics. Each cell is first assigned a position in multi-dimensional space based on the value of its characteristics. The space is then collapsed and those cells which lie closest to each other are grouped together to form clusters. This process is then repeated until all cells are grouped into one large cluster. At any particular point in this procedure, a cluster is defined as a group of cells in which the average distance between cells is less than the minimum distance to the next closest cluster. Assuming that close distances represent

similar sets of characteristics, it is clear that the most dissimilar cells will be the most widely spaced and thus, will be the last to cluster together.

RESULTS

Examination of the retinal ganglion cells reveals a rich variety of shapes, sizes, and styles of dendritic configuration (see Fig. 2). However, it is difficult to approach this material without the tendency to group, categorize, or classify these cells into types. Perhaps, because of our familiarity with well-known descriptions of cat ganglion cells (e.g., Boycott and Wässle, '74), or because of similarities between the morphological ganglion cell types of cats and tree shrews, we found that the ganglion cells of tree shrews were most easily grouped into three qualitative types. For convenience, we will refer to these as Types I, II and III, which resemble cat alpha, beta, and gamma cells, respectively, (Boycott & Wässle, '74). Figure 2 shows typical examples of the three types.

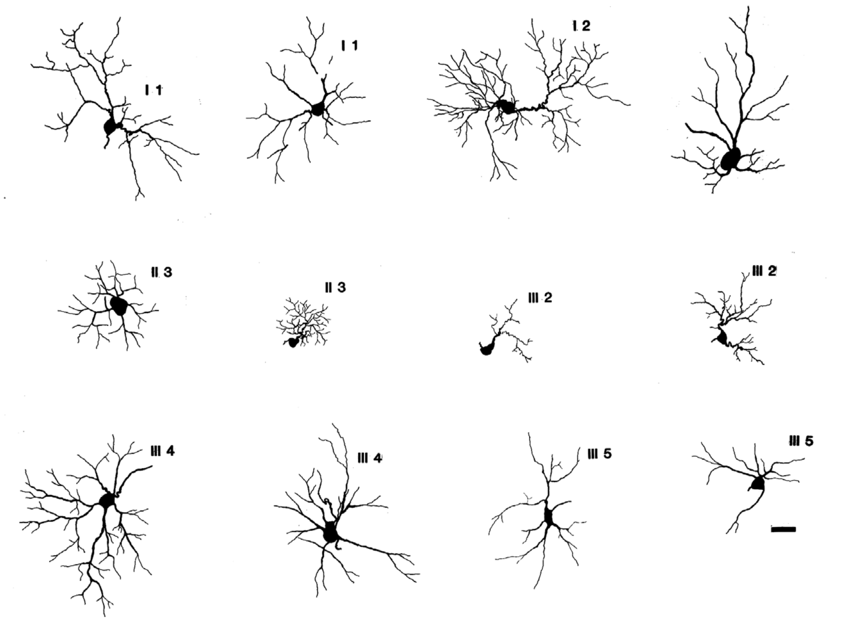

Figure 2. Examples of cell types from the tree shrew retina. Camera lucida drawings of morphological Types I, II, and III. The subclass cluster of each cell is indicated by the number next to the morphological type. Scale = 25 μm.

Type I cells-have large somas, large primary dendritic fields, and thick axons, in contrast, Type II cells have small somas, small primary dendritic fields, and medium-sized axons. Finally, Type III cells have small somas, large primary dendritic fields (with some exceptions such as bipolar types) and thin axons. In addition to morphological characteristics, each of these cell types exhibits similar trends to those described in the cat (Boycott and Wässle, '74). All three types are larger in the peripheral retina than in the *area centralis* (Figure 3), and the distribution of each type varies across the retina such that Type I cells are proportionally more common in the periphery, Type II cells are more common in the *area centralis,* and

Type III cells are least common in the *area centralis* (table 1). No tendency was noted for any cell type to concentrate within the visual streak as has been described in the cat (Rowe and Stone, '76, but see Hughes, '81).

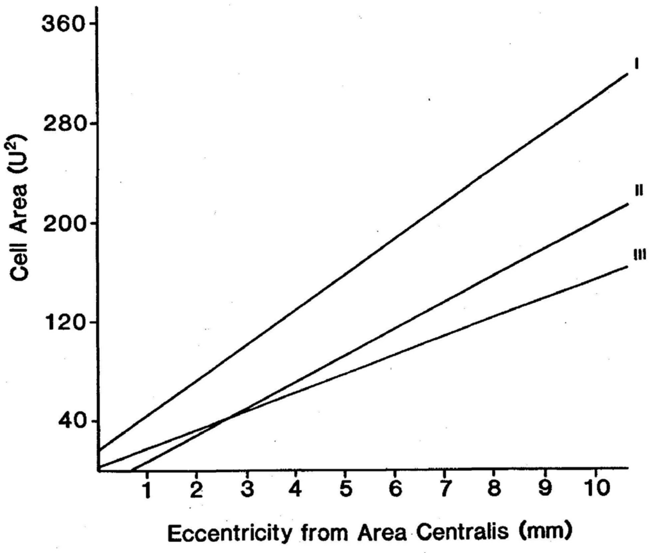

Figure 3. The relationship of soma size to eccentricity from the *area centralis*. Samples were taken at 1 mm intervals in a horizontal line across the retina. At each point sampled, the soma sizes of 15-20 cells of each type were measured. Note that the slope of the regression line for Type I cells is steepest, indicating that these cells have the greatest proportional increase in size with increasing eccentricity. In contrast, the slope of the regression line for Type III cells is the shallowest, indicating that the soma size of these cells change the least with increasing eccentricity.

In order to provide a more objective description of the three qualitatively defined cell types, each cell sampled was quantitatively evaluated in terms of five different characteristics: soma size, primary dendritic field size, axon diameter, and number of primary dendrites and the S-DF ratio. In the following sections each cell type is compared in terms of these characteristics.

Type I Cells - Figure 4 shows that Type I cells have the largest somata of the three groups (mean = 204.35 ± 8.15 μm^2; range = 89.14 to 400.90 μm^2), large primary dendritic fields (mean = 766.59 ± 45.6 μm^2; range = 144.82 to 1968.27 μm^2), thick axons (mean 0.99 ± 0.03 μm; range = 0.5 to 1.5 μm), 3 to 5 primary dendrites (mean = 3.77 ± 0.07) and intermediate S-DF ratio values (mean = 3.91 ± 0.21; range = 0.85 to 9.97). In all cases except the number of primary dendrites, the frequency histograms of these parameters form bimodal distributions (Fig. 4a, b, c, d). This trend is particularly evident in the case of axon diameter in which the histogram is distinctly bimodal with peaks occurring at 0.9 μm and 1.3 μm.

This bimodality suggests that two subclasses of ganglion cell may exist within this cell population, a point which we will return to later.

TABLE 1

THE RELATIVE PROPORTIONS OF CELL TYPES WITHIN REGIONS OF SPECIALIZATION AND THE PERIPHERAL RETINA

			Percentage of Cell Types		
Retinal Region	Case	N[a]	Type I	Type II	Type III
Area Centralis	81-117C	42	16.6	78.5	4.7
	81-117I	41	14.6	82.9	2.4
	82-21C	43	23.2	67.4	9.3
	81-21I	39	15.3	76.9	7.6
Visual Streak	81-117C	39	25.6	43.5	30.7
	81-117I	--	----	----	----
	82-21C	39	22.5	45.0	32.5
	82-21I	--	----	----	----
Peripheral Retina	81-117C	45	35.5	31.1	33.3
	81-117I	21	42.8	52.3	4.7
	82-21C	43	39.5	30.2	30.2
	82-21I	24	41.6	50.0	8.3

Table 1. The relative proportions of ganglion cell types I-III in different retinal regions. The data represent all ganglion cells that could be positively classified in 2-5 adjoining 0.1 X 0.1 mm fields taken from the center most densely labelled portion of each region. Note the large percentage of Type II cells encountered within the area centralis, and the relatively small percentage of Type III cells in this area. Note also that the percentage of Type III cells is no higher within the visual streak than in the peripheral regions of nasal retina (cases 81-117C, 82-21C). Finally, note the small percentage of Type III cells encountered in the temporal periphery (cases 81-117I, 82-21I).

[a]Number of cells in each area.

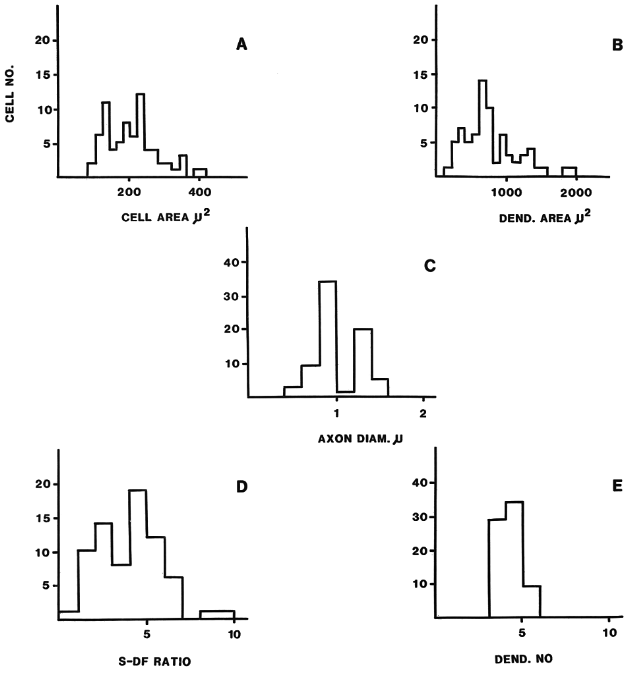

Figure 4. Frequency histograms of the five morphological characteristics (soma size (A), primary dendritic field size (B), axon diameter (G), S-DF ratio (D) and number of primary dendrites (E) used to classify Type I cells in the present study. Note that in all cases except the number of primary dendrites (E), the histograms are bimodal in form, suggesting the presence of more than one subclass of cell within this type. This trend is especially notable in the case of axon diameter (G).

201

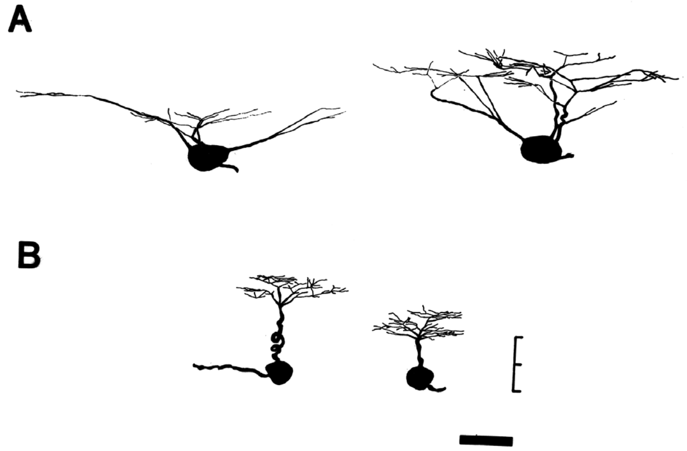

Figure 5. Dendritic stratifications of two (A) Type I (cluster 2) and two (B) Type II (cluster 3) ganglion cells illustrating the difference in the level of dendritic stratification. Note that two levels of dendritic stratification are evident in both cell types. Due to the problem of positively identifying laminar and sublaminar boundaries in whole mounted retinae, the borders of the IPL and its sublaminae have not been illustrated. Scale= 20 µm.

Reconstruction of the level of dendritic stratification of a few cells from each of these types suggested that the population may be further divisible based on the level of arborization in the inner plexiform layer. Figure 5 shows examples of cells that clearly restrict their terminal arborization to either the superficial or deeper tier of the inner plexiform layer.

Type II Cells - Type II cells (Figure 6) have small somas (mean = 121.19 ± 5.54 μm^2; range = 65.08 to 239.41 μm^2), small primary dendritic fields (mean = 203.24 ± 18.87 μm^2; range = 13.80 to 617.58 μm^2), medium-sized axons (mean = 0.82 ± 0.04 μm; range = 0.4 to 1.7 μm), 1 to 4 primary dendrites (mean = 1.47 ± 0.09) and low S-DF ratio values (mean = 1.56 ± 0.09; range = 0.11 to 3.19). In contrast to Type I cells, the frequency histograms are unimodal indicating the presence of only one cell type. However, as with Type I cells, reconstructions of the primary and second dendrites of a few of these cells suggested that they, too, formed two subpopulations with terminations restricted to either the superficial or deep portion of the inner plexiform layer (see Figure 5).

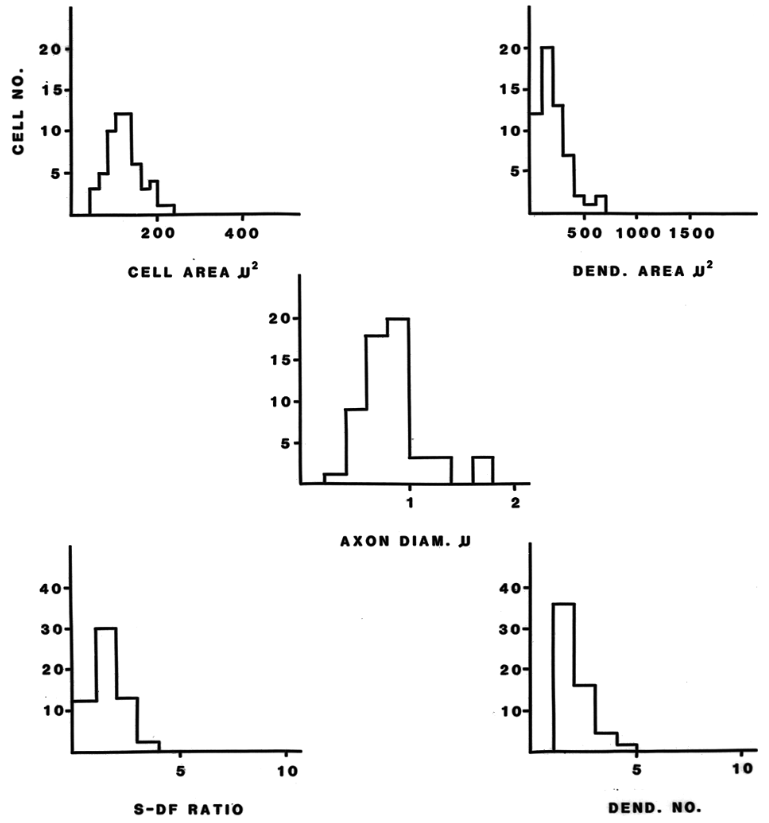

Figure 6. Frequency histograms of the morphological characteristics of Type II ganglion cells. Note that the histograms form unimodal curves indicating the presence of only one subclass of cell. Compare with Figure 4.

Type III Cells - Figure 7 shows that these cells are characterized by small somata (mean = 121.35 ± 8.29 μm^2; range= 31.89 to 228.23 μm^2), very large primary dendritic fields (mean = 1037.72 ± 128.12 μm^2; range= 107.56 to 3429.27 μm^2), thin axons (mean = 0.55 ± 0.03 μm; range= 0.2 to 1.3 μm), 2 to 7 primary dendrites (mean 3.72 ± 0.16) and large S-DF ratio values (mean 8.12 ± 0.64; range= 1.06 to 18.36). The outstanding characteristics of this cell type is the large amount of variance revealed by frequency distributions (Figures 7a-e); this heterogeneity possibly indicative of the presence of several subclasses of retinal ganglion .cells. This situation is similar to that reported for cat gamma cells (Boycott and Wässle, '74; Fukuda and Stone, '74; Kolb et al., '81).

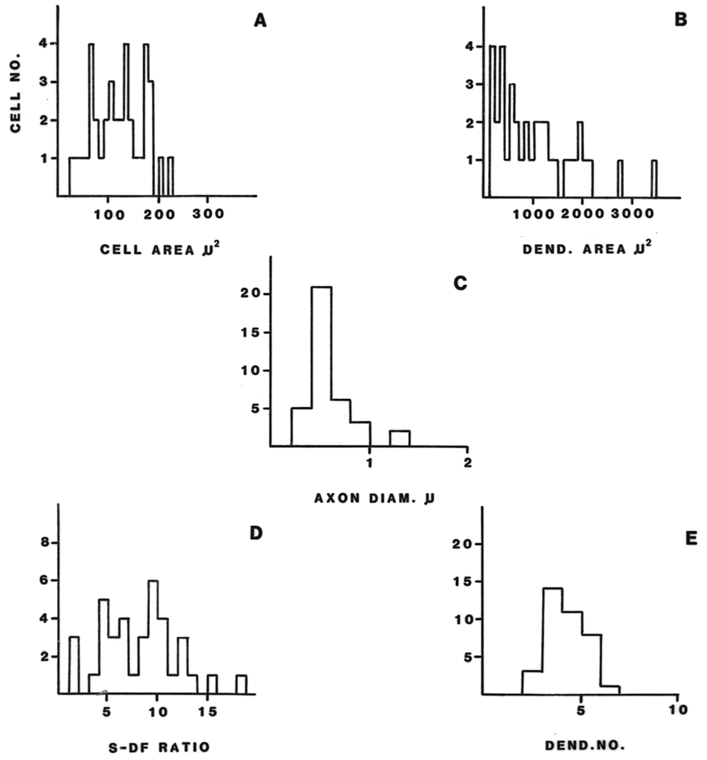

Figure 7. Frequency histograms of the morphological characteristics of Type III ganglion cells. Note the presence of multiple peaks in histograms A, B, and D, indicating this class of cell, like Type I cells, may be composed of more than one subclass. Compare with Figures 4 and 6.

Cluster Analysis

In order to determine if tree shrew retinal ganglion cells fall into similar morphological groupings if strictly quantitative criteria were applied, we performed a hierarchical cluster analysis. This procedure organizes the cells into groups based upon similarities in selected morphological parameters (see also Methods). When the total sample of 166 cells was subjected to this analysis, five clusters (comprising 164 cells or

98.7% of the total sample) and two individual cells were obtained. Comparison between qualitatively classified cell Types I, II and III and the five clusters revealed a surprisingly good fit (Figure 8). Clusters

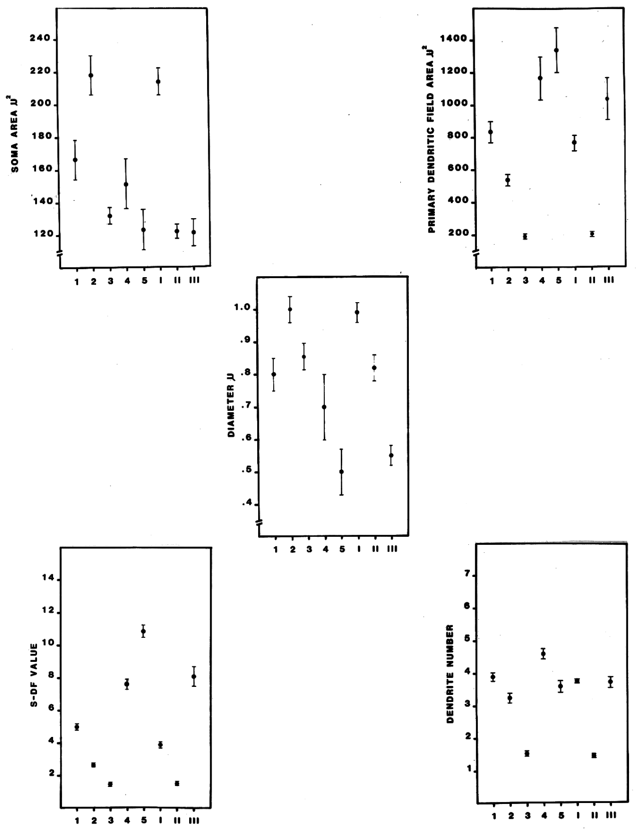

Figure 8. Comparisons of the qualitative and cluster analysis classifications. The mean value (± S.E.M.) of each of five parameters is plotted against the different clusters (1-5) or subjective Types (I-III) for comparison, Note the correspondence between clusters 2, 3 and 5 and Types I, II and III, respectively.

1 and 2 include 81 cells of which 65 (80%) are qualitatively defined Type Is, 10 (12%) are Type Ills and 6 (8%) are Type IIIs. Thus, clusters 1 and 2 together can be thought of as being roughly equivalent to Type I cells. Inspection of the data suggests that the following characteristics separate cells in cluster 1 and 2 from each other. Cluster 1 cells have smaller somata (mean = 166.47 ± 12.02 μm^2), larger dendritic fields (mean = 836.00 ± 64.47 μm^2), thinner axons (mean = 0.8 ± 0.05 μm) and higher S-DF values (mean = 4.99 ± .12) than do cluster 2 cells (somas - mean = 208.50 ± 11.84 μm^2; dendritic fields - mean = 540.02 ± 36.75 μm^2; axons - mean = 1.0 ± 0.04 μm; S-Df - mean = 2.64 ± 0.15). (See Figure 8).

In contrast to clusters 1 and 2, cluster 3 consists of a more homogeneous population, which contains mainly Type II cells. Of the 58 cells in this cluster, 53 (92%) are Type II cells, 3 (5%) are Type I cells and 2 (3%) are Type III cells. Moreover, the two Type III cells that fell into the cluster 2 group are

unusual in configuration, being bipolar types. The most characteristic feature of cells in cluster 3 is the low S-DF ratio (mean 1.46 ± .09). The similarity between this value and the corresponding one for Type II cells (1.47 ± .09, Fig. 8), further reinforces the close correspondence between the qualitative and the cluster analysis classification schemes of these particular cells.

Clusters 4 and 5 are appear to be included in one qualitatively classified cell type, namely, Type III. Of the 25 cells in these clusters, 20 (80%) are Type Ills, while the other 5 (20%) are Type Is. Most of the latter Type I cells are included in cluster 4, making up 40% (4 of 10 cells) of its population, while only one (6%) Type I cell is included in cluster 5. Thus, cluster 4 is unique in being made up of almost an equal percentage of Type I and III cells. In both clusters 4 and 5, however, the characteristic features are the same, namely, small- to medium-sized somata (4 - mean = 151.64 ± 15.24 μm^2 ; 5 - 123.69 ± 12.32 μm^2), large dendritic fields (4 - mean - 1166.57 ± 130.67 μm^2; 5 - mean 1339.42 ± 138.94 μm^2), high S-DF ratios (4 - mean = 7.66 ± 0.29 μm^2; 5 - mean = 10.85 ± 0.32 μm^2) and thin axons (4 - mean = 0.7 ± 0.1 μm; 5 - mean 0.5 ± 0.07 μm). As can be seen in figure 8, the two clusters are, themselves, differentiated mainly on the basis of soma and dendritic field size. Cluster 4 cells have larger somata and smaller dendritic fields than cluster 5 cells.

Finally, the two unclustered cells were qualitatively classified as Type IIIs. Their exclusion from the other clusters was due primarily to their extremely large dendritic fields (2722.07 μm^2 and 3429.27 μm^2) and large S-DF values (15.76 and 18.36).

Classification Based on Size

Since ganglion cell size has been considered as a useful indicator of physiological type, at least in cats (see Boycott and Wässle, '74; Saito, '83; Stanford and Sherman, '84), it is worthwhile to consider how well it distinguishes between ganglion cell types and clusters in tree shrews.

Since all cell types exhibit an increase in mean soma size with increasing eccentricity from the *area centralis* (see above), the value of absolute soma size as a predictor of morphological type is severely limited. A more useful size correlate of morphological type is size relative to the local cell population. Using this measure, we compared the relative soma sizes of 44 cells (5 of each qualitative type from each area of retinal specialization, with the exception that only 4 Type III cells were available from the *area*

centralis) and expressed these as a percentile score. Figure 9 shows that when the results are expressed this way, Type I cells are the largest (mean = 89.6 ± 2.19 %tile; range = 72nd - 99th %tile) and show a clear separation from Types II and III, whose distributions overlap considerably. However, even in the latter two groups, there is some size segregation with Type II cells being larger (mean = 44.9 ± 3.07 %tile; range= 25th - 62nd %tile) than Type III cells (mean = 29.5 ± 4.44 %tile; range = 8th - 69th %tile).

Figure 9 also shows the relationship between relative soma size and cell cluster. As would be predicted from the foregoing, relative size does not distinguish well between cells in clusters 1 and 2, but only between both of these clusters and the remaining cells. Since cluster 3 and Type II refer to an almost identical population, the medium size of these cells can, on average be distinguished from those in clusters 1, 2 and 5. It is difficult to make predictions about the relative size of cluster 4 cells since only one was included in this analysis. However, if this cell is representative, then cluster 4 and 5 cells can clearly be distinguished based on relative size.

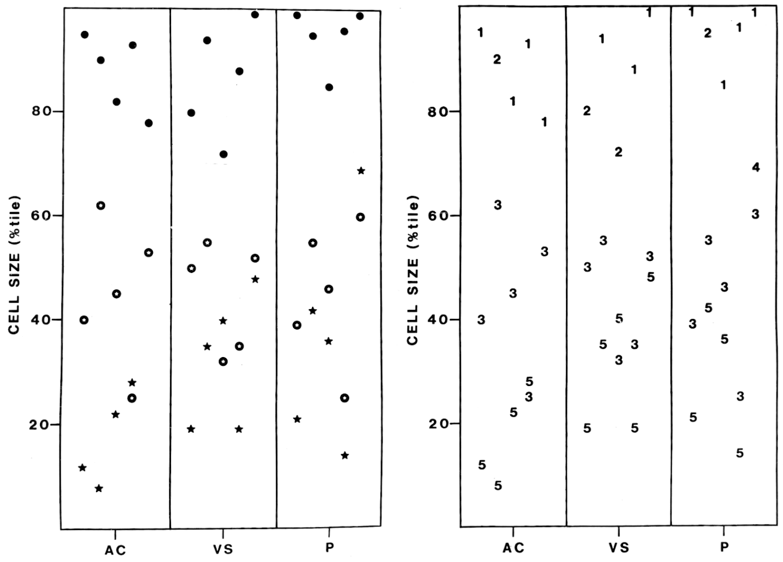

Figure 9. Correlations between cell size and cell classification. The relative sizes (in comparison with the local population) of types I (filled circles), II (circled stars), and III (filled stars) are indicated for three retinal regions (*area centralis*, visual streak and peripheral retina) in the left figure. Note that at all locations, Type I cells have the large somas, Type II cells have medium-sized somas and Type III cells have small somas. A similar correspondence between cluster and cell size is evident in the right figure. Note that clusters 1 and 2 are large in size, while clusters 3 and 4 are medium in size and cluster 5 cells are small. A.C. – *area centralis*; P - periphery; V.S. - visual streak.

Thus, the different morphological types of tree shrew ganglion cells do show some separation by soma size, although this separation is less clear than in species such as the cat (Boycott and Wässle, '74).

DISCUSSION

We have distinguished three main morphological types of ganglion cells in tree shrews, which can be further divided into five classes based upon a cluster analysis. Before discussing the significance of these findings in relationship to what is known about ganglion cells in other species, and about the physiology of ganglion cells in tree shrews, it is worthwhile to consider the rationale behind our classification schemes.

Retinal ganglion cells in mammals have been subdivided into types based upon a number of different schemes (for review see Perry, '82; Rodieck and Brening, 1 83). By far, the most common approach has been to characterize cell types in rather qualitative terms noting obvious similarities and differences. In particular, it has been common practice to try to relate qualitatively defined cell types in other species to the descriptions of alpha, beta, and gamma cells in cats (Boycott and Wässle, '74). Such a practice is useful since there is now clear evidence that the morphological cell types in cats are, for the most part, physiologically distinct and have morphological counterparts in other species (Rodieck and Brening, '83; Saito, 83; Stanford and Sherman, '84). Therefore, it seemed reasonable to begin by establishing if similar morphological types existed in tree shrews. Another advantage to beginning our study with a qualitative description of ganglion cell types was that this approach taps the obvious pattern discrimination powers of our own visual system.

However, the difficulty with such a purely subjective approach is that it is much easier to detect differences in cells than to quantify such differences once detected. Without some form of quantification, it is very difficult to establish the basis of one's cell typing scheme. The usefulness for understanding the role of established cell types is, thus, severely limited as is translation across species. Therefore, we undertook two separate approaches to quantify morphological difference. In the first, we began with qualitative cell types and measured features to quantify their differences; in the second, we reversed the process, quantifying features and then asking whether cells possessing different collections of such features would cluster into groups. The first approach has, as mentioned, the advantage of the power of human pattern discrimination and past experience, while the second approach has the advantage of knowing exactly what went into the formula. The disadvantage of both approaches is that in neither case can we be assured that the morphological types generated indicate functionally distinct cell classes. Regardless, the close match

(84%) between our cell types defined qualitatively and by cluster analysis strengthens the view that our morphological ganglion cell types define different classes of cells in tree shrews.

Comparison with Other Species

Tree shrew retinal ganglion cell types exhibit a number of similarities to the morphological ganglion cell classes reported in other species. The most clear-cut example is our Type II (or cluster 3 cells), which appear to be similar to the beta cells of the cat (Boycott and Wässle, '74; Kolb et al., '81); and equivalent cell types have been noted in almost every species thus far studied (Dogiel, 1891, 1893; Cajal, '33; West, '76; Perry and Cowey, '81; Amthor et al., '83; Wilson and Condo, '85; Vitek et al., '85). However, beta cells are absent in some species such as rats (Perry, '79). In macaque monkeys, cells with beta-like morphology vary considerably from the parafovea where they exhibit very compact dendritic fields (midget ganglion cells) to the periphery where they resemble more closely our Type II cells (Leventhal et al., '81; Perry et al., '84). Tree shrew cells, however, more closely resemble the morphological variations seen in the cat (Boycott and Wässle, '74), with no forms resembling primate midget ganglion cells.

Analogies can also be drawn between our Type I cells and alpha-like cells described in a variety of species (Dogiel, 1891, 1893; Polyak, '57; West, '76; Leventhal et al., '81; Perry and Gowey, '81; Amthor et al. , '83; Perry et al., '84; Vitek et al., '85). It is more difficult to relate the cluster subclasses (1 and 2) of our Type I cells to distinct cell types described in other species since morphological divisions have not been emphasized among cells with large somata. The epsilon cells in cat (Leventhal et al., '81) and E or PE cells in primates (see Figure 2, Leventhal et al., '81 or Figure 7, Perry and Gowey, '84) are perhaps closest in description to our cluster 1 our cluster 1 cells since cluster 1 axons are finer, their dendritic fields larger and their somata smaller than cluster 2 cells which are more similar to cat alpha cells.

Our Type III cells resemble cat gamma cells in the broadest definition of the term (Boycott and Wässle, '74). Not surprisingly, gamma-like cells have been identified in all species investigated (e.g., Boycott and Wässle, '74; Kolb et al., '81; Amthor et al., '83). The difficulty with this loosely defined category is that it undoubtedly contains several subclasses, but efforts to subdivide this group of cells have been, for the most part, only partially successful. Our cluster analysis suggests that these cells contain two subclasses (4 and 5). Of these two subclasses, cells belonging to cluster 5, with their very small somata,

fine axons and sparse widely radiating dendrites, are most similar in appearance to small cat gamma cells. A few also resemble the g1 and g2 cells described for the cat and ferret (Leventhal et al., '85; Vitek et al., '85). Cluster 4 cells do not neatly translate to classes described in other animals, but some certainly resemble the large cat gamma cells (epsilon cells), or P, C, or unclassified cells in macaque monkeys (Leventhal et al., '81; Perry and Cowey, '84).

These cross-species comparisons suggest that the majority of morphological profiles of retinal ganglion cells and major cell types we have identified in tree shrew exist in other mammalian species. At present it is less clear whether our cluster subclasses are unique to tree shrews or exist in other species since quantitative cluster analyses have not been performed on ganglion cells in other species.

There are, however, some cell types that have been identified in other species that we have not seen in our material. For example, we have not seen any ganglion cells with small somata, complex dendritic arbors and spines characterized in cats as delta cells (Boycott and Wässle, '74); nor have we identified counterparts to ON-OFF Type II ganglion cells in rabbit with their characteristic "loops" of retroflexively-branched dendrites (Amthor et al., '83). However, since both of the latter cell types depend for identification on specialized distal features of dendrites, not finding them may simply reflect lack of filling in the finest segments of the dendrites. On the other hand, we have identified several bipolar cells (Type II, but not cluster 3) which have not been described in other species such as cats (Kolb et al., '81) but may exist in the rabbit (Amthor et al., '83).

Functional Implications

The consistency of the morphological correlates of a given physiological cell type across species strongly suggests that the morphology of a cell influences its physiological characteristics. More specifically, the dendritic morphology can influence the physiological properties of a cell in at least two ways. First, the extent (and to some degree, the pattern of dendritic branching), sets limits on the synaptic contacts that a cell can make. Obviously, a Type I or III cell with its wide dendritic field has the opportunity to contact cells over a much larger region of the retina than does a Type II cell in which the dendritic field is more restricted. Likewise, the level of ramification of the dendrites within the inner plexiform layer determines which types of bipolar cells (hyper- or depolarizing) a cell can synapse with (Famiglietti and Kolb, '76).

Second, the pattern of dendritic branching has been found to be an important determinant of the passive electrical properties of a cell (Rall and Rinzel, '73; Rall, '77; Koch et al., -'82). Rall ('77, see also Rall and Rinzel, '73) has reported that when current is injected into the end of a dendrite, the magnitude of the voltage attenuation is much higher than when the same current is injected into the soma. This difference in attenuation depends on the number of branches and is non-existent for cases in which no branches are present, suggesting that the pattern of dendritic branching plays an important role in integrating incoming information. These data suggest that cells with similar dendritic morphology will be found to have similar physiological characteristics and may account for the trans-species consistency in morphology. It is noteworthy, however, that the converse of this suggestion is not necessarily true. Evidence suggests that a set of physiological characteristics can be constructed from more than one morphological substrate. For example, in the cat the physiological correlate of the beta cell is the X-cell. However, some cat retinal X-cells that do not exhibit the morphology of beta cells have been recently described (Stanford and Sherman, '84). Moreover, rat ganglion cells with X-like physiology have been reported, yet cells with beta-like morphology are not found among rat ganglion cells (Fukuda, '77; Perry, '79).

Correlation with Physiological Types of Tree Shrew RGCs

Studies of tree shrew retina have established the existence of eight physiological types of ganglion cells (Van Dongen et al., '76; Thijssen et al., '76; Ter Laak and Thijssen, '78). Of these eight types, two (sustained and transient cells) can be equated to X and Y cells in the cat (Enroth-Cugell and Robson, 1 66), while the other six (on-off center, suppressed by contrast, orientation-selective, direction-selective, color opponent, and edge inhibitory-off-center) are similar to cat W-cells (Stone and Hoffmann, '72; Fukuda and Stone, '74).

With respect to the first two cell types, it is likely that sustained cells correspond to our type II or cluster 3 cells for several reasons. First, both sustained and Type II cells are concentrated around the *area centralis* (present results, Van Dongen et al., '76). Second, sustained cells have the smallest receptive field center sizes of any tree shrew retinal ganglion cell (Van Dongen et al., '76), while Type II cells have the smallest dendritic field sizes of our morphological subclasses. Third, a correlation has been made between sustained retinal ganglion cells and cell types with morphology similar to Type II cells in cat and monkey

(Boycott and Wässle, '74; Fukuda and Stone, '74, '75; Perry and Gowey, '81; Saito, '83; Stanford; Sherman, '84).

The morphological correlate of the physiologically defined transient cells is less clear. In cat, (Boycott and Wässle, '74; Fukuda and Stone, '74, '75) and monkey (Perry and Gowey, '81) brisk transient ganglion cells (Y and Y-like cells) have been correlated with ganglion cells with large cell bodies and alpha morphology. Moreover, Van Dongen et al. ('76) reported that transient cells in tree shrew are more evenly distributed over the retina, a characteristic shared by large ganglion cells in the present study. Since tree shrews possess two subclasses of cells with large cell somas, clusters 1 and 2, either or both could be the morphological correlate of transient cells. However, two characteristics, axon diameter and dendritic field size, provide some evidence for separating the two clusters. With respect to axon diameter, it is first necessary to state that Sherman et al. ('75) found a clear separation between lateral geniculate nucleus X- (sustained) and Y- (transient) cells in terms of their response latency to chiasm stimulation, implying a clear separation in the axon diameters of the two types. Since the axons of cluster 2 cells are significantly larger than those of Type II and cluster 3 cells (the presumptive correlate of X-cells), while those of cluster 1 cells are not, it seems reasonable to assume that cluster 2 cells with their larger axons may be the correlate of the transient cells. Similarly, in terms of receptive field size, Van Dongen et al. ('76) reported that transient cells had smaller receptive field centers than did direction selective cells, the other potential candidate for large ganglion cells (see below). Since cluster 2 cells have smaller dendritic field sizes than cluster 1 cells, it seems likely that cluster 2 cells are the morphological correlates of transient cells.

Correlations with the remaining six physiological cell types are more tenuous. One might speculate, however, that of six cell types, 2 have characteristics that are compatible with the presently described morphological classes. The first is the direction-selective cell which may be represented by our cluster 1 cells. Previous studies of the rabbit (Oyster et al., '81), pigeon (Karten et al., '77), and cat (Grasse and Cynader, '80**)** have shown that direction-selective cells project to the medial terminal nucleus (MTN) of the accessory optic system or its homolog in the avian system. Furthermore, in numerous species (rabbit, Oyster et al., '80; pigeon, Karten et al., '77; turtle, Reiner and Karten, '78; fish, Finger and Karten, '78) cells projecting to this nucleus have been shown to have large somas. Since our own studies (DeBruyn et al., '86), have demonstrated a similar projection of large ganglion cells to the MTN in tree shrews, one could

argue that direction-selective cells are the physiological correlates of cluster 1 cells. It should be noted, however, that the percentage of ganglion (less than 5%) projecting to the MTN is less than the percentage (16%) of direction-selective cells reported by Van Dongen et al.,'76.

A second physiological cell type that might have a morphological correlate is the orientation-selective cell. Van Dongen et al. ('76) reported that these cells have asymmetric receptive fields with the long axis being at least twice the length of the short axis. Assuming that receptive field shape mimics that of the cell's dendritic field (Peichl and Wässle, '81), the most obvious candidate for an orientation-selective cell would be the bipolar ganglion cells of cluster 3 (Type III) which possess definite major and minor axes in their dendritic fields. Correlates of other physiological types must await further study.

We have provided evidence for the existence of at least three morphological types of ganglion cells in the tree shrew retina which can be partially separated of the basis of cell size. Since different types of ganglion cells project differentially to central targets in other species (e.g., Illing and Wässle, '81; Leventhal et al., '81 Rodieck and Brening, '83), the logical extension of this study is to examine the central projections of tree shrew ganglion cells. In the following paper, we report on the projections of different sized ganglion cells to different subcortical nuclei.

ACKNOWLEDGEMENTS

We are grateful to Drs. Sherre Florence and Judy Brunso-Bechtold for helpful suggestions and for reading the manuscript at different stages of completion. We thank Drs. Elizabeth Birecree and Lance Durden for technical help in various stages of this study, Aurora Buck for her skillful care and handling of the animals, Julie Mavity for photographic assistance, Vera Murphy for typing and to Sam Marvin for proofing the manuscript. Special thanks to Dr. Michael Sesma for his help with the cluster analysis material. This research was supported by Public Health Service Grant EY017778 and was performed in partial fulfillment of the requirements for the degree of Doctor of Philosophy from Vanderbilt University. E.J. DeBruyn was supported by Public Health Service Fellowship MH08472 and by Neuroscience Training Grant MH15452. V.A. Casagrande was supported by Research Career Development Award K04-EY00223

REFERENCES

Albano, J.E., A.L. Humphrey, T.T. Norton 1978 Laminar organization of receptive field properties in the tree shrew superior colliculus. J. Neurophysiol., 41:1140-1164. 25.

Amthor, F.R., C.W. Oyster, and E.S. Takahashi 1983 Quantitative morphology of rabbit retinal ganglion cells. Proc. Roy. Soc. (Lond.) B., 217:341-355.

Boycott, B.B., and H. Wässle 1974 in the domestic cat's retina. The morphological types of ganglion cells J. Physiol. (Lond.), 240:397-419.

Cajal, **S.** Ramon y 1933 The structure of the retina. S.A. Thorpe and M**.** Glickstein, eds., Charles C. Thomas, Springfield, Ill., 1972.

DeBruyn, E.J., and V.A. Casagrande 1986 The organization of retinal ganglion cells in the tree shrew (*Tupaia belangeri*): I. Analysis of cell size and distribution. J. Comp. Neurol., (submitted).

DeBruyn, E.J., J.T. Weber and V.A. Casagrande 1986 The organization of retinal ganglion cells in the tree shrew (*Tupaia belangeri*): III. Central Projections of Ganglion Cells. J. Comp. Neurol., (submitted).

Dogiel, A.S. 1891 Uber der nervosen Elemente in der Retina des Menschen. Arch. f. Mikroskop. Anat., 38:317-344.

Dogiel, A.S. 1893 Uber der nervosen Elemente in der Retina des Menschen. Arch. f. Mikroskop. Anat., 40:29-38.

Enroth-Cugell, C., and J. Robson ganglion cells of the cat. 1966 The contrast sensitivity of retinal J. Physiol. (Lond.), 187:518-552.

Famiglietti, E.V., and H. Kolb 1976 Structural basis for "ON" and "OFF"-center responses in retinal ganglion cells. Science, 194:193-195.

Finger, T.E., and H.J. Karten 1978 The accessory optic system in teleosts. Brain Res., 153:144-149.

Fukuda, Y. 1977 A three-group classification of rat retinal ganglion cells: Histological and physiological studies. Brain Res., 119:327-344.

Fukuda, Y., and J. Stone 1974 Retinal distribution and central projections of Y-, X- and W-cells of the cat's retina. J. Neurophysiol., 37:749-772.

Grasse, K.L., and M.S. Cynader 1980 Directional selectivity in the medial terminal nucleus of the cat. Soc. for Neurosci. Abstr. 6:122.

Hanker, J.S., P.E. Yates, C.B. Metz, and A. Rustioni 1977 A new, specific, sensitive, and non-carcinogenic reagent for the demonstration of horseradish peroxidase. J. Histochem., 9:789-792.

Hughes, A., 1981 Population magnitudes and distribution of major modal classes of cat retinal ganglion cells as estimated from HRP filling and a systematic survey of the soma diameter spectra for classical neurons. J. Comp. Neural., 197:303-339.

Illing, R.-B., and H. Wässle 1981 The retinal projection to the thalamus in the cat: A quantitative investigation and a comparison with the retinotectal pathway. J. Comp. Neural., 202:265-285.

Johnson, S.C. 1967 Hierarchical clustering schemes. Psychometrika, 32:241-254.

Kaiser, H.F., and J. Caffry 1965 Alpha factor analysis. Psychometrika, 30:1-14.

Karten, J.H., K.V. Fite, and N. Brecha 1977 Specific projection of displaced retinal ganglion cells upon the accessory optic system in the pigeon (Columbia livia). Proc. Nat. Acad. Sci., U.S.A., 74(4):1753-1756.

Koch, C., T. Poggio, and V. Torre 1982 Retinal ganglion cells: A functional interpretation of dendritic morphology. Phil. Trans. Roy. Soc. (Land.) B, 298:227-263.

Kolb, H., R. Nelson, and A. Mariani 1981 Amacrine cells, bipolar cells, and ganglion cells of the cat retina: A Golgi study. Vision Res., 21:1081-1114.

Leventhal, A.G., R.W. Rodieck, and B. Dreher 1981 Retinal ganglion cell classes in cat and Old-World monkey: morphology and central projections. Science, 213:1139-1142.

Leventhal, A.G., R.W. Rodieck, and B. Dreher 1985 Central projections of cat retinal ganglion cells. J. Comp. Neural., 237:216-226.

Oyster, C.W., E.S. Takahashi, and D.C. Hurt 1981 Density, soma size and regional distribution of rabbit retinal ganglion cells. J. Neurosci., 1:1331-1346.

Peichl, L., and H. Wässle 1981 Morphological identification of ON- and OFF- center brisk transient (Y) cells in the cat retina. Proc. Roy. Soc. (Lond.) B, 212:139-156.

Perry, V.H. 1979 The ganglion cell layer of the retina: A Golgi Study. Proc. Roy. Soc. (Land.) B, 204:363-375.

Perry, V.H. 1982 The ganglion cell layer of the mammalian retina. Prog. In Retinal Research (eds., N. Osborne and G. Chader) Vol. 1, pp. 53-80.

Perry, V.H., and A. Gowey 1981 The morphological correlates of .X- and Y- retinal ganglion cells in the retina of monkeys. Exp. Brain Res., 43:226-228.

Perry, V.H., and A. Gowey 1984 Retinal ganglion cells that project to the superior colliculus and pretectum in the macaque monkey. Neurosci, (12)5:1125-1137.

Perry, V.H., R. Oehler, and A. Gowey 1984 Retinal ganglion cells that project to the dorsal lateral geniculate nucleus in the macaque monkey. Neurosci. (12)4:1101-1123.

Polyak, S. 1957 The vertebrate visual system. University of Chicago Press, Chicago, Ill.

Rall, W. 1977 Core conductor theory and cable projections of neurons. In: Handbook of Physiology (eds. E. Kandel and S. Geiger), pp. 39-97., American Physiological Society, Washington, D.C.

Rall, W., and J. Rinzel 1973 Branch input resistance and steady attenuation for input to one branch of a dendritic neuron model. Biophys. J., 13:648-688.

Reiner, A., and H.J. Karten 1978 A bisynaptic retinocerebellar pathway in the turtle. Brain Res., 150:163-169.

Rodieck, R.W., and R.K. Brening 1983 Retinal ganglion cells: properties, types, genera, pathways, and trans-species comparison. Brain Behav. Evol. 23:121-164.

Rowe, M.H., and J. Stone 1976 Properties of ganglion cells in the visual streak of the cat's retina. J. Comp. Neural., 169:99-126.

Rowe, M.H., and J. Stone 1977 Naming of neurons. Classification and naming of cat retinal ganglion cells. Brain Behav. Evol, 14:185-216.

Saito, H. 1983 Morphology of physiologically identified X-, Y-, and W-type retinal ganglion cells of the cat. J. Comp. Neural., 221:279-288.

Sherman, S.M., T.T. Norton, and V.A. Casagrande 1975 X- and Y-cells in the dorsal lateral geniculate nucleus of the tree shrew (Tupaia glis). Brain Res., 93:152-157.

Stanford, L.R., and S.M. Sherman 1984 retinal ganglion cells in the cat. Structure/function relationships of Brain Res. 297:381-386.

Stone, J., and K.-P. Hoffman 1972 Very slow-conducting ganglion cells in the cat's retina: a major new functional type? Brain Res., 43:610-616.

Ter Laak, H.J., and J .M. Thijssen 1978 Receptive field properties of optic tract fibers from ON-center sustained and transient cells in a tree shrew (*Tupaia chinensis*). Vision Res., 18:1097-1109.

Thijssen, J.M., P.A.M. Van Dongen, and H.J. Ter Laak 1976 Maintained activity of cells in the tree shrew optic tract. Exp. Brain Res., 25:279-290.

Van Dongen, P.A., H.J. Ter Laak, J.M. Thijssen, and A.J.H. Vendrik 1976 Functional classification of cells in the optic tract of a tree shrew (*Tupaia chinensis*). Exp. Brain Res., 24:441-446.

Vitek, D.J., J.D. Schall, and A.G. Leventhal 1985 Morphology, central projections, and dendritic field orientation of retinal ganglion cells in the ferret. J. Comp. Neural., 241:1-11.

West, R. 1976 Light and electron microscopy of the ground squirrel retina: functional considerations. J. Comp. Neural., 168:355-378.

Wilson, P.D., and G.J. Condo 1985 the North American Opossum. Beta-like ganglion cells in the retina of Brain. Res., 331:155-159.
