# Supplementary material for: DeBruyn and Casagrande manuscripts on tree shrew retinal ganglion cells as a basis for cross-species retina research
Source: Vis Neurosci. 2022 Jan 31;39:E001. doi: 10.1017/S0952523821000171 (PMC8807137; doi:10.1017/S0952523821000171)
Supplement: Supplementary file 1 [file vnssup.zip › S0952523821000171sup001.pdf]

The Organization of Retinal Ganglion Cells in the Tree Shrew (*Tupaia belangeri*). I. Analysis of Cell Size Distribution

E.J. DeBruyn[1,2] and V.A., Casagrande[1,3]

Departments of Cell Biology[1] and Psychology[3]

Vanderbilt University

Nashville, TN 37232

[2]Present Address: Department of Electrical Engineering

Vanderbilt University

Nashville, TN 37235

Running Head: Tree Shrew Retinal Ganglion Cells

Address Correspondence to: Dr. V.A. Casagrande

Department of Cell Biology

Vanderbilt University

Nashville, TN 37235

Key words: Tree shrew, retina, ganglion cells, area centralis, visual streak, cell size, cell density.

ABSTRACT

The objective of this study and two companion studies (DeBruyn and Casagrande, '86; DeBruyn et al., '86) was to describe, quantify, and classify retinal ganglion cells in the tree shew (*Tupaia belangeri*). In this paper, the distribution and sizes of retinal ganglion cells were analyzed using whole mounted Nissl-stained retinae. Our data show that the ganglion cell layer is characterized by a well-developed visual streak, an *area centralis* located close to the temporal retinal margin, and no fovea. Both the *area centralis* and visual streak contain a high density of smaller cells. This density reaches a peak of approximately 20,000 cells/mm^2 in the area centralis. Cell density declines and mean cell area and range of cell areas increases in the periphery (more rapidly for temporal than for nasal retina) but remains high (3000 cells/mm^2) even at the extreme margins of the retina. Additionally, centroperipheral density gradients in temporal retina are steeper than those in nasal retina such that at points of equal eccentricity from the *area centralis* in the nasal and temporal retina (i.e., points that view the same point in the visual field), cell density in the nasal retina is always higher than in the temporal retina. Cell size analysis shows that, in general terms, cell area is inversely related to cell density. Mean cell size ranges from 45µm^2 in the *area centralis* to over 100µm^2 in peripheral retina. Nasal-temporal differences are also apparent in distributions of cell size such that cells in the nasal retina are significantly smaller than their temporal counterparts at eccentricity-matched points. At density-matched points, nasal cells are not significantly smaller than temporal cells, although more large (75th-99th% of local cell sizes) cells are present in temporal retina.

Taken together, our data suggest that the retina of tree shrews is specialized both for binocular and monocular visual resolution, based upon the existence of an *area centralis* in the binocular retina, and the visual streak and high density of ganglion cells in the extreme periphery which relate to monocular viewing. This organization is best explained by the visual adaptations unique to the tree shrew, not by its proposed relationship to primates as suggested originally by LeGros Clark, ('59). (362 words)

INTRODUCTION

Only within the last decade has research into the organization of retinal ganglion cells progressed to the point where enough information is available on cell size, functional specialization, and central projections to begin to make generalizations concerning the basic features of retinal topography and their functional significance. Recent evidence from a number of physiological studies indicate that ganglion cells in several species can be divided into several functional subgroups with different cell sizes (Boycott and Wässle, '74; Cleland et al, '75; Fukuda, '77; Saito, '83; Stanford and Sherman, '84; see Rodieck and Brening, '83 for review.). Moreover, differences in the central projections of these cell types have led to the concept that they form three parallel functional channels (see Stone et al., '79 for review). Beyond these features, evidence demonstrates that many mammals exhibit topographic specializations within the ganglion cell layer such as an area centralis, fovea, or visual streak, which permit functional specialization in response to varied visual environments. The organization of the retina in the tree shrew is of interest for several reasons. First, there is considerable knowledge about the anatomical and functional organization of central visual structures in this species.

For example, as in cat and monkey, the dorsal lateral geniculate nucleus (LGND) of the tree shrew is laminated and contains both X- and Y-functional subclasses of cells (Laemle, '68; Casagrande and Harting, '75; Hubel, '75; Sherman et al., '75). Moreover, these cell types are known to project to layers I, III, IV and VI of visual cortex (Carey et al., '79; Conley et al., '84). Considerable information is also available on the connections and functional organization of the tree shrew superior colliculus, pulvinar, and striate and extrastriate visual areas (Lane et al., '71; Harting et al., '72, '73; Kaas et al., '72; Ohno et al., '75; Albano et al., '78, '79; Graham and Casagrande, '80). In contrast, we know little about general features of retinal organization in this animal.

The tree shrew also occupies a pivotal position in the postulated phylogeny of primates. LeGros Clark ('59) placed special emphasis on the relative development of the visual system of the Tupaiadae when he classified them as primates, yet many features of the tree shrew visual system, such as the unusual laminar arrangement of the LGND and the extreme development of the superior colliculus, are

suggestive of an evolutionary path independent of primates. Thus, it becomes of interest to compare the retinal organization in the tree shrew with that in primates.

Finally, what little we do know about the tree shrew retina suggests that it is almost totally cone dominated: less than 4% of receptors identified are rods (Immel and Fischer, '82). In support of this observation is the finding that tree shrews show only a photopic sensitivity curve with no purkinje shift (Tigges et al., '67). It is, therefore, of interest to examine how a nearly pure cone population is reflected in the organization of the ganglion cell layer and distribution of ganglion cell classes within this layer.

In this series of experiments, we used retinal whole mounts of tree shrew retinae to investigate how cells are distributed within the ganglion cell layer and how these cells can be classified based upon their size, morphology, and central projections. In this paper, the first of our series, we describe the regional variations in the densities and sizes of Nissl-stained ganglion cell somata. In the next paper (DeBruyn and Casagrande, '86), we describe the morphology of horseradish peroxidase-filled ganglion cells and develop a classification scheme based upon a cluster analysis of anatomic features. Finally, in the third paper (DeBruyn et al., '86) we describe the differences in central projections of populations of ganglion cells based upon retrograde labelling. These results have been previously presented in preliminary form (DeBruyn and Casagrande, '78; '80).

METHODS

Animals used in these experiments were adult tree shrews (*Tupaia belangeri*) weighing 120-250 gms and were raised in our colony.

Preparation of Retinal Whole Mounts

Whole mounts of six retinae from four animals were prepared in a manner similar to that of Wässle et al. ('75). Briefly, the tree shrews were anesthetized with sodium pentobarbital (Nembutal, 55 mg/kg). The eyes were then enucleated and the anterior chambers were cut away at the *ora serrata*.

The retinae were removed from the eyecups, floated onto a saline bath where any adhering pigment epithelium was brushed off, and then immersion-fixed overnight in either 10% formalin or 4% glutaraldehyde in a phosphate buffer (pH 7.4). Following this procedure, the retinae were floated onto a gelatinized slide, optic fiber layer uppermost, and several radial cuts were made around the periphery to aid in flattening the retina. Care was taken to cut at different locations in each retina so that isodensity maps would not be affected by identical patterns of shrinkage. Following flattening any remaining vitreous was removed and the retinae were covered with a piece of filter paper soaked in 5% formal-alcohol and weighted under a glass slide in a bath of the same solution overnight. The retinae were then defatted in xylene, rehydrated through alcohols, stained with cresyl violet, dehydrated, cleared in xylene, and coverslipped. It is noteworthy that immersion for 20 minutes in 3% glacial acetic acid in 100% alcohol following the first xylene step improved the quality of staining (Wässle et al., '75).

Cell Counts

Counts were made in five retinae (3788L, 79-16L, 79-16R, 79-23L, 79-23R) according to previously described methods (DeBruyn et al., '80), except that peripheral counts were taken at intervals of 0.5 mm instead of 1 mm. Moreover, in order to define more completely the density changes in the central area of the retina, cells were counted in every 0.1 mm x 0.1 mm field throughout an area of 2 mm^2 in the region of highest cell density as defined by blood vessels (Fig. 1). The total percentage of retinal surface sampled in each of the five retinae was approximately 13%.

In addition, more extensive counts were made on a sixth retina (80~15L), at intervals of 0.2 mm instead of 0.5 mm. In addition, every 0.1 mm X 0.1 mm field within an area of 5.6 mm^2 in the central retina (an area approximately enclosed by the 14,000 cell/mm^2 isodensity line) was counted. In total, approximately 40% of the retinal surface was sampled in this retina.

Construction of Isodensity Maps

In all retinae sampled, isodensity lines were drawn so that they enclosed all areas in which the counts were greater than or equal to the value assigned to the line. In addition, a three-dimensional isodensity map (Oyster et al., '81) of retina 80-15L was constructed in the following manner: the outline of the retina was traced onto a sheet of orthogonal projection graph paper so that the nasal-temporal and superior-inferior dimensions were represented in the X and Y planes, respectively. Each vertical (superior to inferior) row of sample points (starting with the most temporal row and proceeding nasally) was then plotted on the map, with the density of cells at each point being represented in the Z plane. As each row of sample points was plotted, the points were connected so that a series of density vs. position graphs for each vertical strip of retina was constructed. Isodensity lines were then drawn so they intersected each graph at the particular value assigned to the line. The use of such a procedure eliminated the need for interpolation between sample points at which cell density was somewhat higher or lower than the value of the line.

Cell Measurements

Samples of cells were taken at 1 mm intervals along the horizontal and vertical meridians. At each point sampled, the outlines of all cells in 1 to 4 0.1 mm X 0.1 mm fields were drawn at l000X using a Zeiss *camera lucida* drawing tube. Cells were included in the sample if they were estimated to have half or more than half of their profile within the field and were drawn only when their nucleoli were in focus. In addition, pairs of samples were taken at selected points in the nasal and temporal herniretinae at points matched for either eccentricity from the area centralis or for cell density. To correct for possible superior-inferior differences in cell sizes, all matched samples were taken at the same elevations above or below

the area centralis. Cell areas were measured from outlines and all statistics performed using a Bioquant II computerized image analysis system (Leitz).

Sources of Potential Error

The construction of isodensity maps necessarily involves a number of steps, any one of which may affect the accuracy of the final map. First, the number of sample points is important; maps constructed from relatively few samples clearly lack the accuracy of those in which a fine sampling matrix was used. In the present study, this problem was avoided by sampling a relatively high percentage of the retinal surface, including the entire surface within high density regions where changes in density are rapid. Second, ganglion cells must be identified correctly. Cases in which horseradish peroxidase (HRP) was used to trace central connections (see DeBruyn et al., '86) helped to set a lower limit on the size of small ganglion cells which have traditionally been the most difficult to distinguish from other cell types in the ganglion cell layer (Stone, '65, '78; Hughes, '75; Hughes and Vaney, '80; Hughes and Wieniawa Narckiewicz, '80). Nevertheless, it is possible that not all of these neurons are ganglion cells, but instead are displaced amacrine cells (Hughes and Vaney, '80) or "microneurons" (Hughes and Wieniawa-Narckiewicz, 80), that have been reported in the ganglion cell layer. Thus, the population of ganglion cells in the tree shrew may have been over-estimated. Third, in areas of high density, the existence of two or three layers of ganglion cells can make it difficult to distinguish cells. Although this factor was presumably minimized by performing all counts at l000X under oil immersion, it is possible that some cells may have been missed in the area centralis. Finally, because no sectioned retinae were examined, it is not possible to say what percentage, if any, of the ganglion cells is located in the inner nuclear layer (displaced). Since the latter have been reported to exist in a number of species (Bunt et al., '75; Karten et al., '77; Brecha and Karten, '79; Kimm et al., '79) it is possible that at least a small number may exist in the tree shrew also.

In interpreting the results of cell size data, it is necessary to take into account the variability that is inherent in these studies. For example, cell size data are subject to variation in histological technique. Hughes ('81) has reported that ganglion cells may undergo linear shrinkage of up to 20% during dehydration and staining, even in retinae in which the overall areal shrinkage is less than 5%. In areal

terms, this amounts to a 36% reduction in cell size. Moreover, cell size samples are small point-to-point measures with the large inherent variability that biological samples possess. Thus, these results should be interpreted conservatively.

RESULTS

General Appearance of the Ganglion Cell Layer

As in other species, major blood vessels emerge from the optic disc which is located in the superior temporal quadrant of the retina, 0.8 mm superior and 4.5 mm temporal to the geometric center of the whole mount (Fig. 1). There appears to be little major branching of the vessels; however, small vessels

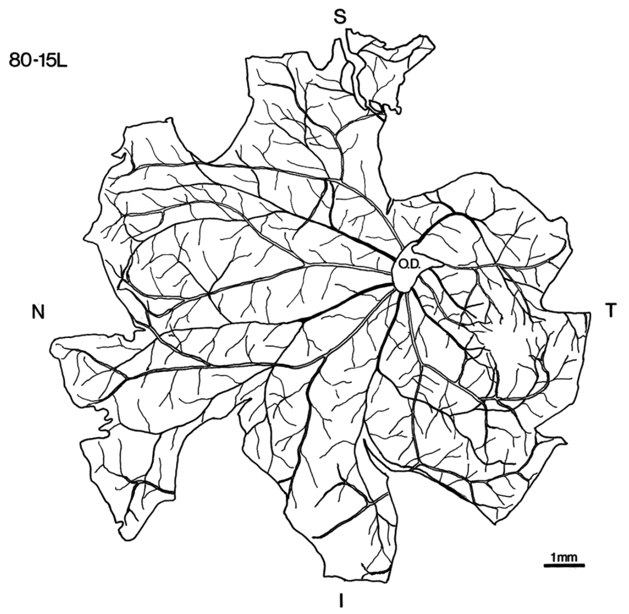

Figure 1: The pattern of major blood vessels in the tree shrew retina. The filled vessels are veins, the open ones, arteries. The area centralis is indicated by the thinning of major vessels in the temporal retina. I - inferior; N - nasal; S - superior; T temporal; O.D. - optic disc.

frequently emerge from the main ones at approximately 45° angles. The only hint of an area of specialization occurs in the temporal retina where the major vessels curve away from, and then converge upon, an area approximately 2 mm from the temporal edge of the retina and 1 mm below the optic disc. This location would be the predicted central vision representation or *area centralis* based upon electrophysiological mapping of visual cortex (Kaas et al., '72). Additionally, several smaller vessels (usually 3) proceed straight infero-temporally from the disc to this area. Although all vessels thin out as they approach the *area centralis*, examination at higher magnification reveals no totally avascular area as has been reported for the macula of the macaque (Ferraz de Olivera and Ripps, '68). Another noticeable feature of the retina is the presence of a thick layer of optic fibers which is most prominent in the

temporal retina. As in other species, the fibers proceed from the central region to the optic papilla in sweeping arcs, avoiding the area of highest cell density.

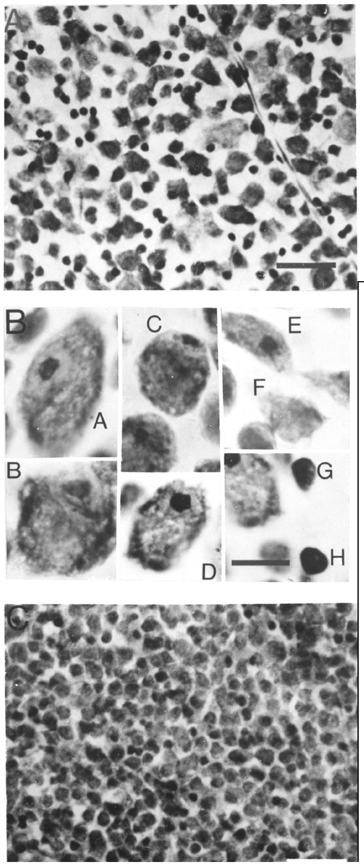

Figure 2: Photomicrographs of ganglion cells in the tree shrew retina. A: A low power photomicrograph of the ganglion cell layer in the periphery of the retina. Note the even in this eccentric location (temporal retinal margin), the density of ganglion cells is still high. Scale = 40 μ. B: High power photomicrographs of the various types of ganglion cells in the ganglion cell layer. A and B are examples of large ganglion cells with distinct nuclei, nucleoli and Nissl substance. C, D, E and Fare small ganglion cells in which the nuclear regions are less distinct. G and H are glial cells. Note the more homogeneous cytoplasm and relative absence of cytological features of the glial cells. Scale~ 10 μ. C: a low power photomicrograph of the area centralis. Note the homogeneity and small size of the ganglion cells in comparison with those in figure 2A. Scale = 40 μ.

At low magnification, the ganglion cell layer appears very crowded, with large numbers of tightly packed cells present in even the most peripheral regions (Fig. 2A). Multiple cell layers first appear at a density of approximately 9,000 cells/mm^2 and reach a maximum of three layers thick in the *area centralis* (an area delineated by the 16,000 cell/mm^2 isodensity line, extending from 0.8 mm nasal to 0.3 mm temporal and 0.3 mm superior to 0.4 mm inferior to the point of highest cell density). At higher magnification (Fig. 2B), it is evident that there are two cytologically distinct cell types present in the peripheral retina: large cells (composing the upper 25% of the local distribution of cell sizes) with distinct nuclei, nucleoli and Nissl substance, and small cells in which these cytological features are not as evident. The distinction between these smaller cells and glial cells present in the ganglion cell layer is not always clear; however, the two types can usually be differentiated on the basis of Nissl substance in the ganglion cells. As the *area centralis* is approached, the cytological distinction between large and small cells becomes less obvious until it disappears at a point approximately 0.4 mm (approximately 5°) eccentric

from the point of highest cell density. Within this central-most area, the ganglion cells appear as *a* continuous mosaic of homogeneous size profiles (Fig. 2C). The absence of large cells from the area of fixation is a feature which is also found in the primate retina (Webb and Kaas, '76; DeBruyn et al., '80, '82; Stone and Johnson, '81), although not in the cat (Stone, '65, '78; Hughes, '75, '81).

Ganglion Cell Topography

A map of ganglion cell densities within retina 80-15L is shown in Figure 3. In this figure, each line represents a zone of equal cell density or isodensity. Figure 3 also shows the same map represented in three dimensions with density represented in the third dimension. This format gives a better visual impression of the centroperipheral density gradient, the extent of the visual streak (defined peripherally by the 9,000 cell/mm^2 isodensity line and centrally by the 16,000 cell/mm^2 isodensity line) and the

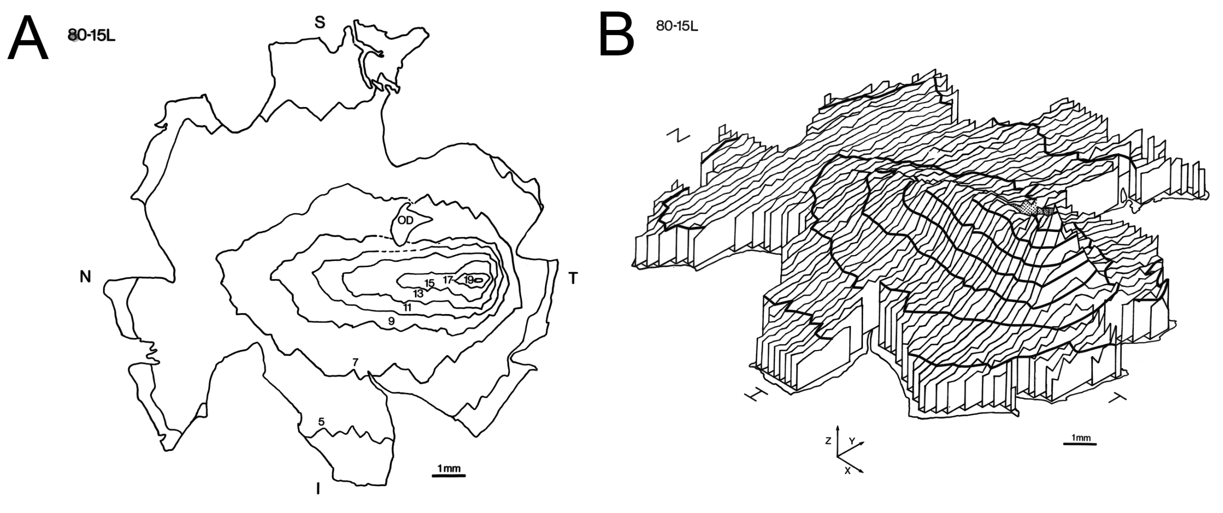

Figure 3: A: A conventional isodensity map of the tree shrew retina. The isodensity lines are drawn so that each encloses all areas in which the counts were greater than or equal to the value assigned to the line. Note the prominent visual streak (defined by the 9,000 cell/mm^2 line), nasally and the area centralis in the temporal retina. Values for each line indicated are in units of 1000 cells/mm^2. B: A three-dimensional isodensity map of the same retina. In this case, the density of cells is represented in the Z-dimension of the map and the isodensity lines are constructed so that they intersect each graph at the indicated density level. Note that density declines at a faster rate in temporal than in nasal retina. Values for the lines are the same as in the upper figure. Abbreviations as in previous figures.

density fluctuations between isodensity lines than does a conventional two-dimensional isodensity map. In both maps, the isodensity lines form a series of concentric ellipsoids, with an area of peak density (center of the *area centralis*) occurring at a point 2.2 mm from the temporal margin of the retina. Even in areas of high cell density such as the *area centralis*, there is no tendency for isodensity lines to become

circular as in the cat (Stone, '65; Hughes, '75), but to remain elliptical with a horizontal to vertical ratio of 2 to 1.

Another noteworthy feature concerns the differences in the rate of change in density in nasal and temporal retina. As can be seen in figure 3, the rate of decrease in cell density from the *area centralis* to the periphery is much higher for temporal than for nasal retina. An important implication of this trend is that points at equal eccentricities from the center of gaze have different cell densities in nasal and temporal retina.

The Visual Streak - Before proceeding further, it is necessary to define the term visual streak as presently used. The first problem is to define the peripheral boundary of the streak. In previous investigations, the peripheral boundary of the visual streak has been set somewhat arbitrarily (e.g., Hughes, '75; Oyster et al., '81) or has not been completely defined (e.g., Rowe and Stone, '76; Stone and Johnson, '81). Thus, although there is agreement that the streak is formed by isodensity lines which are horizontally elongated, the degree of this elongation has never been specified. For example, in the cat (Hughes, '75) the horizontal to vertical ratio of the visual streak is approximately 4 to l, while in the rabbit (Oyster et al., '81), it is considerably more than this. In both of these species, however, the streak shares two characteristics: first, it encloses approximately 10% of the retinal area; and second, it contains approximately 1/3 of the total number of ganglion cells. In the tree shrew, the isodensity line which matches these characteristics most closely is the 9,000 cell/mm^2 line, as it encloses approximately 12% of the retinal surface and contains approximately 30% of the total number of cells.

A second problem is to define the central boundary of the streak. As in the case of the peripheral boundary, there is no set definition for separating the streak from the more central portions of the retina (i.e., the *area centralis*). Since the streak is formed by horizontally elongated isodensity lines, while those forming an *area centralis* are thought to be more circular (Rowe and Stone, '76), the point at which there is a clear reduction in the horizontal to vertical ratio of the isodensity lines (16,000 cell/mm^2 line) was chosen as the central boundary of the streak.

The selection of these boundaries allows a lower limit to be set on the degree of horizontal elongation required for the presence of the visual streak. In the tree shrew, all isodensity lines within the streak have a horizontal to vertical ratio of approximately 3 to 1 (range 2.83 to 3.37 to 1) while the maximum ratio of any other line is less than 2.5 to 1 (range = 1.2 to 2.4 to 1). Thus, a conservative estimate for the minimum horizontal to vertical ratio of isodensity lines within the visual streak of the tree shrew would be 2.6 to 1.

Individual Variation - The ratio of horizontal to vertical dimensions of the streak can be used as a measure of individual variation in cell distribution. Figure 4 shows that the distribution of ganglion cells varies both within and between individuals. As can be seen in this figure, there is a closer correspondence between retinae from the same individual than between those of different individuals; variation is considerably less when the ratios of two retinae from the same animal are compared (79-16 - .11; 79-23 -

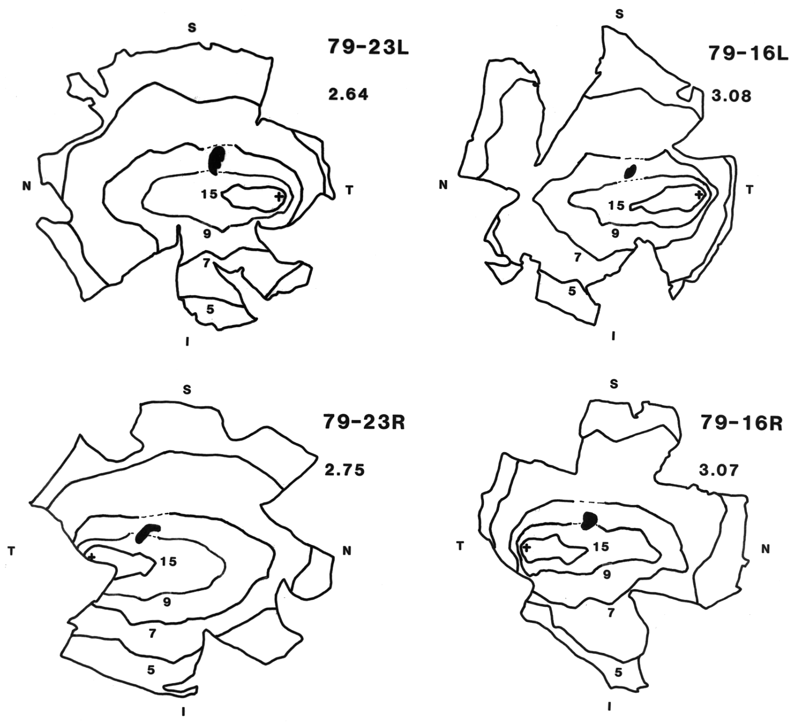

Figure 4: The intra- vs. interindividual variation in ganglion cell distribution. Note that the horizontal to vertical ratios of the visual streaks (indicated under the label of each retina) are more similar in two retinae from the same individual than in retinae from different individuals. Due to the low magnification factor of this figure, only four isodensity lines are represented in each retina. The position of the area centralis is indicated by the cross in each case, and the optic discs are filled. Other conventions as in figure 3. Abbreviations as in previous figures.

.01) than when ratios of retinae from these two individuals are compared (right retinae - .32; left retinae - .44).

Analysis of Cell Sizes

Cell areas ranged from a minimum of 20 μm^2 in the area centralis to a maximum of 550 μm^2 in the periphery. Figures 5 and 6 show that frequency histograms of cell areas at all eccentricies form roughly unimodal curves that are composed of two components; a small-celled peak which includes 85 – 90% of the total population and a large-celled tail region which contains the remaining 10 – 15% of the cells and

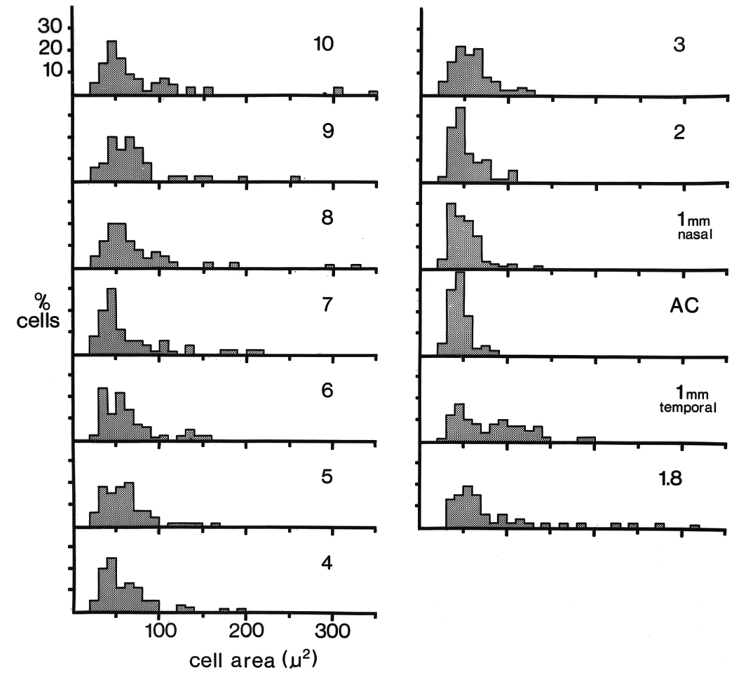

Figure 5: Histograms of ganglion cell soma sizes sampled at 1 mm intervals along the horizontal meridian. Each sample represents 150-200 cells taken from 1-4 0.1 x 0.1 mm fields. The histograms have been expressed as percentages to correct for differences in sample sizes. The eccentricity from the area centralis is indicated in the upper right of each histogram. Note that at all locations, the histograms are unimodal in form.

occupies the top 25% of the total range of ganglion cell sizes. As eccentricity from the *area centralis* increases, two trends are apparent: First, there is an increase in mean cell size (from 45 μm^2 in the *area centralis* to over 100 μm^2 in the periphery); and second, there is an increase in the proportion of cells contained within the tail region (i.e., there is an increase in variance). The rate of change in cell size is

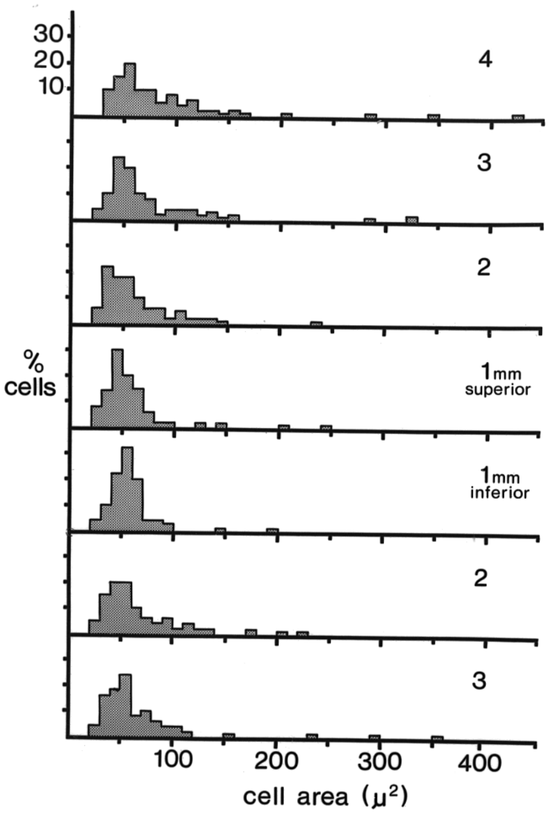

Figure 6: Histograms of ganglion cell sizes sampled at 1 mm intervals along the vertical meridian. Note that the increase in soma size is faster in this direction than along the horizontal meridian. Conventions as in the previous figure.

non-uniform with respect to the direction of sampling. Samples taken along the vertical meridian (Fig. 6) increase in size at a faster rate than do samples taken along the horizontal meridian (Fig. 5). Additionally,

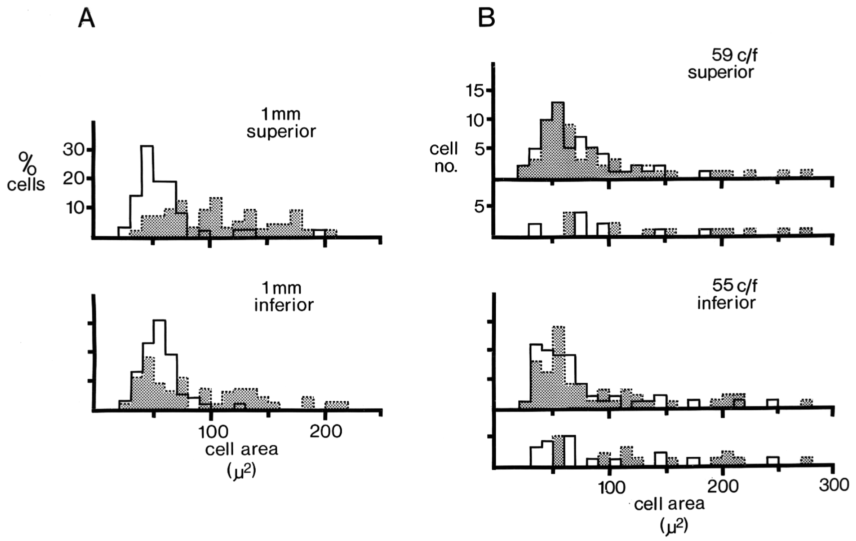

Figure 7: A: A comparison of ganglion cell soma sizes at equally eccentric locations (1 mm from the vertical meridian) in the nasal (unfilled) and temporal (filled) retina. Note that temporal ganglion cells are much larger than their nasal counterparts. As in figure 6, the results have been expressed as a percentage of total cells in order to correct for differences in density. B: A comparison of ganglion cell soma sizes at locations of equal density in the nasal (unfilled) and temporal (filled) retina. Matched samples were taken along the same elevation above and below the horizontal meridian. Below each pair of samples is a subtraction histogram in which the number of nasal cells in each bin has been subtracted from the number of temporal cells and, the excess has been plotted. Note that although the mean size of ganglion cells in nasal and temporal retina does not differ significantly, there is a greater proportion of large ganglion cells in temporal retina and of small ganglion cells in nasal retina.

figure 7A shows that ganglion cells in the temporal retina are significantly larger than nasal ganglion cells sampled at equally eccentric points from the *area centralis*, a fact which may be related to the difference in density (see above). Finally, samples taken from regions of equal cell density along the same elevation in nasal and temporal retina show no significant difference in cell size, although the percentage of large ganglion cells is greater in samples taken from the temporal retina, and the percentage of small cells is greater in the nasal retina (Fig. 7B).

Individual Variation - Distributions of cell size for matched retinal points show equivalent intra- as inter-individual variation. This is shown in Fig. 8 for density-matched samples from four different retinal regions (*area centralis*, visual streak, nasal, and temporal periphery) in 5 different retinae from 4 animals. This result contrasts with data on maps of cell density in which inter-individual variation is more pronounced.

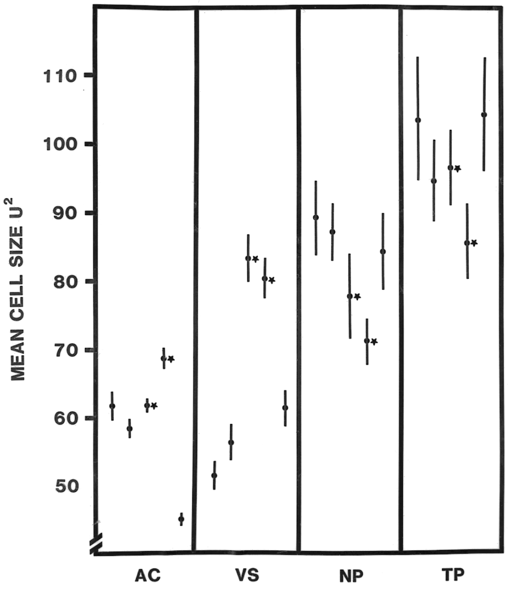

Figure 8: The intra- vs. interindividual variation in ganglion cell size. The mean size (filled circles) ± S.E.M. of density matched samples of ganglion cells from four different retinal regions (*area centralis*, visual streak, nasal, and temporal periphery) has been compared in 5 retinae from 4 different animals (131R, 3788L, 81-85R, 81-85L, 80-15L in order of appearance.). Sample densities are: AC - 20,000 cells/mm^2, VS - 15,000 cells/mm^2, NP - 7,000 cells/mm^2, TP - 5,000 cells /mm^2. Stars indicate samples taken from two retinae of the same animal. Note that the variation in the mean size of ganglion cells from two retinae of the same animal is as great as that for samples from different animals. Note also that the inverse relationship of density to ganglion cells size is not constant. For example, the mean size of cells in the visual streak of 131R is smaller than that in the *area centralis*. Finally, note the increase in the size of the S.E.M. with decreasing density. Abbreviations: AC, *area centralis*; NP, Nasal periphery; TP, temporal periphery; VS, visual streak.

DISCUSSION

The topography of the ganglion cell layer in all mammals studied, including tree shrews, is organized with a gradient in cell density and cell size such that central areas (e.g., the *area centralis* or parafovea, and the visual streak) have higher cell densities and more uniform cell sizes, generally smaller, then more peripheral retinal areas. Mammals differ in the relative location of specialized central areas, steepness of density gradients, and distribution of cell size classes within retinal areas. In tree shrews, our data show that the ganglion cell layer is characterized by a well-developed visual streak, an *area centralis* close to the temporal margin, and no fovea. Both the *area centralis* and the visual streak contain a high density of smaller cells. Cell density declines while mean soma size and range of soma sizes increases in the periphery (more rapidly for temporal than for nasal retina) but remains surprisingly high even at the extreme margins of the retina. The ensuing discussion will consider the functional implications of each of these features and how each compares with features of the retinal ganglion cell layer described for primates and other mammals.

Centro-peripheral Differences in Retinal Topography

As in most other mammals studied retinal ganglion cells in the tree shrew retina exhibit a gradient in density and cell size, being smaller, more homogeneous, and more tightly packed in the *area centralis*; and larger, more heterogeneous, and more loosely packed in the periphery. In other mammals this trend appears to follow a density gradient laid down in the receptor layer (Osterberg, '35; Steinberg et al., '73; Ogden, '75) which is repeated in other neuronal layers of the retina (e.g., Wässle et al., '78). Although less is known about the receptor density trends in tree shrews, there is evidence to suggest that the receptors in tree shrews also follow similar trends to ganglion cells since peripheral receptor to ganglion cell ratios are described as 1.6:1 (Rohen and Castenholtz, '67).

A comparison of mammalian retinae suggests that although there is a common trend toward declining ganglion cell density as one moves toward the periphery, rates of decline differ regionally within the retina (i.e., nasal-temporal differences) and greatly among species. In the tree shrew**,** the centroperipheral gradient is only 4 or 5 to 1 in contrast to gradients of 30 or 50 to 1 for nocturnal species

with wide binocular overlap, such as the cat (Stone, '65, '78; Hughes, '75, '77), owl monkey (Webb and Kaas, '76), galago (DeBruyn et al., '80), and slow loris (Debruyn et al., '82); and 300 to 1000 to 1 in diurnal primates (DeBruyn et al., '82). It is noteworthy that animals with laterally placed eyes, such as rabbits, squirrels, hamsters, mice, and rats exhibit a shallow gradient (Hughes, 71, '77; Tiao and Blakemore, '76; Fukuda, '77; Provis, '79; Oyster et al., '81; Dräger and Olson, '81) similar to the tree shrew, suggesting a greater overall dependence on peripheral vision than is the case for cats or primates. In the tree shrew, however, the absolute density is much higher than is found in most other mammals with laterally placed eyes. This adaptation is also found in diurnal squirrels (Hughes, '77) and may reflect a need for increased resolution and an overall greater dependence on vision in diurnal arboreal habitats.

It has been argued that the overall high ganglion cell density and a smaller centro-peripheral gradient, such as exists in tree shrews, are characteristics of mammals with “universal macularity", suggesting that they maintain high resolution throughout their field of view (see Hughes, '77 for review). This idea is misleading on two counts. First, it is clear that, even in tree shrews, the *area centralis* has at least four or five times the theoretical resolving power possible in the extreme periphery. Second, in animals with small eyes, a minimum number of ganglion cells in the periphery is required if any detail, at all, is to be resolved. In tree shrews, the theoretical peripheral limit of resolution (see also below), would be around two cycle/deg. This is, of course, better than would be found at the extreme margins of the retinae of mammals with steep centro-peripheral cell density gradients, such as cats and primates, in spite of the larger eye sizes, and may be an advantage in detecting potential predators.

With respect to centro-peripheral variation in cell size, studies in a number of species have demonstrated that the increases in mean cell size and the variance of cell sizes with eccentricity can be attributed to two factors. First, there is a general increase in the size of ganglion cells, regardless of functional subclass. Second, there is a change in the relative proportions of the functional subclasses which compose the local population so that the relative proportions of large, medium, and small cells, each of which reflect a different functional class, change (see Perry, '82; Rodieck and Brening, '83). Since the sizes of tree shrew ganglion cells change in a like manner, it seems reasonable to assume that similar trends are occurring (see also DeBruyn and Casagrande, '86; DeBruyn et al., '86).

Two additional related points are relevant to observations on cell size. First, most mammals studied, including tree shrews, show unimodal distributions of size at all topographic points within the retina (e.g., Oyster et al., '81; Stone and Johnson, '81. This means that meaningful correlations between cell size and functional class can only be made by considering size relative to the local distribution within confined regions; absolute differences in size are, for all practical purposes, meaningless. If size does correlate with functional class in tree shrews, then central-peripheral differences in the range of cell sizes suggest that one or a few of the functionally defined classes of ganglion cells in tree shrews are represented in the *area centralis*, while an increasing variety are represented at more peripheral locations (Van Dongen et al., '76). Second, it is clear that although, in general terms, ganglion cell size increases with decreasing cell density, it is probably more accurate to say that the distribution of relative cell sizes at a particular locus shows a consistent inverse relationship to density, being narrow at central locations and broader in the periphery. This is because when individual points are considered (even within the same retina), the inverse relationship between size and density may break down. An example of this can be seen in Figure 7 (retina 131R) where the mean size of ganglion cells from the visual streak (15,000 cells/mm^2) is smaller than the mean for cells from *area centralis* (20,000 cells/mm^2).

Although the trends described above apply generally to all centro-peripheral gradients in ganglion cell density and size composition, regional differences are also apparent, the most striking being between nasal and temporal retina or the retinal region lying temporal to the center of the *area centralis*. Our results indicate that there are two principal differences between nasal and temporal retina in tree shrews. First, the centroperipheral density gradient, particularly outside of the *area centralis*, is much steeper for temporal retina, and consequently, mean cell size also increases at a faster rate. Second, the composition of cell size classes in temporal retina differs such that, even at points of equal density, the relative percentage of large cells is greater and the overall size distribution is broader than in nasal retina. Similar nasal-temporal retinal differences have been noted in other mammals including brush-tailed possum (Freeman and Tancred, '78), rabbit (Provis, '79; however, see Oyster et al., '81), grey fox (Rapaport et al., '78), dog (Osmotherly, '79), and some primates (Stone and Johnson, '81), suggesting that this type of regional specialization may have a wide generality.

It is apparent that if ganglion cell density relates directly to visual acuity, then acuity in nasal retina will be higher than that in temporal retina for points of equal eccentricity. However, resolution of fine detail may be determined only by certain functional cell classes (e.g., Cleland et al., '71). Thus, an accurate assessment of nasal-temporal functional differences may only be determined by an examination of the relative distribution of physiological cell classes at equally eccentric locations. Differences in cell size distribution suggest that such differences in the composition of physiological cell classes exist. By analogy to work in the cat, cell size distribution differences in tree shrew would imply that, at corresponding locations, there are proportionally more Y-cells and fewer W-cells in temporal than in nasal retina (Stone, '78).

Taken together, these results strongly suggest that there are fundamental differences in the visual information that is processed by the two retinal regions. If this is the case, it is conceivable that nasal and temporal retina analyze different components of a visual stimulus (such as pattern or movement), and that the traditional view of the visual system merging identical images from each eye should be modified.

Central Areas of Specialization

Central areas of specialization within the ganglion cell layer are typically defined by high cell densities, although other criteria, such as blood vessel patterns, distribution of cell sizes, and functional classes, have also been used to define these regions (see Rapaport and Stone, '84 for review of this issue). Based upon ganglion cell density gradients, three types of retinal specialization can be identified. The first of these is evident in some nocturnal mammals such as mice (Dräger and Olson, '81), rats (Fukuda, '77), hamsters (Tiao and Blakemore, '76), and opossums (Hokoc and Oswald-Cruz, '79; Rapaport et al., '81). The pattern in these species is characterized by a shallow decline of ganglion cell density away from a central zone of increased density. The central zone of higher density, however, is not equivalent to an *area centralis* since it represents part of the monocular, not the binocular segment of visual space and, therefore, represents a specialized zone concerned with monocular vision (Rapaport and Stone, '84; Jeffery, '85). A second cell dense central zone of specialization is seen in some diurnal or crepuscular· mammals, such as grey and ground squirrels, and rabbits (Davis, '29; Hughes, '71; Oyster et al., '81; Long

and Fisher, '83; but see also Provis, '79 for rabbit). These mammals show a similar shallow decline from a central zone of high ganglion cell density except that the latter zone is horizontally elongated (a visual streak), and isodensity lines of declining value are also horizontally elongated. Like the central zone of high ganglion cell density described above for some nocturnal rodents, the visual streak is a specialized region within nasal retina and may be more concerned with monocular viewing. The third, and most commonly observed, zone of specialization is one in which a zone of higher ganglion cell density can be recognized with the area of retina represented at the center of binocular visual field; a zone which we will define here as a true *area centralis*. The *area centralis* is typically represented as a ganglion cell peak located at the temporal boundary of a variably developed visual streak. An *area centralis* exists not only in tree shrews, but also in carnivores (Stone, '65; '78; Hughes, '75, '77; Hebel, '76; Osmotherly, '79), primates (van Buren, '63; Webb and Kaas, '76; DeBruyn et al., '80, '82; Stone and Johnson, '81), ungulates (Hughes and Whitteridge, '73; Hebel, '76: Hughes, '77), and some marsupials (Freeman and Tancred, '78; Tancred, '81).

If one considers each of the above-described types of central specialization as a reflection of differential adaptations of the retinal mosaic to separate aspects of an animal's lifestyle, then the organization of retina in tree shrews suggests that it supports at least two roles since both the *area centralis* and visual streak are well developed. In fact, if one considers the marked nasal-temporal difference as another type of regional specialization, one could argue that topography of the retina in tree shrews supports three separate functional adaptations. Roles of each region of specialization are, in part, suggested by their location, cell size composition, and central targets.

The tree shrew *area centralis* is located in the center of the binocular fixation axis. It is almost devoid of blood vessels and contains the densest population of ganglion cells of uniformly small size. It has long been argued that these characteristics in other mammals such as cats, allow for binocular centering of fixation, higher spatial resolution, and possibly stereopsis (Hughes, '77). Personal observations of tree shrews suggest that they face forward to inspect objects of interest, as would be expected in order to align information with both *area centrales*. At present, it is unclear if tree shrews possess stereopsis. However, assuming that tree shrews at least use the area centralis for obtaining finer

resolution, then it is theoretically possible to predict their acuity based upon cell density, according to Shannon's sampling (Goldman, '68), since receptor to ganglion cell ratios are nearly 1:1. Using a mean retinal circumference of 13 mm (average of 5 retinae) and a visual field extent of 180 degrees (Lane et al., '71), a magnification factor of 0.07 mm/degree is obtained. Assuming that the peak cell value of (20,200 cells/mm^2) is representative, then an area of one square degree would intersect the receptive fields of 99 retinal ganglion cells (20,2000 cells/mm^2 x (0.07 mm/$degree^2$) = 99 cells/square degree), and a line one degree long would intersect the fields of 10 cells. This value corresponds to a cutoff frequency of 5 cycles/degree or a minimum resolvable bar width of 6 minutes of arc. This value is in good agreement with the figure of around 3 cycles/degree reported behaviorally by Petry et al. ('84) for tree shrews using extrapolation from contrast sensitivity functions. However, it is noteworthy that in order to arrive at the behavioral acuity of 1 minute of arc reported for tree shrews by Ordy and Samovajski ('68) tree shrews would have to possess a peak density of 735,000 cells/mm^2 , or roughly 110 layers of retinal ganglion cells.

Assuming that the tree shrew's *area centralis* is adapted to provide high resolution binocular information, then what additional information is provided by the possession of an elongated adaptation for monocular viewing such as the visual streak? Answers to this question have been sought in correlating lifestyle with the possession of a streak in mammals or birds who possess a similar adaptation (the linear area) (see Meyer, '77 and Hughes, '77 for review). Visual streaks have been identified in both nocturnal and diurnal species, as well as predators and prey. In general, this specialization appears to be associated with birds and mammals that inhabit open spaces seeking food on the ground. From the latter observation, it has been argued that the visual streak may help in detection of objects on the horizon (the terrain theory), in animals that remain in a more or less fixed relationship to this landmark, i.e., on the ground (Hughes, '77). However, the existence of the visual streak in tree shrews and primates (Stone and Johnson, '81; DeBruyn et al., '82) is inconsistent with this theory since tree shrews are mostly, although not exclusively, arboreal and reside in tropical forests where dense foliage would interfere with a view of the horizon. A second theory on the function of the visual streak suggests that it enhances the ability of an animal to detect weak visual cues (Rowe and Stone, '76). This might hold for nocturnal carnivores such as

the cat but does not fit with the strictly diurnal lifestyle and nearly all cone retina of tree shrews (Immel and Fischer, '82). A third possibility that has been suggested to explain this specialization in birds is that it may aid in the stabilization of the visual field for detection of movement while the bird is in motion (Meyer, '77). This idea would fit well with the lifestyle of the tree shrew since tree shrews make extremely rapid adjustments of head and body in space, both in relation to stationary objects and small moving prey (winged insects) or predators (human with a large net). Moreover, the tree shrew superior colliculus, which may be involved in processing such information, also is well developed and shows a retinotopically expanded representation of the visual streak (see Fig. 6, Lane et al., '71).

Is the tree shrew retina primate-like?

The relative development of the visual system played a key role in arguments favoring the inclusion of the Tupaids within the primate order (LeGros Clark, '59). In partial support of this argument, LeGros Clark ('59) emphasized the similarity between the vascularity pattern of the tree shrew retina, in which blood vessels are nearly absent from the *area centralis*, and that of primates. However, this vascularity pattern is seen in many other mammals with a well-developed *area centralis* and does not, in and of itself, support such an evolutionary relationship. In fact, the lack of a fovea, shallow centroperipheral cell density gradient, well developed visual streak, and virtual absence of rods, with no corresponding evidence of a scotopic function in tree shrews would suggest that these mammals developed rather different visual adaptions from those typical of primates. Moreover, in primates, particularly diurnal primates, the major emphasis of the retina is upon binocular vision as attested to by the sharp cell density drop-off outside of the parafovea or *area centralis*. In contrast, in tree shrews, the monocular visual specializations, (i.e., the visual streak and shallow cell density gradients), dominate the retina. It could be argued that such monocular specializations would be expected in the lateral-eyed mammals. However, as discussed above, lateral eyed mammals such as mice and rats (Dräger and Olson,'81; Fukuda, '77), lack a visual streak and, in some cases, exhibit other forms of central specialization. If one adds to these observations the fact that many details of the organization of central visual targets of the retina of tree shrews show major differences from those described in primates and that a percentage of ganglion cells in tree shrews exhibit physiological properties such *as* direction and orientation selectivity not found in

primates (Van Dongen et al., '76), it is evident that the few similarities that exist between the retinal organization of tree shrews and primates could easily be attributed to evolutionary convergence.

ACKNOWLEDGEMENTS

We are grateful to Drs. Sherre Florence and Judy Brunso-Bechtold for helpful suggestions and for reading the manuscript at different stages of completion. We thank Drs. Elizabeth Birecree and Lance Durden for technical help in various stages of this study, Aurora Buck for her skillful care and handling of the animals, Julie Mavity for photographic assistance, Vera Murphy for typing and to Sam Marvin for proofing the manuscript.

This research was supported by Public Health Service Grant EY017778 and was performed in partial fulfillment of the requirements for the degree of Doctor of Philosophy from Vanderbilt University. E.J. DeBruyn was supported by Public Health Service Fellowship MH08472 and by Neuroscience Training Grant MH15452. V.A. Casagrande was supported by Research Career Development Award K04-EY00223.

REFERENCES

Albano, J.E., A.L. Humphrey, and T.T. Norton 1978 Laminar organization of receptive field properties in the tree shrew superior colliculus. J. Neurophysiol., 41:1140-1164.

Albano, J.E., T.T. Norton, and W.C. Hall 1979 Laminar origins of projections from the superficial layers of the superior colliculus in the tree shrew, *Tupaia glis*. Brain Res., 173:1-11.

Boycott, B.B., and H. Wässle 1974 The morphological types of ganglion cells in the domestic cat's retina. J. Physiol. (Lond.), 240:397-419.

Brecha, N., and H.H. Karten 1979 Accessory optic projections upon oculomotor nuclei and vestibulo cerebellum. Science, 203:913-916.

Bunt, A.H., A.E. Hendrickson, J.S. Lund, R.D. Lund, and A.F. Fuchs 1975 Monkey retinal ganglion cells: Morphometric analysis and tracing of axonal projections with a consideration of the peroxidase technique. J. Comp. Neural., 164:265-286.

Carey, R.G., D. Fitzpatrick, and I.T. Diamond 1979 Layer I of striate cortex in *Tupaia glis* and *Galago senegalensis*: projections from thalamus and claustrum revealed by retrograde transport of horseradish peroxidase. J. Comp. Neural., 186:393-439.

Casagrande, V.A., and J.K. Harting 1975 Transneuronal transport of tritiated fucose and proline in the visual pathways of the tree shrew, *Tupaia glis*. Brain Res., 96(2):367-373.

Clark, W. E. LeGros 1959 The antecedents of man. Edinburgh Univ. Pres, Edinburgh.

Cleland, B.G., M.W. Dubin, and W.R. Levick 1971 Sustained and transient neurons in the cat's retina and lateral geniculate nucleus. J. Physiol. (Land.), 217:473-496.

Cleland, B.G., W.R. Levick, and H. Wässle 1975 Physiological identification of a morphological class of cat retinal ganglion cell. J. Physiol. (Land.), 248:151:171.

Conley, M., D. Fitzpatrick and I.T. Diamond 1984 The laminar organization of the lateral geniculate body and the striate cortex in the tree shrew (*Tupaia glis*). J. Neurosci. 4: 171-197.

Davis, F.A. 1929 The anatomy and histology of the eye and orbit of the rabbit. Am. Ophthal. Soc. Trans., 27:401-441.

DeBruyn, E.J., and V.A. Casagrande 1980 Tree shrew retinal ganglion cells: Differences in nasal and temporal retina. Soc. for Neurosci. Abstr., 6:348.

DeBruyn, E., and V.A. Casagrande 1978 Density and central projections of retinal ganglion cells in the tree shrew. Soc. for Neurosci. Abstr., 4:624.

DeBruyn, E.J., V.W. Wise, and V.A. Casagrande 1980 The size and topographic arrangement of retinal ganglion cells in the Galago. Vision Res., 20:314-327.

DeBruyn, E.J., J. Tigges, and V.A. Casagrande 1982 Variations in primate retinal ganglion cell organization. Invest. Ophthal. Vis. Sci. Suppl., 22:246.

DeBruyn, E.J., and V.A. Casagrande 1986 The organization of retinal ganglion cells in the tree shrew (*Tupaia belangeri*): II. Analysis of Cell Morphology. J. Comp. Neural. (Submitted).

DeBruyn, E.J., J.T. Weber, and V.A. Casagrande 1986 The organization of retinal ganglion cells in the tree shrew (*Tupaia belangeri*): III. Central Projection of Ganglion Cells. J. Comp. Neural. (Submitted).

Dräger, U.C. and J. F. Olsen 1981 Ganglion cell distribution in the retina of the mouse. Invest. Ophthal. Vis. Sci. 20: 285-293.

Ferraz de Oliveria, L., and H. Ripps 1968 The "area centralis" of the owl monkey (*Aotus trivirgatus*). Vision Res., 8:223-228.

Freeman, B., and E. Tancred 1978 The number and distribution of ganglion cells in the retina of the brush-tailed possum, *Trichosurus vulpecula*. J. Comp. Neural., 177:557-568.

Fukuda, Y. 1977 A three-group classification of rat retinal ganglion cells: Histological and Physiological studies. Brain Res., 119:327-344.

Goldman, S. 1968 Information theory. Dover Press, New York.

Graham, J., and V.A. Casagrande 1980 A light microscopic and electronmicroscopic investigation of the superficial layers of the superior colliculus of the tree shrew (*Tupaia glis*). J. Comp. Neural., 191:133-151.

Harting, J.K., W.C. Hall, and I.T. Diamond 1972 Evolution of the pulvinar. Brain Behav. Evol., 6:424-452.

Harting, J.K., W.C. Hall, I.T. Diamond and G.F. Martin 1973 Anterograde degeneration study of the superior colliculus in *Tupaia glis*: Evidence for a subdivision between the superficial and deep layers. J. Comp. Neurol., 148:361-386.

Hebel, R. 1976 Distribution of retinal ganglion cells in five mammalian species (pig, sheep, ox, horse, dog). Anat. Embryol. (Berl.), 150:45-51.

Hokoc, J.N., and E. Oswaldo-Cruz 1979 A regional specialization in the opossum retina: Quantitative analysis of the ganglion cell layer. J. Comp. Neurol., 183:385-396.

Hubel, D.R. 1975 An autoradiographic study of the retina-cortical projections in the tree shrew (*Tupaia glis*). Brain Res., 96:41-50.

Hughes, A. 1971 Topographical relationships between the anatomy and physiology of the rabbit visual system. Docurnenta Ophthal., 30:33-160.

Hughes, A. 1975 A quantitative analysis of the cat retinal ganglion cell topography. J. Comp. Neural., 163:107-128.

Hughes, A., 1977 The topography of vision in mammals of contrasting lifestyle: Comparative optics and retinal organization. In: Handbook of Sensory Physiology, Vol. VII/5. F. Crescitelli, ed., Springer, New York.

Hughes, A., 1981 Population magnitudes and distribution of major modal classes of cat retina ganglion cells as estimated from HRP filling and a systematic survey of the soma diameter spectra for classical neurons. J. Comp. Neural., 197:303-339.

Hughes, A., and D. Whitteridge 1973 The receptive fields and topographical organization of goat retinal ganglion cells. Vision Res., 13:1101-1114.

Hughes, A., and D.I. Vaney 1980 Coronate cells: displaced amacrines of the rabbit retina? J. Comp. Neural., 189:169-189.

Hughes, A., and E. Weiniawa-Narckiewicz 1980 A newly identified population of presumptive "microneurons" in the cat retinal ganglion cell layer. Nature, 284:468-470.

Immel, J.H., and S.V. Fischer 1982 Tree shrew cones shed when the lights come on. Invest. Ophthalmol. Vis. Sci. Suppl., 22:140.

Jeffery, G. 1985 The relationship between cell density and the nasotemporal division in the rat retina. Brain Res., 347:354-357.

Kaas, J.H., W.C. Hall, H. Killackey, and I.T. Diamond 1972 Visual cortex of the tree shrew (*Tupaia glis*): Architectonic subdivisions and representations of the visual field. Brain Res., 42:491-496.

Karten, J.H., K.V. Fite, and N. Brecha 1977 Specific projection of displaced retinal ganglion cells upon the accessory optic system in the pigeon (*Columbia livia*). Proc. Nat. Acad. Sci. U.S.A., 74(4):1753-1756.

Kimm, J., J.A. Winfield, and A.E. Hendrickson 1979 Visual-vestibular interactions and the role of the flocculus in the vestibulo-ocular reflex. Prog. Brain Res., 50:703-713.

Laemle, L.K. 1968 Retinal projections of *Tupaia glis*. Brain Behav. Evol., 1:473-499.

Lane, R.H., J.M. Allman, and J.H. Kaas 1971 Representation of the visual field in the superior colliculus of the grey squirrel (*Sciurus carolinensis*) and the tree shrew (*Tupaia glis*). Brain Res., 26:277-292.

Long, K.O. and S.K. Fisher 1983 the distribution of photoreceptors and Ganglion cells in the California Ground Squirrel, *Spermophilis beecheyi*. J. Comp. Neural., 221:329-340.

Meyer, D.B. 1977 The avian eye and its adaptations. In: *Handbook of Sensory Physiology*. Vol. VII/5. F. Crescitelli, ed., Springer, New York.

Ogden, T.E. 1975 The receptor mosaic of *Aotus trivirgatus*: Distribution of rods and cones. J. Comp. Neural., 153:399-428.

Ohno, T., U. Misgeld, S.T. Kitai, and A. Wagner 1975 Organization of visual afferents into the LGd and the pulvinar of the tree shrew, *Tupaia glis*. Brain Res., 90:153-158.

Ordy, J.M., and T. Samovajski 1968 Visual acuity and ERG-CFF in relation to the morphological organization of the retina among diurnal and nocturnal primates. Vision Res., 8:1205-1225.

Osmotherly, S. Retinal topography of the dog (*Canis familaris*). Proc. Aust. Physiol. Pharmacol. Soc., 10:143P.

Osterberg, G. 1935 Topography of the layer of rods and cones in the human retina. Acta Ophthalmol. Kbh., Suppl. 6.

Oyster, C.W., E.S. Takahashi, and D.C. Hurst 1981 Density, soma size and regional distribution of rabbit retinal ganglion cells. J. Neurosci., 1:1331-1346.

Perry, V.H. 1982 The ganglion cell layer of the mammalian retina. In *Progress in Retinal Research* (eds. Osborn, N., and Chades, G.), Vol. l, pp. 53-80. Pergamon Press, Oxford.

Petry, H.M., R. Fox, and V.A. Casagrande 1984 Spatial contrast sensitivity of the tree shrew. Vision Res., 24:1037-1042.

Provis, J.M. 1979 The distribution and size of ganglion cells in the retina of the pigmented rabbit: A quantitative analysis. J. Comp. Neural., 185:121-137.

Rapaport, D.H. and J. Stone 1984 The *area centralis* of the retina in the cat and other mammals: Focal point for function and development of the visual system. Neurosci. 11(2):289-301.

Rapaport, D.H., M.A. Sesma, and M. Rowe 1978 Distribution and central projections of ganglion cells in the retina of the gray fox (*Urocyon cinereoargenteus*). Soc. for Neurosci. Abstr., 5:804.

Rapaport, D.H., P.D. Wilson, and M.H. Rowe 1981 The distribution of ganglion cells in the retina of the North American opossum (*Didelphis virginiana*). J. Comp. Neurol., 199:465-480.

Rodieck, R.W. and R.K. Brening 1983 Retinal ganglion cells: properties, types, genera, pathways and trans-species comparisons. Brain Behav. Evol., 23:121-164.

Rohen, J.W., and A. Costenholtz 1967 Uber die zentralisation der retina bei Primaten. Folia Primat., 5:92-147.

Rowe, M.H., and J. Stone 1976 Properties of ganglion cells in the visual streak of the cat's retina. J. Comp. Neurol., 169:99-126.

Saito, H. 1983 Morphology of physiologically identified X-, Y- and W-type retinal ganglion cells of the cat. J. Comp. Neurol., 221:279-288.

Sherman, S.M., T.T. Norton, and V.A. Casagrande 1975 X- and Y-cells in the dorsal lateral geniculate nucleus of the tree shrew (*Tupaia glis*). Brain Res., 93:152-157.

Stanford, L.R., and Sherman, S.M. 1984 Structure/function relationships of retinal ganglion cells in the cat. Brain Res., 297:381-386.

Steinberg, R.H., M. Reid, and P. Lacy 1973 The distribution of rods and cones in the retina of the cat (*Felis domesticus*). J. Comp. Neurol., 148:229-248.

Stone, J. 1965 A quantitative analysis of the distribution of ganglion cells in the cat's retina. J. Comp. Neurol., 124:337-352.

Stone, J. 1978 The number and distribution of ganglion cells in the cat's retina. J. Comp. Neurol., 180:753-772.

Stone, J., and E. Johnson 1981 The topography of primate retina: A study of the human, bushbaby, and New- and Old-World monkeys. J. Comp. Neurol., 196:205-223.

Stone, J., B. Dreher, and A.G. Leventhal 1979 Parallel and hierarchical mechanisms in the organization of visual cortex: A review. Brain Res. Rev., 1:345-394.

Tancred, E. 1981 The distribution and sizes of ganglion cells in the retinas of five Australian marsupials. J. Comp. Neurol., 196:585-603.

Tiao, Y.-C., and C. Blakemore 1976 Regional specialization in the golden hamster's retina. J. Comp. Neural., 168:439-458.

Tigges, J., B.A. Brooks and M.R. Klee 1967 ERG recordings of a primate pure cone retina (*Tupaia glis*). Vision Res., 7:553-563.

Van Buren, J.M. 1963 The retinal ganglion cell layer. Charles Thomas, Springfield, Ill.

Van Dongen, P.A., H.J. Terlaak, J.M. Thijssen, and A.J.H. Vendrik 1976 Functional classification of cells in the optic tract of a tree shrew (*Tupaia glis*). Exp. Brain Res., 24:441-446.

Wässle, H., W.R. Levick, and B.G. Cleland 1975 The distribution of the alpha type of ganglion cells in the cat's retina. J. Comp. Neurol., 159:419-438.

Wässle, H., L. Peichl, and B.B. Boycott 1978 Topography of horizontal cells in the retina of the domestic cat. Proc. Roy. Soc. (Lond.) B, 203:269-291.

Webb, S.V., and J.H. Kaas 1976 The sizes and distribution of ganglion cells in the retina of the owl monkey, *Aotus trivirgatus*. Vision Res., 16:1247-1254.
